# Porous Organic Frameworks Utilizing Halogen···Halogen Interactions of X4–tetra[2,3]Thienylene (X = Br, I): Guest Dynamics and Dielectric Response

**DOI:** 10.1002/chem.202502872

**Published:** 2025-11-10

**Authors:** Genki Saito, Takashi Takeda, Shun Dekura, Jumpei Moriguchi, Tetsu Sato, Ryo Tsunashima, Tomoyuki Akutagawa

**Affiliations:** ^1^ Graduate School of Engineering Tohoku University Sendai 980–8579 Japan; ^2^ Institute of Multidisciplinary Research for Advanced Materials (IMRAM) Tohoku University 2–1–1 Katahira, Aoba–ku Sendai 980–8577 Japan; ^3^ Faculty of Science Shinshu University 3–1–1 Asahi Matsumoto 390–8621 Japan; ^4^ Graduate School of Sciences and Technology for Innovation Yamaguchi University Yoshida 1677–1 Yamaguchi 753–8512 Japan

**Keywords:** dielectric behavior, dynamics, halogen interaction, host‐guest, tetra[2,3]thienylene

## Abstract

X4‐tetrabromo[2,3]thienylene (**1**, Br4‐tetra[2,3]thienylene) and X4‐tetraiodotetra[2,3]thienylene (**2**, I4‐tetra[2,3]thienylene) were used to investigate the formation of various guest inclusion crystals through intermolecular halogen···halogen (X···X) interactions and the dynamics of guest molecules. **1** and **2** formed host–guest crystals with aromatic molecules such as benzene (Bz), toluene (Tol), anisole (An), and chlorobenzene (ClBz). X‐ray crystal structure analysis revealed the formation of a channel structure where 1D chains formed by intermolecular X···X interactions were arranged in a grid pattern. Host lattices of **1** and **2** were capable of reversibly adsorbing and desorbing Tol molecules while maintaining crystallinity. Dielectric measurements showed dielectric relaxation due to molecular motion of polar guest molecules (Tol and ClBz) in the host lattice formed by molecule **2**. In particular, crystals containing ClBz exhibited rotational dynamics of guest molecules with increasing temperature. These results provide important insights for the design of flexible porous materials using halogen···halogen interactions and the creation of dielectric materials based on guest molecule dynamics.

## Graphical abstract

X4‐tetrahalo[2,3]thienylene with four halogen atoms (X = Br and I) at the molecular terminals were used to investigate the formation of various guest inclusion crystals through intermolecular halogen···halogen (X···X) interactions and the dynamics of guest molecules.

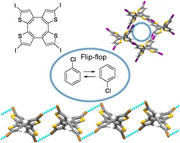

## 1 Introduction

Porous materials are widely studied for their diverse potential applications including catalysis,^[^ ^1^, ^2^, ^3^ ^]^ gas adsorption and separation,^[^ ^4^, ^5^, ^6^ ^]^ and sensing.^[^ ^7^, ^8^, ^9^, ^10^ ^]^ Conventional porous materials such as metal‐organic frameworks (MOFs) and covalent organic frameworks (COFs) have attracted attention, where these are mainly constructed through coordination bonds or covalent bonds. Meanwhile, in the recent years, porous materials utilizing noncovalent interactions, such as hydrogen‐bonded organic frameworks (HOFs), have attracted attention for their structural flexibility and reversible assembly characteristics.^[^ ^11^, ^12^, ^13^, ^14^, ^15^ ^]^ Additionally, van der Waals open frameworks (WaaFs) with superior flexibility have been developed,^[^ ^16^ ^]^ and porous materials focusing on various types of intermolecular forces are being developed.

The π‐electron organic compounds are utilized in the development of various functional materials such as conductive, magnetic, and optical materials due to their high design freedom of electronic states.^[^ ^17^, ^18^, ^19^, ^20^, ^21^, ^22^, ^23^ ^]^ In particular, nonplanar π‐electron molecules such as cyclooctatetraene (COT) and sumanene frameworks are attracting attention as foundations for various functional materials due to their unique 3D structures and electronic states.^[^ ^24^, ^25^, ^26^, ^27^, ^28^ ^]^ Compounds with hydrogen‐bonding functional groups introduced into the COT framework form intermolecular hydrogen bond networks and function as HOFs and proton conductors. For example, derivatives with carboxyl groups introduced into the COT framework form strong hydrogen bond networks, realizing stable porous structures. These materials show high gas adsorption capacity and selective molecular recognition ability,^[^ ^29^, ^30^ ^]^ making them promising for applications in environmental and energy fields. Additionally, compounds with proton‐accepting groups such as amino groups introduced into the COT framework form proton conduction pathways through hydrogen bonds, showing high proton conductivity.^[^ ^31^ ^]^ In particular, examples achieving proton conductivity (10^−4^ to 10^−6^ S cm^−1^) even under nonhumidified conditions through cooperative proton transfer (Grotthuss mechanism) within hydrogen bond networks have been reported. These materials are considered promising as electrolyte materials for the next‐generation fuel cells.

In the recent years, research on the mechanical properties of organic crystals has rapidly developed, with particular attention to organic crystals showing elastic deformation and plastic deformation.^[^ ^32^, ^33^, ^34^, ^35^, ^36^, ^37^, ^38^ ^]^ Especially, crystals constructed from 1D column structures with weak intermolecular interactions including halogen bonds have been shown to exhibit unique mechanical properties due to the balance between the directionality and strength of intermolecular interactions. In elastic crystals using halogen bonds, molecules in the crystal lattice can undergo reversible deformation where they temporarily displace under mechanical stimuli but return to their original arrangement after the stimulus is removed.^[^ ^35^, ^39^, ^40^ ^]^ This is due to the balance between the directionality and flexibility of halogen···halogen (X···X) interactions. Elastic deformation crystals containing halogen bonds are expected to be applied as phase transition materials and mechanochromic materials. Furthermore, in the recent years, material development utilizing halogen interactions has been reported not only for organic materials but also for organic‐inorganic hybrid materials.^[^ ^41^, ^42^ ^]^ Halogen···halogen interactions are a type of noncovalent interaction based on σ‐hole interactions arising from the anisotropic electron density distribution of halogen atoms.^[^ ^43^, ^44^ ^]^ In particular, heavy halogen atoms such as Br and I are known to have high polarizability and form strong directional interactions. Porous materials utilizing these halogen···halogen interactions (halogen‐bonded organic frameworks, XOFs) ^[^ ^45^, ^46^, ^47^ ^]^ can be expected to have characteristic advantages compared to MOFs, COFs, and HOFs. Halogen···halogen interactions are weaker than hydrogen bonds but have directionality, potentially allowing the structure to flexibly change in response to guest molecule incorporation. By varying the type of halogen atom from F, Cl, Br, to I, the strength and directionality of interactions can be precisely controlled, and additionally, the electronic properties of halogen atoms allow electronic adjustment of host–guest interactions.^[^ ^48^, ^49^ ^]^ Also, being relatively weak interactions, they are characterized by excellent responsiveness to external stimuli such as temperature and pressure, as shown in elastic crystals. However, most XOFs developed so far involve systems with electrostatic N − H^+^···I^−^···N intermolecular interactions between pyridinium and I^−^, and there are few reports of host–guest crystals made from neutral halogen···halogen interactions.^[^ ^48^, ^49^ ^]^

**Scheme 1:**
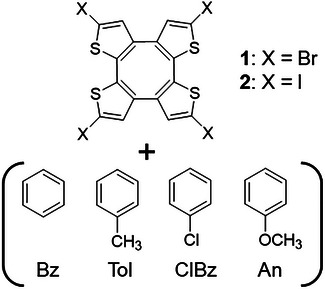
Chemical structures of X4‐tetra[2,3]thienylenes (**1** and **2**: X = Br and I) and guest molecules of benzene (Bz), toluene (Tol), chlorobenzene (ClBz), and anisole (An).

While HOFs and proton conductors utilizing hydrogen bonds are characterized by stable structures based on strong interactions and high proton conductivity, XOFs using halogen···halogen interactions are expected to have more flexible structures and dynamic/mechanical responsiveness. Considering such complementary characteristics, in this study we designed and synthesized X4–tetra[2,3]thienylene with four halogen atoms (X = Br or I) at the terminals of the COT framework, which is a nonplanar π‐electron molecule (Scheme 1). In this molecular design, the saddle‐shaped nonplanar structure of the COT framework forms effective voids in crystal packing. From X‐ray structure analysis of HOF crystals in our previous research, we have revealed that the dihedral angle of thiophene rings is about 40°,^[^ ^30^ ^]^ and this nonplanarity creates gaps between molecules, enabling the inclusion of solvent molecules. Unlike HOFs, by positioning four halogen atoms at the terminals of the nonplanar framework, a three‐dimensionally extended halogen···halogen interaction network is formed, which is expected to realize a stable porous structure Scheme 1.

By combining the π‐electron system of the COT framework with halogen atoms, it is possible to develop functional materials that combine electronic and optical properties with porous structures, while this research focuses on dielectric responses through interactions with guest molecules. In the COT framework, intermolecular S···S interactions are observed in addition to halogen···halogen interactions, which may contribute to the stabilization of the crystal structure. Since the type of halogen atom (Br and I) can precisely control intermolecular distances and channel sizes, in this study, we increased the channel size by changing Br atoms to I atoms and examined the inclusion of benzene derivatives (benzene: Bz, toluene: Tol, chlorobenzene: ClBz, anisole: An) as more diverse guest molecules. Utilizing these characteristics, this study focuses on the differences in intermolecular interactions due to the type of halogen atom (Br and I) on the COT framework and their effects on the structure and properties of host–guest crystals. In particular, by examining in detail the relationship between guest molecule dynamics and dielectric response, we report the development of novel external field–responsive dielectric materials using halogen···halogen interactions.

## 2 Results and Discussion

Compound **1** was obtained in 87% yield by treating tetratrimethylsilyltetra[2,3]thienylene with *N*‐bromosuccinimide according to existing methods.^[^ ^50^, ^51^ ^]^ Compound **2** was newly synthesized with reference to the synthesis method of compound **1** by adding *N*‐iodosuccinimide to the reaction solution. Molecules **1** and **2** were dissolved in Bz, Tol, An, and ClBz, respectively, and host–guest single crystals with various crystal formula were obtained by introducing methanol, a poor solvent, via vapor diffusion method (Table 1 and S1). Molecule **1** formed inclusion crystals with a 1:1 composition with guest molecules Bz, Tol, and An, while did not form inclusion crystals with ClBz. On the other hand, molecule **2** showed more complex behavior than **1**, forming crystals with 1:2 and 1:3 compositions with Bz, and 1:1 composition crystals with Tol, An, and ClBz. Various crystal formations were confirmed by combinations of halogen substituent types (X = Br and I) and guest solvent molecules (Bz, Tol, An, and ClBz). Single crystal X‐ray structure analysis at 100 K was conducted on these nine host–guest single crystals and the host single crystal to examine in detail the intermolecular interactions between host lattices and between host and guest (Table S1). In all crystals, 1D chain structures were formed by intermolecular Br···Br interactions in molecule **1** and intermolecular I···I interactions in molecule **2**. These 1D chains were arranged in a grid pattern through weak intermolecular interactions such as S···Br (I), Br (I)···π, and π···π, forming 1D solvent inclusion channels. Exceptionally, only the **2**·3(Bz) crystal showed a channel structure with orthogonal solvent molecule layers. Also, the crystal density of the **2**·2(Bz) crystal was significantly lower at 1.858 g cm^−3^ compared to other crystals, suggesting a loose packing style of guest molecules. Table 1 summarizes the space group, crystal density, void space volume per host molecule excluding guest molecules, and the percentage of void space in the unit cell for the obtained host–guest crystals.

**Table 1:** Classification of host–guest crystals and characteristics of packing structures.

Host·guest	Space group	*d*_calc_ [g cm^−3^]^[^ ^a^ ^]^	Void [Å^3^]^[^ ^b^ ^]^	Void [vol.%]^[^ ^c^ ^]^	Guest [vol.%]^[^ ^d^ ^]^
1·Bz	*I*2/*a*	2.010	740	31	68
1·Tol	*I*2/*a*	2.025	764	32	82
1·An	*I*2/*a*	2.071	753	31	89
2·2(Bz)	*Ibam*	1.858	1841	52	54
2·3(Bz)	*Ibam*	2.049	1767	51	84
2·Tol	*I*2/*a*	2.335	852	32	73
2·An	*I*2/*a*	2.394	837	32	80
2·ClBz	*P*2_1_/*c*	2.415	824	32	67

^[a]^ Calculated crystal density determined from single crystal X‐ray structure analysis at 100 K.

^[b]^ Volume of void space per host molecule in the unit cell.

^[c]^ Percentage of void space in the unit.

^[d]^ Percentage of void space occupied by guest molecules.

### 2.1 1D Br∼Br Chain System of **1**

**Figure 1:**
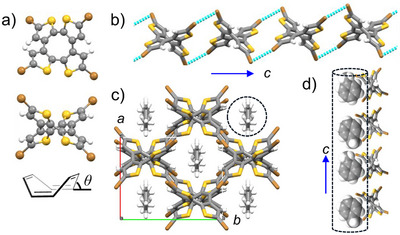
Crystal structure of **1·**Tol at 100 K. a) Molecular structure of **1** viewed along the normal direction to the COT plane (upper) and the parallel direction to the COT plane (lower). The definition of dihedral angel *θ* for molecules **1** and **2**. b) 1D array of **1** through the double Br···Br interactions along the *c* axis. c) Unit cell viewed along the *a *+ *c* axis. 1D array of guest An molecules along the *a *+ *c* axis. d) molecular arrangement of Tol along the *a *+ *c* axis using CPK model for Tol.

All crystal structures are similar to each other. Figure 1 shows the crystal structure of **1**·Tol at 100 K as a representative example. Figure 1 shows the molecular structure viewed from directions perpendicular and parallel to the COT framework at the center of molecule **1**. The four thiophene rings fused to the central COT core take a saddle‐shaped conformation pointing up and down from each other, and the dihedral angle (*θ*) representing this twist was 40.6°. For other crystals, the magnitude of *θ* was observed in the range of 40.9 to 42.2°, and the *θ* values in the optimized structures by DFT calculations for **1** and **2** were 41.4 and 41.3°, respectively (Figure S1). The fact that the calculated values for isolated molecules and the measured values in crystals showed close values revealed that the nonplanar molecules **1** and **2** do not undergo significant strain in the crystal.

Figure 1b shows the 1D chain structure formed by molecule **1** through intermolecular Br···Br interactions. The nonplanar molecule **1** formed a 1D chain along the *c*‐axis through intermolecular Br···Br interactions involving four Br groups at the molecular terminals. The observed distance between Br atoms *d*_Br–Br_ was 3.674 Å, which is shorter than the sum of the van der Waals radii of Br atoms (3.72 Å), confirming effective intermolecular interactions. Figure 1c is the unit cell viewed along the *c*‐axis of **1**·Tol. The 1D chains extended along the *c*‐axis of the unit cell and were arranged parallel in the *ac*‐plane. Furthermore, the 1D chain arrangement along the *a *+ *b* axis was twisted by 90°, realizing a grid‐like structure where 1D chains were stacked on each other. Between the 1D chains, intermolecular S···S bonds were observed at 3.756 Å, which was approximately the same distance as the sum of the van der Waals radii of S atoms (3.7 Å). Therefore, it was shown that the 1D chains were fixed as a grid‐like host lattice through intermolecular S···S interactions. This host lattice formed a channel along the *c*‐axis of the crystal, and the guest Tol molecule existed in that space (Figure 1d).

In the **1**·Tol crystal, the void space when excluding guest molecules was 764 Å^3^ relative to the unit cell, occupying 32% of the unit cell volume. Based on the molecular volume of Tol estimated by DFT calculations as 156 Å^3^, it formed a stable host–guest crystal by occupying 82% of the void space in the unit cell. Orientational disorder related to the direction of the methyl group was observed in the Tol molecule with an occupancy ratio of 0.5:0.5, and a two‐fold rotation axis existed at the center of the benzene six‐membered ring. In the channel of the crystal structure at 100 K, adjacent Tol molecules were arranged with their methyl groups alternating in direction to minimize steric repulsion, canceling out their dipole moments.

Between the host lattice formed by molecule **1** and Tol, C−H···S and C−H···π interactions were observed at distances of 2.877 to 3.096 Å, indicating that guest molecules were stably incorporated into host molecules. In fact, thermogravimetry (TG) measurements showed no weight loss up to around 380 K, confirming that guest molecules were stably retained even near room temperature (discussed later in Figure 3). The **1**·Tol crystal showed a weight loss of 12.1% when heated to 400 K, which agreed well with the theoretical weight percentage of Tol molecules (12.5%). Since the channels are separated from each other by a distance of about 8.4 Å along the *a *+ *b* axis direction, there are no interactions between guest molecules. In crystal structure analysis at 293 K, no changes in space group or molecular arrangement pattern were observed, while the average value of the anisotropic temperature factor of carbon atoms of Tol, the guest molecule, increased from 0.031 at 100 K to 0.085 at 293 K, indicating thermal fluctuations of guest molecules (Figure S3).

For molecule **1** with guest molecules Bz and An, crystal structures similar to **1**·Tol with channels were observed (Figures S4, S5). Also, molecule **1** did not form inclusion crystals with ClBz. The void space and its percentage in the unit cell of the **1**·Bz crystal were 740 Å^3^ and 31%, respectively, and for **1**·An, they were 753 Å^3^ and 31%. From the molecular volumes of Bz and An estimated by DFT calculations as 125 and 167 Å^3^, respectively, they occupied 68% and 89% of the void space. Therefore, it is considered that An interacts more strongly with the host lattice than Bz. Since Tol occupied 82% of the void space formed by **1**, the space filling rate decreased in the order An > Tol > Bz. These guest molecules were stably incorporated into the host lattice not by π–π interactions with host molecules, but by multipoint C−H···Br and C−H···S interactions. Since molecule **1** realizes similar channel structures for guest molecules of different sizes and shapes (Bz, Tol, and An), the host lattice consisting of 1D chains constructed by Br···Br interactions is considered to be rich in flexibility.

### 2.2 1D I∼I Chain System of 2

We examined the host–guest crystal structures for molecule **2**, where the Br groups at the molecular terminals of the COT framework were changed to larger I groups. The van der Waals radii of Br and I atoms are 1.85 and 2.04 Å, respectively, and I atoms have characteristics that make their electron clouds more easily polarized than Br atoms. Therefore, in intermolecular halogen···halogen interactions, I atoms are expected to form stronger halogen bonds through σ–hole interactions, showing stronger directional interactions than Br···Br interactions.^[^ ^43^, ^44^ ^]^ This is because the formation of σ–holes due to the anisotropic electron density distribution of I atoms generates stronger electrostatic interactions. In this study, we confirmed that molecule **2** with four I atoms at the molecular terminals forms crystal structures similar to molecule **1** for guest molecules Bz, Tol, An, and ClBz (Figures S6–S8). Exceptionally, it forms a 1:3 composition crystal with Bz, while this crystal is not stable at room temperature and changes to **2**·2(Bz) with a 1:2 composition. Also, it did not form host–guest crystals with fluorobenzene (FBz).

By changing Br atoms to I atoms, the 1D channels formed by the host lattice became larger in size. The observed I atom distance *d*_I–I_ was 3.875∼3.940 Å, which is shorter than the sum of the van der Waals radii of I atoms (4.08 Å), confirming effective intermolecular I···I interactions. These 1D chains extended along the *c*‐axis and were arranged parallel in the *ac* plane. Furthermore, the 1D arrangement along the *a *+ *b* axis was twisted by 90°, realizing a grid‐like structure. Between the 1D chains, unlike molecule **1**, no intermolecular S···S interactions were observed, while weak intermolecular interactions such as C−H···π and C−H···S existed. Therefore, it is considered that the 1D chains are more loosely arranged as a grid‐like host lattice than molecule **1**. This host lattice formed a channel along the *c*‐axis, and guest molecules existed in that space. The void space, which was 764 and 753 Å^3^ in **1**·Tol and **1**·An crystals, increased to 852 and 837 Å^3^ in **2**·Tol and **2**·An crystals. The percentage of void space in the unit cell volume showed almost the same value of about 32% for both molecule **1** and **2** crystals. While molecule **1** did not form crystals with ClBz, molecule **2** formed a 1:1 formula crystal with ClBz, and its crystal structure was similar to **2**·Tol and **2**·An crystals. The molecular volume of ClBz estimated from DFT calculations (139 Å^3^) occupied 68% of the void space. This value is decreased compared to Tol (156 Å^3^) and An (167 Å^3^) occupying 73 and 80% of the void space, suggesting that ClBz interacts relatively loosely with the host lattice. This is consistent with the magnitude of intermolecular interactions between host and guest estimated by theoretical calculations.

### 2.3 Crystal Transformation From **2**·3(Bz) to **2**·2(Bz)

Molecule **2** yielded **2**·3(Bz) and **2**·2(Bz) crystals depending on the number of included Bz molecules. The former **2**·3(Bz) crystal is unstable at room temperature, and Bz easily desorbs from the crystal, changing to the **2**·2(Bz) crystal. Regarding the percentage of void space in the unit cell, the **2**·3(Bz) crystal has a value of about 50%, which is significantly increased from about 30% seen in other host–guest crystals. By changing from molecule **1** to **2**, the void volume increased significantly from 740 Å^3^ in the **1**·Bz crystal to 921 and 885 Å^3^ in the **2**·3(Bz) and **2**·2(Bz) crystals, and the responsiveness to the guest molecule Bz changed markedly. As a result, only the Bz crystal of molecule **2** formed two different crystal structures.

**Figure 2:**
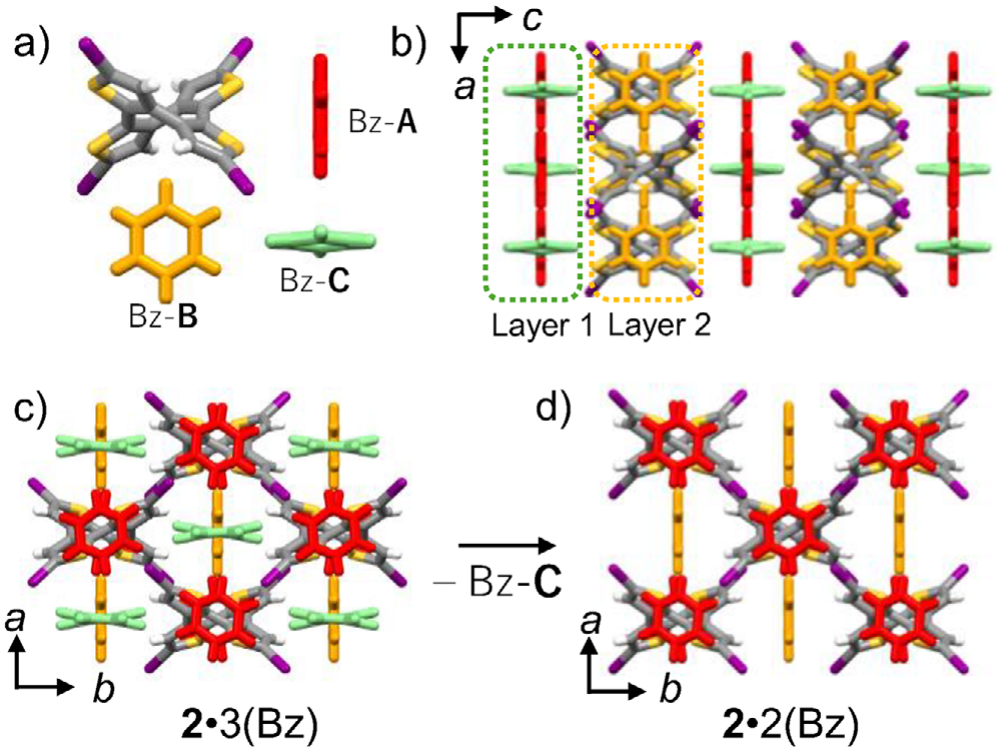
Crystal structure of **2**·3(Bz) and **2**·2(Bz) at 100 K. a) Crystallographically independent molecules of **2**, Bz‐A, Bz‐B, and Bz‐C in single crystal **2**·3(Bz). b) Molecular arrangement of **2**·3(Bz) viewed along the *b*‐axis. Two guest layers of (Bz‐A··· Bz‐C)_∞_ and (Bz‐B)_∞_ along the *a*‐axis. c) Host lattice of **2** via the I···I intermolecular interaction in the *ab* plane and guest Bz arrangement in single crystal **2**·3(Bz). d) Structural transformation from single crystals **2**·3(Bz) to **2**·2(Bz) after the desorption of Bz‐C molecule.

From single crystal X‐ray crystallographic analysis of **2**·3(Bz) and **2**·2(Bz), we examined the intermolecular interactions of Bz molecules with the host lattice. Figure 2a shows crystallographically independent molecule **2**, Bz‐A, Bz‐B, and Bz‐C in the **2**·3(Bz) crystal. The three independent Bz molecules were incorporated into the host lattice with different intermolecular interactions. Figure 2b, c shows the molecular arrangement pattern of the **2**·3(Bz) crystal viewed along the *b*‐axis and *c*‐axis of the unit cell. A 2D layer was formed in the *ab* plane of the unit cell, and pores similar to the 1D channel structure were formed. Bz‐A molecules existed between molecules and no I···I interactions were observed. The guest molecules Bz‐A and Bz‐C formed a 2D layer along the *a*‐axis, perpendicular to each other (Figure 2c). On the other hand, Bz‐B molecules were arranged in a 1D manner along the *a*‐axis. Bz‐B was fixed by C−H···π interactions (*d* = 2.974∼3.148 Å) from the four thienylenes of molecule **2**, and Bz‐A was fixed by S···π interactions (*d* = 3.477∼3.757 Å) from two thienylenes of molecule **2**. Meanwhile, Bz‐C formed a T‐shaped stack structure with Bz‐A and existed with C−H···π interactions (3.110 Å). Therefore, the strength of intermolecular interactions between molecule **2** and Bz is considered to decrease in the order of Bz‐B > Bz‐A > Bz‐C.

**Figure 3:**
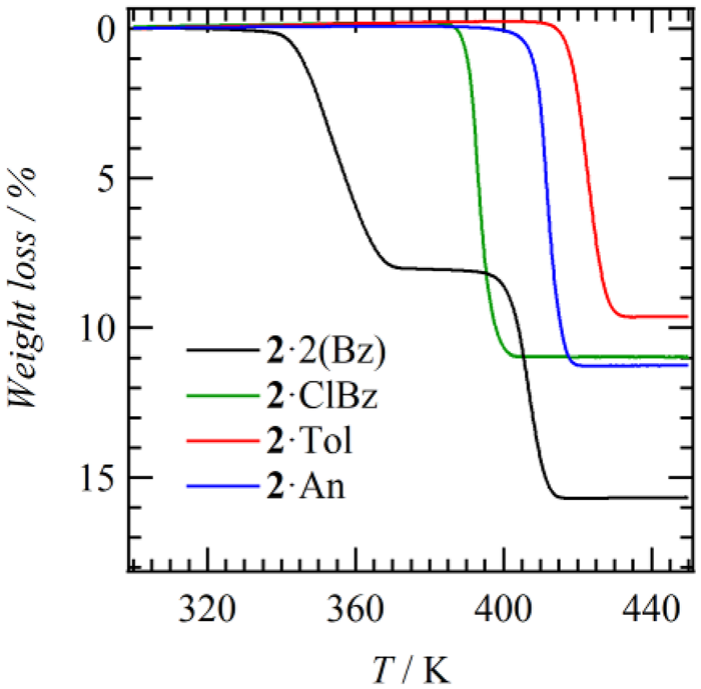
TG charts of **2**·guest crystals.

The conversion from crystal **2**·3(Bz) to **2**·2(Bz) occurred easily with the desorption of one Bz‐C molecule at room temperature. Figure 3 shows the TG charts of **2**·2(Bz), **2**·Tol, **2**·An, and **2**·ClBz crystals consisting of host lattice **2**. For host lattice **1**, **1**·Bz began to lose weight around 380 K and showed a weight loss of 10.5% at 400 K, which showed good agreement with the calculated value of 10.8% for one Bz molecule (Figure S10). On the other hand, the TG chart of **2**·2(Bz) showed clearly distinguishable two‐step weight losses at 340 and 400 K, which were 8.03% and 15.7%, respectively. These showed good agreement with the theoretical calculated values of 7.90 and 15.8% for the stepwise desorption of one Bz molecule each. Since it is considered that guest molecules desorb in order from those with smaller inclusion energy to the host lattice, it is thought that at room temperature, Bz‐C has already desorbed to form **2**·2(Bz), and furthermore, Bz‐A desorbs at 340 K. Also, it was confirmed that Bz‐A desorbs when the sample is left for 1 week (Figure S11). The desorption of Bz‐A corresponds to a change to a channel‐type crystal structure. With further temperature increase, the second step of Bz desorption occurs at 400 K, which was higher than that of **1**·Bz. The desorption onset temperatures of guest molecules in **2**·Tol, **2**·An, and **2**·ClBz formed by molecule **2** were 420, 410, and 390 K, respectively, which are close to the desorption temperature of the second step of Bz in **2**·2(Bz). Therefore, it is considered that **2**·2(Bz) changes to **2**·Bz with a guest channel structure at 400 K, and complete desorption of guest molecules occurs at 420 K.

### 2.4 Guest Sorption Properties and Reversibility

**Figure 4:**
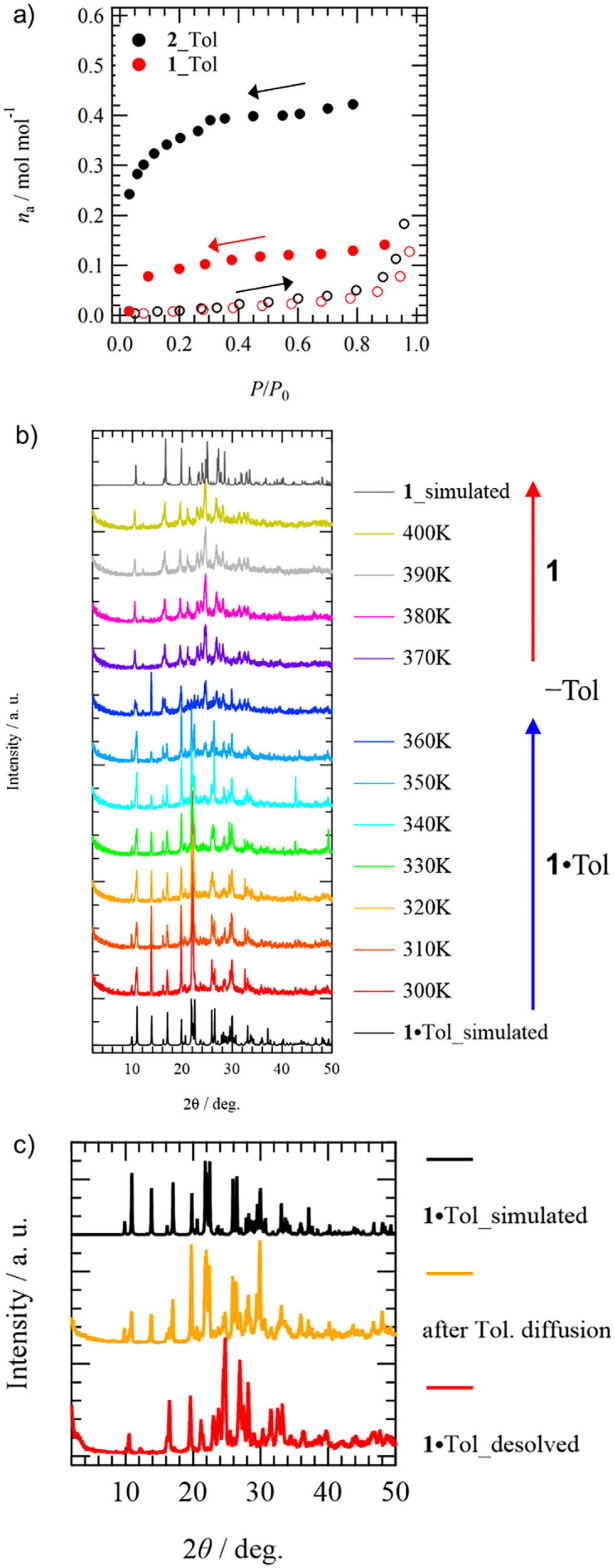
Reversibility of Tol adsorption‐desorption process of host lattice **1**. a) Adsorption‐desorption isotherms of Tol for **1** and **2** at 313 K. b) Variable temperature PXRD patterns of **1**·Tol. Desorption of Tol from **1**·Tol crystals was observed around 370 K. c) structural reversibility of **1**·Tol by Tol diffusion.

In these crystals formed by highly flexible halogen interactions, it is expected that their structure can flexibly change with the presence or absence of guest molecules. Therefore, we examined the reversibility of the host lattice to the adsorption and desorption of guest molecules. From the TG chart, it was shown that crystals **1** and **2** including guest molecules did not show desorption of guest molecules near room temperature and had high thermal stability. Figure 4a shows the adsorption isotherms of **1** and **2** for Tol at 313 K. The adsorption isotherm of Bz at 298 K showed only adsorption of Bz molecules from *n*_a_ = 0.1 to 0.2 mol mol^−1^ (Figure S12), which is consistent with the result that guest molecules stably exist in the channel at room temperature in TG measurements. In the adsorption isotherms of Tol at 313 K, adsorptions of *n*_a_ = 0.15 and 0.4 mol mol^−1^ were confirmed for **1** and **2** with increasing relative pressure *P*/*P*_0_. If measurements at higher temperatures were possible, they might show an adsorption amount of *n*_a_ = 1 mol mol^−1^. Under the same conditions, host lattice **2** adsorbs more Tol molecules than **1**, suggesting that host lattice **2** composed of intermolecular interactions between I atoms has higher flexibility than lattice **1**. Also, for **2**, reversible adsorption of Tol in a 1:1 composition is confirmed in a vial sealed with guest, while for ClBz with almost the same size, complete 1:1 re‐adsorption was not observed (Figure S13). This is because the 1D halogen chain formed by **2** forms a channel structure where chains are nested with weak C − H···π interactions without S···S bonds. Furthermore, for Bz and An, re‐adsorption were not observed like ClBz. The adsorption characteristics of guest molecules are governed depending on the size of guest molecule and the intermolecular interaction energy between host and guest (discussed later).

To examine the retention of crystallinity and reversibility associated with solvent adsorption and desorption, variable‐temperature powder X‐ray diffraction (PXRD) measurements were conducted. Figure 4b shows the variable‐temperature PXRD patterns of **1**·Tol crystal from 300 to 400 K. TG measurements confirmed that desorption of Tol molecules begins around 340 K. The PXRD pattern at 300 K showed good agreement with the simulation XRD pattern calculated from single crystal X‐ray crystallographic analysis at 100 K. With increasing temperature, changes in the Bragg reflections observed in PXRD occurred around 350 K, confirming changes in the crystal structure. This PXRD pattern matched the simulation pattern calculated from single crystal X‐ray crystallographic analysis of guest molecule **1** only. Since sharp Bragg reflections were observed even at 400 K, desorption of guest molecules occurred while maintaining high crystallinity, changing to a crystal lattice consisting only of host **1**.

The host lattice **1** from which Tol has desorbed did not return to a 1:1 formula by complete adsorption of Tol molecules, as shown by the adsorption isotherms at *T* = 298 and 313 K. However, when molecule **1** was exposed to Tol molecules at 353 K (80 °C) in a sealed vial, it was confirmed to return to the 1:1 formula PXRD pattern (Figure 4c). Therefore, host lattice **1** was capable of reversible adsorption and desorption of Tol, the guest molecule, while maintaining high crystallinity. Similar results were observed for the adsorption of Tol to host lattice **2** (Figure S13). However, host lattices **1** and **2** did not show reversible adsorption and desorption processes for guests other than Tol, such as Bz, An, and ClBz, showing selective reversible adsorption and desorption characteristics only for Tol. Although crystal lattice **2** is considered to be more flexible than crystal lattice **1**, it could not reversibly return to the original **2**·ClBz for ClBz, which is almost the same size as Tol (Figure S13). This difference in stability of Tol and ClBz to the host lattice is considered to be governed by host‐guest intermolecular interactions (discussed later).

**Figure 5:**
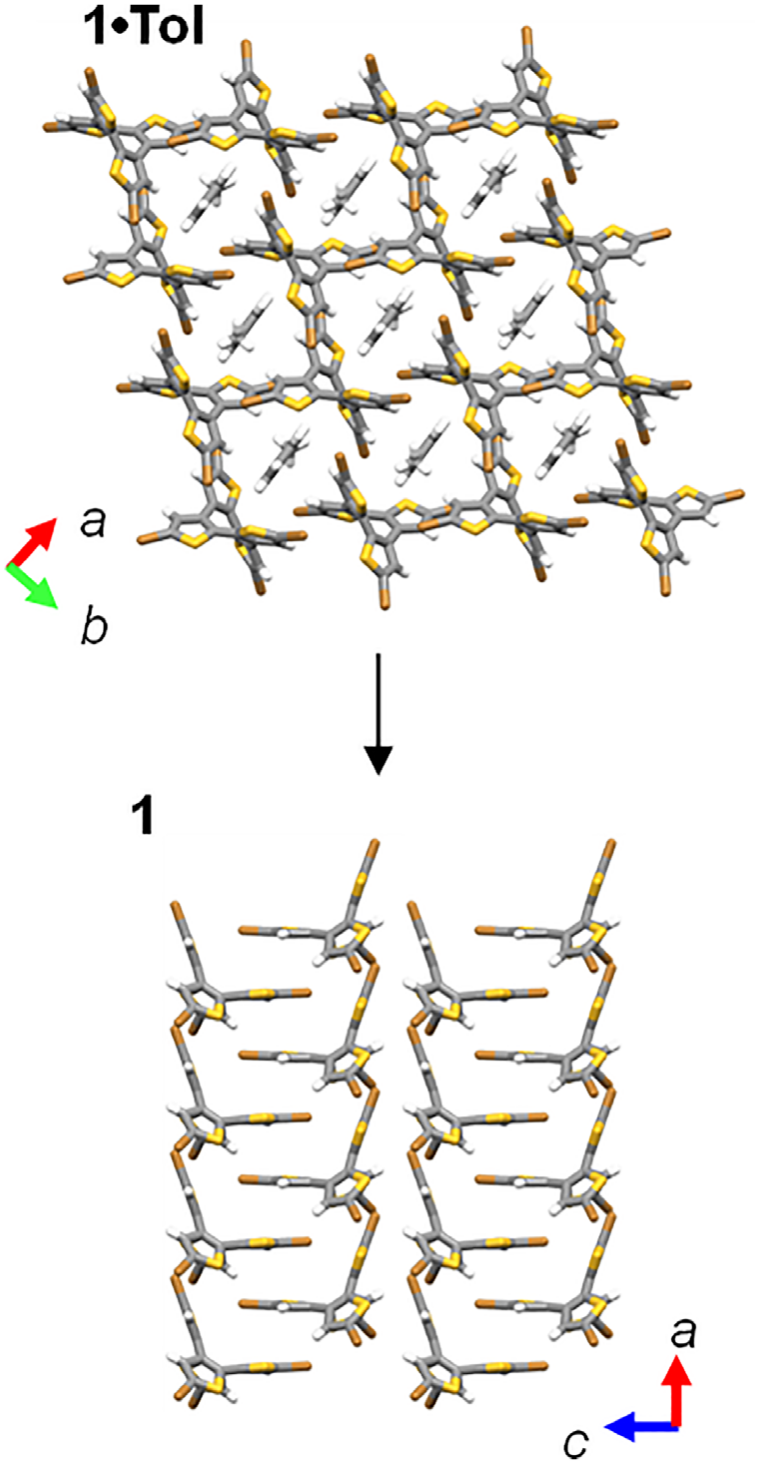
Changes in the host lattice with the desorption of Tol from the **1**·Tol crystal. The left and right figures show the results of single crystal X‐ray crystallographic analysis of **1**·Tol and **1**, respectively.

In host lattices **1** and **2**, guest molecules are introduced into channels formed by the grid arrangement of 1D halogen chains, and reversible guest molecule adsorption and desorption were observed in host–guest crystals containing Tol molecules. Having successfully performed single crystal X‐ray structure analysis of host crystals **1** and **2** without guest molecules, we discuss the reversible structural changes associated with Tol molecule adsorption and desorption. Figure 5a, b shows the crystal structures viewed along the *c*‐ and *b*‐axis of **1**·Tol and **1** (Figures S16, S17). In **1**·Tol, Tol molecules were introduced into the channel within the grid arrangement of 1D chains formed by molecule **1**. In crystal **1**, with the desorption of Tol, the structure changed from the **1**·Tol crystal where Br···Br interactions were dominant to a structure arranged by multipoint Br···S and B···π intermolecular interactions (Figure 5). In crystal **1**, molecule **1** had multipoint interactions with Br groups adjacent to thiophene rings and COT rings in the *ab* plane. As a result, it enabled reversible adsorption and desorption of guest molecules while maintaining good crystallinity. It is considered that similar reversible adsorption and desorption occur for **2**·Tol as well.

### 2.5 Dynamic Behavior

Dielectric constant sensitively reflects the dynamics of dipole moments in crystals. Therefore, in host–guest crystals, dielectric response is observed when the dynamics of guest molecules with dipole moments are thermally excited with increasing temperature. On the other hand, since all crystals have inversion symmetry, even if dipole moment dynamics exist in the crystal, the ground state is antiferroelectric, and its dielectric response is predicted to be small. To evaluate molecular dynamics in host–guest crystals, dielectric constant measurements were performed. Table 2 summarizes the activation energies determined from the temperature (*T*) and frequency (*f*) dependence of the 1D channel occupancy area and dielectric constant (Figures S18–S23).

**Table 2:** Dielectric Properties and Host–Guest Interaction of **1**·Guest and **2**·Guest crystals.

Host·guest	Channel area [Å^2^]	*E*_a1_, eV^[^ ^a^ ^]^	*E*_a2_, eV^[^ ^b^ ^]^	*ΔE*, kcal mol^−1[^ ^c^ ^]^
1·Bz	31	0.73	−	14.5
1·Tol	31	0.71	−	16.3
1·An	31	0.64	−	16.7
2·3(Bz)^[^ ^d^ ^]^	−	−	−	46.5
2·Tol	34	0.74	0.29	16.2
2·An	33	0.74	−	17.4
2·ClBz	33	0.67^[^ ^e^ ^]^	0.18	15.5

^[a]^ Activation energy determined from dielectric relaxation in the low temperature region *T* < 250 K.

^[b]^ Activation energy determined from dielectric relaxation in the high temperature region *T* > 250 K.

^[c]^ Stabilization energy calculated by DFT. Δ*E* = −*E*_HG_ / (number of host molecules) was estimated by DFT calculations using B3LYP/3–21G* as the basis function. Here, *E*_HG_ is the interaction energy of the host–guest structure calculated using the counterpoise method.

^[d]^ Unmeasurable because the crystal is unstable at room temperature.

^[e]^ Measurement using a single crystal.

**Figure 6:**
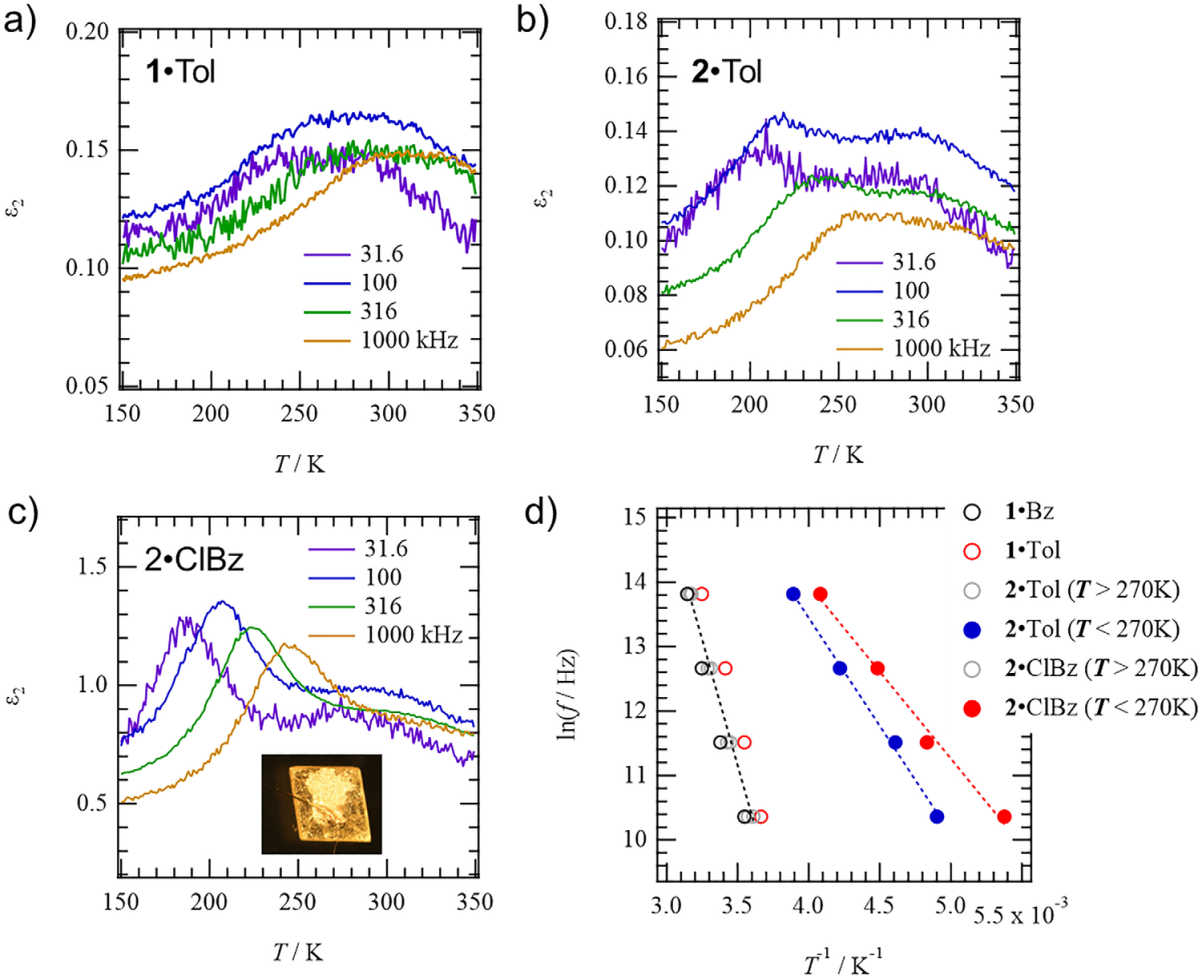
Dielectric response of host–guest crystals. *T*‐ and *f*‐dependent imaginary part dielectric constants (*ε*_2_) of compressed pellets a) **1**·Tol, b) **2**·Tol, and c) single‐crystal **2**·ClBz. d) ln *f*_p_ – *T* ^−1^ plots of **1**·Bz, **1**·Tol, **2**·Tol, and **2**·ClBz.

Figure 6a, b, c shows the *T*‐ and *f*‐dependence of the imaginary part of the dielectric constant (*ε*_2_) for crystals **1**·Tol, **2**·Tol, and **2**·ClBz. The dielectric constants of crystals **1**·Tol and **2**·Tol were measured using compressed pellets, while the dielectric constant of **2**·ClBz was measured along the *a*‐axis of single crystal, which is the channel direction. In all samples, dielectric constant peaks dependent on *T* and *f* were observed at a specific temperature *T*_p_ at a specific frequency *f*_p_, indicating the existence of a dipole moment relaxation process in the crystal. For **1**·Tol, the peak of *ε*_2_ seen at 273 K at 31.6 kHz shifted to 308 K when the measurement *f* was increased to 1000 kHz. Since only one type of peak was observed in the **1**·Tol crystal, it indicates the existence of a typical single Debye‐type relaxation process, and its ln *f*_p_ − *T*_p_^−1^ plots were well reproducible by the Arrhenius equation, with an activation energy *E*_a1_ = 0.71 eV (Figure 6d). Similarly, single Debye‐type relaxation processes were observed for **1**·Bz and **1**·An crystals using compressed pellets, yielding values of *E*_a1_ = 0.73 and 0.64 eV, respectively. Crystals **1**·Tol, **1**·Bz, and **1**·An all showed single Debye‐type relaxation processes with similar *E*_a1_ values. Notably, since Bz molecules do not have a dipole moment, the observed dielectric response is considered to be a result attributable to the host lattice. Therefore, in host–guest crystals formed by molecule **1**, guest molecules incorporated into the host lattice are stabilized by effective intermolecular interactions, indicating that there is no dynamics of dipole moments due to thermal excitation.

**Figure 7:**
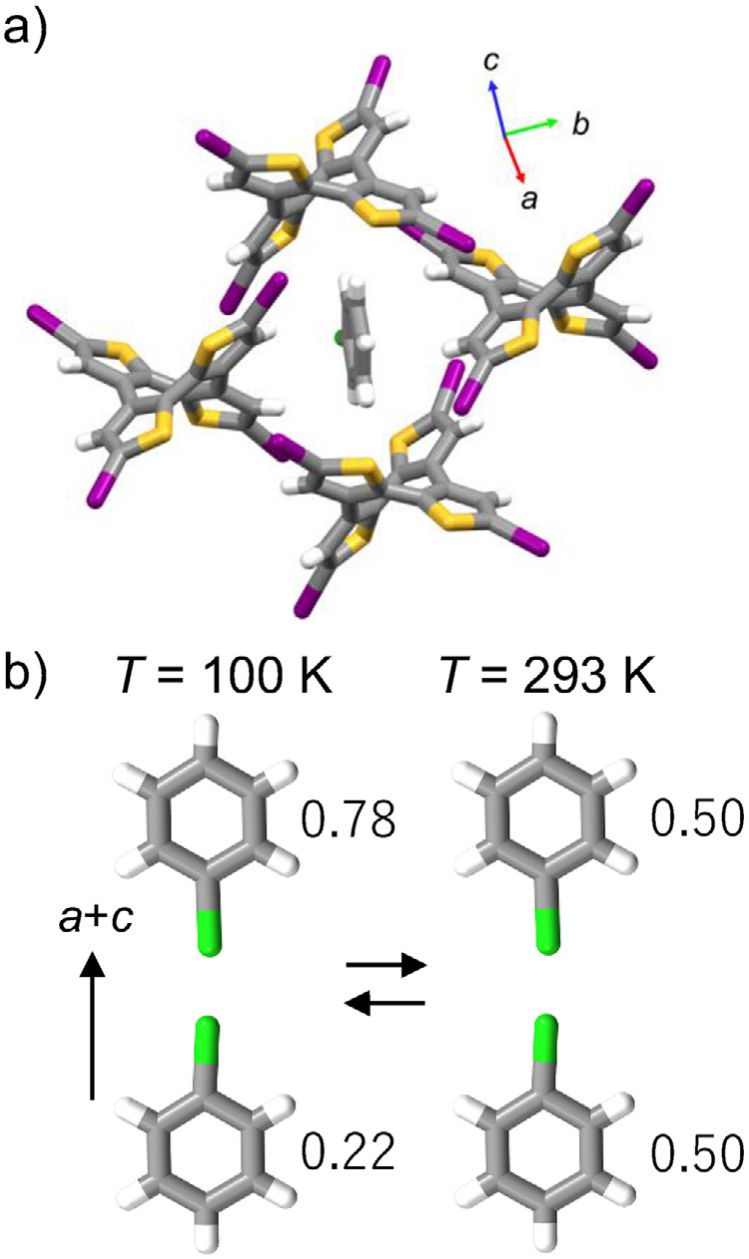
Thermally activated reversible guest dynamics in host lattice of **2**. *T*‐dependent change in the occupied states of ClBz at 100 K (upper) and 293 K (lower).

On the other hand, guest inclusion crystals formed by molecule **2** showed different behavior from molecule **1**. Crystals **2**·Tol and **2**·ClBz exhibited two different relaxation processes in the *T*‐ and *f*‐dependence of *ε*_2_, one in the low *T* region (*T* < 250 K) and one in the high *T* region (*T* > 250 K) (Figure 6, 7). In the compressed pellet sample of **2**·Tol, maxima of *ε*_2_ appeared at 204 and 280 K in the measurement at *f* = 31.6 kHz, and both shifted to higher temperatures of 257 and 316 K at *f* = 1000 kHz. Using *f*_p_ and *T*_p_ from the low‐ and high‐*T* regions to make ln *f*_p_ – *T*_p_^−1^ plots, good linear relationships were obtained, and the activation energies derived from them were *E*_a2_ = 0.29 eV (high‐*T* region) and *E*_a1_ = 0.74 eV (low‐*T* region). The *T*‐range and *E*_a2_ of the relaxation process observed in the high‐*T* region were similar to the appearance *T*‐range and *E*_a_ value of the relaxation observed in **1**·Tol. Similarly, in the **2**·ClBz single crystal, from ln *f*_p_–*T*_p_^−1^ plots, two types of relaxation processes were observed in one with *E*_a2_ = 0.18 eV in the low‐*T* region and the other with *E*_a1_ = 0.67 eV in the high‐*T* region. The latter is considered to be similar to the relaxation processes seen in the high‐*T* region of **2**·Tol and in **1**·Tol. Therefore, the dielectric relaxation observed in the low‐*T* region in *ε*_2_ of **2**·Tol pellet and **2**·ClBz single crystal is considered to reflect the dynamics of guest molecules with dipole moments. In the **2**·Bz crystal with Bz, which has no dipole moment, as a guest, despite having the same host lattice as molecule **2**, no dielectric relaxation was observed in the low‐*T* region, and dielectric relaxation in the high‐*T* region was observed with *E*_a1_ = 0.37 eV. From these results, it is considered that the dielectric relaxation with *E*_a1_ = 0.64 − 0.74 eV commonly observed in the high‐*T* region in **1**·(Bz, Tol, An) and **2**·(Bz, Tol, An, ClBz) reflects the asymmetric thermal fluctuations of the host lattice. On the other hand, the low‐*T* relaxation process *E*_a2_ observed in **2**·(Tol, ClBz) is a result reflecting the dynamics of polar guest molecules. In the **2**·An crystal with a larger guest size, only the relaxation process with *E*_a1_ = 0.74 eV in the high‐*T* region was observed, and *E*_a2_ reflecting the dynamics of guest molecules in the low‐*T* region was not confirmed (Figure S26). This is a result of the larger sized An not being able to move freely like Tol or ClBz. The dynamics of ClBz in host lattice **2** (*E*_a2_ = 0.18 eV) had a lower *E*_a_ value than the dynamics of Tol (*E*_a2_ = 0.29 eV). The molecular volumes of Tol and ClBz are 156 and 139 Å^3^, respectively, and since ClBz is smaller, it is thought to lower the activation barrier. However, it is considered that there are other factors that reduce *E*_a_ by about 60% compared to the value for Tol (discussed later). Furthermore, despite host lattice **1** forming a **1**·An crystal with the larger sized An (167 Å^3^), the fact that ClBz, which is smaller in size than An, did not form a **1**·ClBz crystal is related to the intermolecular interactions working between ClBz and the host lattice.

### 2.6 Guest Dynamics in Host Lattices

From the crystal structure at 100 K, the stabilization energy Δ*E* = −*E*_HG_ / (number of host molecules) due to guest inclusion per host molecule was estimated by DFT calculations using B3LYP/3–21G* as the basis function. Here, *E*_HG_ is the interaction energy of the host–guest crystal calculated using the counterpoise method, and GD3BJ was used for the dispersion force correction term. Also, the molecular structures used in the calculations used the atomic coordinates obtained from single crystal X‐ray structure analysis at 100 K.

On the other hand, in the host lattice of molecule **2** formed by I···I interactions, the Δ*E* values for **2**·3(Bz), **2**·Tol, **2**·An, and **2**·ClBz were 13.8 (value converted per guest molecule), 16.2, 17.4, and 15.5 kcal mol^−1^ (calculated value for the major component), respectively. For Tol and An with electron‐donating groups, the stabilization energy for the host lattice containing thiophene rings increased, whereas for ClBz with an electron‐withdrawing group, the Δ*E* value was small. In TG measurements, the temperatures at which weight loss occurred for **2**·Tol, **2**·An, and **2**·ClBz were 420, 410, and 390 K, and the stability of guest molecules in host lattice **2** decreased in the order of Tol > An > ClBz. In particular, the result of a significant decrease in **2**·ClBz is consistent with the decrease in Δ*E* by DFT calculations. Also, despite the molecular volumes of Tol and ClBz being approximately the same, the fact that their stabilization energies for host lattice **2** differ greatly from each other was also consistent with the magnitude relationship of activation energies for dielectric relaxation. The dielectric dynamics of guest molecules are governed not only by size compatibility but also by electronic interactions with the host lattice.

For **1**·Bz, **1**·Tol, and **1**·An crystals, stabilization energy values of Δ*E* = 14.5, 16.3, and 16.7 kcal mol^−1^ were obtained, respectively, showing that Tol and An are more stably included than Bz. Since the molecular volumes of Bz and Tol are 125 and 156 Å^3^, respectively, the larger sized Tol molecule could exist stably by fitting size into the host lattice formed by **1**. **1**‐guest crystals **1**·(Bz, Tol, and An) showed the first step of weight loss around 380 K in TG measurements, and showed weight losses of 11, 13, and 15%, respectively, at *T* > 400 K. These values are consistent with the calculated values corresponding to **1** mole of each guest molecule. The onset temperature of Tol desorption in TG measurement (390 K) is 10 K higher than that of Bz (380 K), and Tol molecules existed stably in the channel in the host lattice through dipole–dipole interactions. Therefore, in the *T*‐ and *f*‐dependence of the dielectric constant of **1**·Tol, the dynamics of Tol, a polar guest molecule, were not observed, and only the dynamics of the host lattice were observed as a result.

As a result of evaluating the dynamics of the polar guest molecule ClBz in the host lattice **2**·ClBz crystal formed by I···I interactions using dielectric constant, it interacts more weakly with the host lattice than the **2**·Tol crystal, and there is a larger dynamics. Next, we attempted to evaluate the dynamics of guest molecules crystallographically. The **2**·Tol crystal containing Tol as a guest has a center of symmetry in space group *I*2/*a* in X‐ray crystallographic analysis at 100 K, and there is no dipole moment in the crystal as a whole. Orientational disorder of the methyl group was observed in the Tol molecules in the channel, with an occupancy ratio of 0.5:0.5. Therefore, Tol molecules are arranged alternately upward and downward in the channel to cancel out their dipole moments. Crystallographically, it is difficult to discuss the dynamics of polar Tol molecules due to temperature changes from changes in the crystal structure.

On the other hand, for ClBz with weak intermolecular interactions with host lattice **2**, orientational disorder of the Cl atom was observed in the X‐ray crystallographic analysis of the **2**·ClBz crystal at 100 K, and interestingly, its occupancy ratio was 0.22:0.78 (Figure 7). The space group at 100 K is *P*2_1_/*c* with a center of symmetry, and although there is a bias in the occupancy ratio of the orientational disorder of ClBz, it is a nonpolar crystal where dipole moments cancel each other out as a whole crystal. When the single crystal analyzed by X‐ray structure analysis at 100 K was heated to 293 K and similarly analyzed, the space group changed from *P*2_1_/*c* to *I*2/*a*, and the occupancy ratio of the orientational disorder of the Cl atom changed to 0.5:0.5. Furthermore, when the same single crystal was cooled again to 100 K and crystal structure analysis was performed, the space group returned to *P*2_1_/*c*, and the occupancy ratio of ClBz also returned to the original state of 0.22:0.78. Therefore, this change in crystal structure seen at 100 and 293 K occurs reversibly with temperature. On the other hand, in DSC, no peak indicating a clear phase transition was observed in the measurable temperature range (133∼300 K) (Figure S27), while the dielectric relaxation due to the dynamics of polar guest molecules appears at 180∼250 K. From these results, ClBz in host lattice **2** undergoes thermally excited twofold flip‐flop rotational motion, and it is in a free rotation state overcoming the rotational barrier near room temperature. However, with decreasing temperature, the dynamics gradually freezes, and the occupancy ratio becomes 0.22:0.78 at 100 K, and it is predicted that the occupancy ratio will become 0:1 at a certain temperature as the temperature is further lowered. The dynamics of ClBz completely freeze at temperatures below 100 K. With the freezing of this ClBz dynamics, it is considered that the symmetry of the crystal decreased as the arrangement pattern of host lattice **2** changed at low‐*T*. By using ClBz guest with weak intermolecular interactions with host lattice **2**, it is considered that the dynamics of guest molecules were thermally excited, causing dielectric response and affecting crystal symmetry.

## 3 Conclusion

Using X4‐tetra[2,3]thienylene with four halogen atoms (X = Br or I) introduced at the terminals of the tetra[2,3]thienylene framework, we investigated the formation of various guest inclusion crystals through halogen···halogen interactions and molecular dynamics. Br4–tetra[2,3]thienylene (**1**) and I4‐tetra[2,3]thienylene (**2**) formed host–guest crystals with aromatic molecules (Bz, Tol, An, and ClBz) in various composition ratios. Molecule **1** formed crystals with 1:1 formula with Bz, Tol, and An, while did not form host–guest crystals with ClBz. On the other hand, molecule **2** formed crystals with 1:2 and 1:3 formula with Bz, and host–guest crystals with 1:1 formula with Tol, An, and ClBz. X‐ray crystallographic analysis revealed that 1D chains formed by intermolecular X···X interactions arranged in a grid pattern to form a 1D channel structure. Also, by changing Br atoms to I atoms, the size of the 1D channel formed by the host lattice increased, and by making the host lattice structurally more flexible, it became possible to introduce various guest molecules. The host lattice stably adsorbed guest molecules at room temperature and maintained crystallinity after desorption of guest molecules by heating, while selective and reversible adsorption and desorption were possible only when Tol was used as the guest molecule. From dielectric constant measurements, no dynamics of guest molecules were observed in the host lattice formed by molecule **1**, and only the dynamics of the host lattice were thermally excited in the high‐*T* region. On the other hand, in the host lattice formed by molecule **2**, dielectric relaxation due to the dynamics of polar guest molecules (Tol and ClBz) was observed in the low‐*T* region. In particular, in crystals containing ClBz, the occupancy ratio of orientational disorder at 100 K changed depending on temperature, and flip‐flop rotational motion of ClBz molecules occurred. The interaction energy of ClBz with the host lattice calculated by DFT calculations was small and was consistent with the sequence of desorption temperatures of guest molecules in TG measurements (Tol > An > ClBz) and the sequence of activation energy of dielectric relaxation. This study revealed the design of flexible porous materials using halogen···halogen interactions and dielectric response due to the dynamics of polar guest molecules. It is of great significance in that it clarified the differences in interactions due to the types of halogen atoms and their effects on the structure and properties of host–guest crystals. In the future, based on this knowledge, the development of XOFs with excellent external field responsiveness is expected.

## Supporting Information

Experimental Section, crystal parameters, optimized molecular structures, crystal structures, TG charts, sorption isotherm of Bz at 298 K, structural change of **2** after Tol and ClBz readsorption, single crystal simulation, VT‐PXRD, crystal structures of **1** and **2**, dielectric constants, Arrhenius plots, DSC chart of **2**·ClBz, and two‐level model analysis of occupancy factor of ClBz in **2**·ClBz.

## Conflict of Interest

The authors declare no conflict of interest.

## Supporting information



Supporting Information

Supporting Information

## Data Availability

The data that support the findings of this study are available from the corresponding author upon reasonable request.
