# Supplementary material for: Porous Organic Frameworks Utilizing Halogen···Halogen Interactions of X4–tetra[2,3]Thienylene (X = Br, I): Guest Dynamics and Dielectric Response
Source: Chemistry. 2025 Nov 10;31(71):e02872. doi: 10.1002/chem.202502872 (PMC12734654; doi:10.1002/chem.202502872)
Supplement: Supplementary file 1 — Supporting Information [file CHEM-31-e02872-s002.pdf]

Supporting Information

Porous Organic Frameworks Utilizing Halogen···Halogen Interactions: Guest Dynamics and Dielectric Response of X4-TTT (X = Br, I)

Genki Saito,[a] Takashi Takeda,[a, b, c]* Shun Dekura,[a, b] Junpei Moriguchi,[d] Tetsu Sato,[a, b] Ryo Tsunashima, [d] and Tomoyuki Akutagawa [a, b]*

a Graduate School of Engineering, Tohoku University, Sendai 980-8579, Japan, b Institute of Multidisciplinary Research for Advanced Materials (IMRAM), Tohoku University, 2-1-1 Katahira, Aoba-ku, Sendai 980-8577, Japan, c Faculty of Science, Shinshu University, 3-1-1 Asahi, Matsumoto 390-8621, Japan, and d Graduate School of Sciences and Technology for Innovation, Yamaguchi University, Yoshida 1677-1, Yamaguchi, 753-8512, Japan.

E-mail takashi@shinshu-u.ac.jp and akutagawa@tohoku.ac.jp

Contents

1. Experimental Section

2. Crystal parameters (Table S1).

3. Optimized molecular structure of **1** and **2** (Figure S1).

4. Crystal structures and ORTEP diagram of **1**•Tol (Figure S2 – S4).

5. Thermal parameter change of Tol in **1**•Tol (Figure S5).

6. Crystal structures and ORTEP diagram of **1**•Bz and **1**•An (Figures S4 – S9).

7. Crystal structures and ORTEP diagram of **2**•Tol, **2**•An, and **2**•ClBz (Figures S10-18).

8. Crystal structures of **2**•3(Bz) and **2**•2(Bz) and host-guest interaction of **2**•3(Bz) (Figure S19 -20).

9. Summary of X-X distance and C-X···X angle of type A crystals (Table S2)

10. The calculated total energies (per formula) of the optimized **2**·3Bz (Table S3)

11. TG charts of **1**•Bz, **1**•Tol, **1**•An, and **2**•2(Bz) (Figures S21 - 22).

12. Sorption isotherm of Bz at 298 K (Figure S23).

13. Structural change of **2** after Tol and ClBz re-adsorption (Figure S24).

14. Single crystal simulation and powder pattern of **1**•Tol and **2**•Tol (Figure S25).

15. VT-PXRD of **1**•Bz, **1**•Tol, and **2**•ClBz (Figures S26-28).

16. Crystal structures of **1** and **2** (Figures S29 - 30).

17. Dielectric constants (Figures S31-36)

18. Arhenius plots of dielectric measurements (Figure S37).

19. DSC chart of **2**•ClBz (Figure S38).

20. Two-level model of occupancy factor of ClBz in **2**•ClBz (Figure S39).

21. Reference

Experimental Section

General. Commercially available reagents and solvents were used without purification. ^{1}H NMR measurements were performed on a Bruker Avance III 400 NMR spectrometer. Tetramethylsilane (δ = 0.00 ppm) was used as a reference substance for chemical shifts (δ) in ^{1}H NMR (400 MHz). Medium pressure columns from Yamazen Corporation, EPCLC AI-580 and Yamazen columns (Hi-Flash column silica gel, Universal Column silica gel) were used. Thermogravimetric analysis (TG-DTA) measurements were performed using Thermo Plus EVO2 TG8121 from Rigaku Corporation, under nitrogen atmosphere (flow rate 500 mL/min) with a heating rate of 10 K/min. Al pans were used as measurement containers, and Al2O3 was used as a reference. For differential scanning calorimetry (DSC) measurements, METTLER TOLEDO STARe System DSC3-TS was used. Measurements were performed at a scanning rate of 5 K/min under N2 flow atmosphere (30 mL/min). An empty Al pan was used for the reference cell. The temperature-frequency dependence of the dielectric constant was measured by AC impedance method using a temperature variable stage. LINKAM LTS-350 was used for the temperature variable stage, and Keysight Technologies E4990A was used for the impedance analyzer. Powder samples were ground in an agate mortar and then formed into pressure pellets (3 mm φ). Ag paste was applied to both sides of the pellet or single crystal samples, and they were connected to the measuring device using gold wire (25 μm φ). Powder X-ray diffraction (PXRD) was performed using a RAPID-II diffractometer (Rigaku Corporation) or SmartLab 3kW (Rigaku Corporation). Both used Cu Kα (λ = 1.54187 Å) monochromatized with a graphite monochromator as the X-ray source, and samples were ground in an agate mortar. For the RAPID-II diffractometer (Rigaku Corporation), powder samples were introduced into a 0.2 mm φ capillary tube for X-ray analysis, and measurements were performed using a nitrogen blowing device for temperature control. For SmartLab 3kW (Rigaku Corporation), a parallel beam optical system was used, powder samples were introduced into a depression on a glass plate for measurement, and measurements were performed under N_2 flow (100 mL min^{-1}) using a custom temperature control stage.

Preparations of 1 and 2. Compound **1** was obtained in 87% yield by treating tetratrimethylsilyltetra[2,3]thienylene with *N*-bromosuccinimide according to existing methods. The ^{1}H NMR spectrum of the obtained compound agreed well with previously reported data. For compound **2**, with reference to the synthesis method of compound **1**, tetratrimethylsilyltetra[2,3]thienylene (511 mg, 828 μmol), chloroform 38 mL, and acetic acid 38 mL were added to a 100 mL flask and stirred, then *N*-iodosuccinimide (1.0996 g, 4.89 mmol, 5.9 eq) was added to the reaction solution and stirred

overnight at room temperature. After overnight stirring, the solution became a suspension. The suspension was vacuum filtered, and the filtered solid was washed with methanol to obtain a white solid, 470 mg (68% yield).

Compound **1**. ^{1}H NMR (400 MHz, $CDCl_3$): δ 6.90 (s, 4H) Elemental Analysis. Calc. for C23H12S4Br4 [(1)•toluene]: C: 37.45, H: 1.57, N:0.00, Found: C: 37.52, H:1.64, N: 0.00.

Compound **2**. ^{1}H NMR (400 MHz, CDCl3): δ 7.06 (s, 4H), Elemental Analysis. Calc. for C23H12S4I4 [**2**•toluene]: C: 29.89, H: 1.31, N:0.00, Found: C: 29.81, H:1.60, N: 0.00.

Single crystal growth of 1, 2, 1•n(Guest) and 2•n(Guest). Molecules **1** and **2** were dissolved in each aromatic solvent by heating, and the target single crystals were prepared by vapor diffusion of methanol at room temperature. Also, molecules **1** and **2** were dissolved in THF, and single crystals without solvent inclusion were prepared by vapor diffusion of methanol at room temperature.

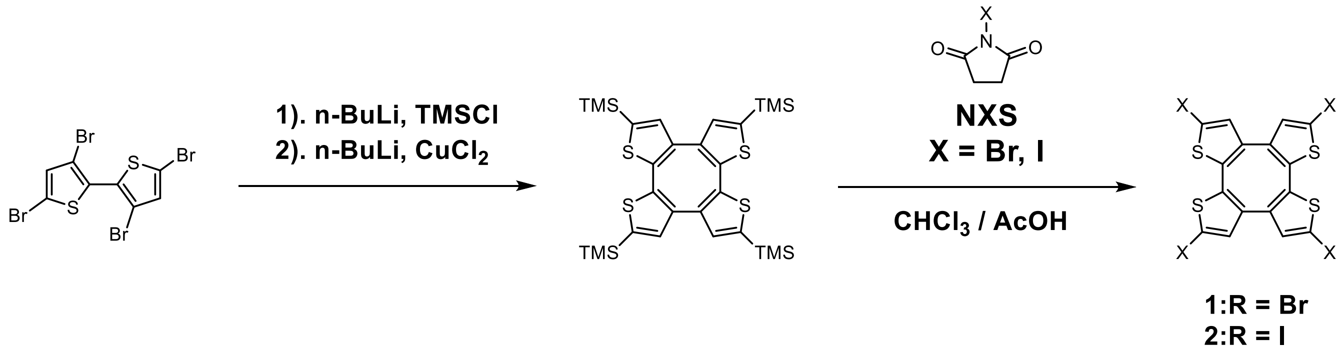

Scheme S1. Synthetic route of compounds **1** and **2** [1,2].

Crystal structural determination. Crystallographic data were collected using a Rigaku RAPID-II diffractometer equipped with a rotating anode fitted with a multilayer confocal optic and using Cu Kα (λ = 1.54187 Å) radiation from a graphite monochromator. Structural refinements were performed using the full-matrix least-squares method on F^2. Calculations were performed using Crystal Structure software package Olex2. All the parameters, except for those of the hydrogen atoms, were refined using anisotropic temperature factors. Table S1 summarizes crystal data and parameters of six single crystals.

Table S1. **Crystal Data, Data Collection, and Reduction Parameters of 1, 2, 1•n(guest) and 2•n(guest).**

**Crystal**	**1**	**1**•Bz	**1**•Tol	**1**•Tol	**1**•An
Chemical formula	$C_{16}H_4S_4Br_4$	$C_{22}H_{10}S_4Br_4$	$C_{23}H_{12}S_4Br_4$	$C_{23}H_{12}S_4Br_4$	$C_{23}H_{12}OS_4Br_4$
Formula weight	644.07	722.18	736.21	736.21	752.21
T, K	100	100	100	293	100
Space group	*Pnma* (#62)	*I*2/*a* (#15)	*I*2/*a* (#15)	*I*2/*a* (#15)	*I*2/*a* (#15)
a, Å	7.8916(2)	10.6989(4)	10.5686(3)	10.8442(4)	10.7951(2)
b, Å	16.5739(3)	12.7048(5)	12.7008(4)	12.7904(5)	12.6150(2)
c, Å	14.4346(3)	18.2406(16)	18.5946(15)	18.5673(16)	18.4437(14)
α, deg	–	–	–	–	–
β, deg	–	105.736(7)	104.618(7)	105.255(7)	106.109(6)
γ, deg	–	–	–	–	–
V, Å^3	1887.97(8)	2386.5(3)	2415.2(2)	2484.6(3)	2413.0(2)
Z	4	4	4	4	4
D_{calc}, g·cm^{-3}	2.266	2.010	2.025	1.968	2.071
μ, mm^{-1}	14.517	11.576	11.454	11.134	11.514
Reflections measured	20371	13347	13215	13984	13663
Independent reflections	1794	2176	2211	2273	2208
Reflections used	1794	2176	2211	2273	2208
R_{int}	0.0604	0.0642	0.0447	0.0513	0.0507
R_1 [a]	0.0441	0.0361	0.0271	0.0391	0.0311
R_{all}	0.0491	0.0381	0.0302	0.0431	0.0338
$R_w(F_2)$ [a]	0.0950	0.0979	0.0704	0.0953	0.0809
GOF	1.135	1.129	1.155	1.111	1.148
CCDC	2482318	2482308	2482320	2482311	2482315

[a] $R_1 = \Sigma||F_o| - |F_c|| / \Sigma |F_o|$ and $R_w = (\Sigma\omega(|F_o| - |F_c|)2 / \Sigma\omega F_o^2)^{1/2}$.

continued

**Crystal**	**2**	**2**•2(Bz)	**2**•3(Bz)	**2**•Tol	**2**•Tol
Chemical formula	$C_{16}H_4S_4I_4$	$C_{28}H_{16}S_4I_4$	$C_{28}H_{16}S_4I_4$	$C_{23}H_{12}S_4I_4$	$C_{23}H_{12}S_4I_4$
Formula weight	832.03	988.25	1066.35	924.17	924.16
T, K	100	100	100	100	293
Space group	*Pnma* (#62)	*Ibam* (#72)	*Ibam* (#72)	*I*2/*a* (#15)	*I*2/*a* (#15)
a, Å	8.1735(6)	10.3980(8)	10.3141(5)	10.9151(3)	11.1025(5)
b, Å	17.3132(13)	13.1641(10)	13.1039(6)	13.0111(4)	13.0763(5)
c, Å	14.9519(9)	25.809(2)	25.5756(12)	19.1733(14)	19.1696(16)
α, deg	–	–	–	–	–
β, deg	–	–	–	105.147(6)	105.547(7)
γ, deg	–	–	–	–	–
V, Å^3	2115.8(3)	3532.7(5)	3456.7(3)	2628.3(2)	2681.2(3)
Z	4	4	4	4	4
D_{calc}, g·cm^{-3}	2.612	1.858	2.049	2.335	2.289
μ, mm^{-1}	49.932	30.029	30.752	40.292	39.498
Reflections measured	21658	18479	18070	14347	14072
Independent reflections	2001	1661	1632	2412	2447
Reflections used	2001	1632	1632	2412	2447
R_{int}	0.1829	0.1278	0.0962	0.1344	0.1314
R_1 [a]	0.0698	0.0582	0.0660	0.0626	0.0538
R_{all}	0.1321	0.0753	0.0756	0.0686	0.0932
$R_w(F_2)$ [a]	0.1836	0.1642	0.1667	0.1609	0.1161
GOF	0.972	1.111	1.171	1.055	0.963

CCDC	2482319	2482312	2482309	2482317	2482316

[a] $R_1 = \Sigma||F_o| - |F_c|| / \Sigma |F_o|$ and $R_w = (\Sigma\omega(|F_o| - |F_c|)^2 / \Sigma\omega F_o^2)^{1/2}$.

continued

**Crystal**	**2**•An	**2**•ClBz	**2**•ClBz
Chemical formula	$C_{23}H_{12}OS_4I_4$	$C_{22}H_9S_4ClI_4$	$C_{22}H_9S_4ClI_4$
Formula weight	940.17	944.58	944.58
T, K	100	100	293
Space group	*I*2/*a* (#15)	*P*2$_1$/*c* (#14)	*I*2/*a* (#15)
a, Å	10.9873(2)	19.2156(8)	11.0401(4)
b, Å	12.9319(8)	13.0505(6)	13.1470(5)
c, Å	19.0732(10)	10.7236(4)	19.1636(15)
α, deg	–	–	–
β, deg	105.739(4)	104.995(7)	105.497(6)
γ, deg	–	–	–
V, Å^3	2608.4(2)	2597.6(2)	2680.4(3)
Z	4	4	4
D_{calc}, g·cm^{-3}	2.394	2.415	2.341
μ, cm^{-1}	40.646	41.716	40.428
Reflections measured	14384	29083	14833
Independent reflections	2392	4743	2452
Reflections used	2392	4743	2452
R_{int}	0.1104	0.1439	0.1154
R_1 [a]	0.0532	0.0686	0.0534
R_{all}	0.0613	0.0990	0.0673
$R_w(F_2)$ [a]	0.1389	0.1743	0.1430

GOF	1.059	1.102	1.023
CCDC	2482310	2482313	2482314

[a] $R_1 = \Sigma||F_o| - |F_c|| / \Sigma |F_o|$ and $R_w = (\Sigma\omega(|F_o| - |F_c|)^2 / \Sigma\omega F_o^2)^{1/2}$.

Sorption isotherm. Adsorption isotherms of benzene and toluene vapors were measured using BELSORP-maxII (MicrotracBEl) at 298 and 313 K, respectively. The benzene and toluene used for the measurements were subjected to three degassing treatments before measurement to remove dissolved gases, and each sample was vacuum dried at 423 K for 12 hours before measurement.

Theoretical calculation. DFT calculations were performed using Gaussian16W from Gaussian Inc. B3LYP/3-21G* was used as the calculation level for structure optimization and energy calculation. The atomic coordinates used for the calculations were those obtained from single crystal structure analysis at 100 K. In structure optimization, it was confirmed that the structure was the most stable structure with no imaginary vibrations by frequency analysis. The stabilization energy due to inclusion was calculated from $\Delta E = -E_{HG}$ / (number of host molecules). E_{HG} is the interaction energy of the host-guest crystal, which was determined by extracting the structure of the host-guest complex from the crystal structure and calculating the energy using the counterpoise method, with GD3BJ used as the dispersion force correction term.

Relative stability of the guest benzene molecules in **2**·3Bz were evaluated by using DFT calculations. The calculations were performed using OpenMX software (Ver. 3.9.9) based on optimized localized basis functions and pseudopotentials (PPs). The basis functions used were H6.0-s2p1, C6.0-s2p2d1, S7.0-s2p2d1f1, and I7.0-s3p2d2f1 for hydrogen, carbon, sulfur, and iodine, respectively; in the abbreviation of basis functions such as C6.0-s2p2d1, C is the atomic symbol, 6.0 represents the cutoff radius (bohr) in the generation by the confinement scheme, and s2p2d1 indicates the employment of two, two, and one optimized radial functions for the s-, p-, and d-orbitals, respectively. The radial functions were optimized by a variational optimization method.[3,4] As valence electrons in the PPs, we included 1s for hydrogen; 2s and 2p for carbon, nitrogen and oxygen; 2s, 2p, and 3s for sodium. All the PPs and pseudo-atomic orbitals used in this study were taken from the database (2019) on the OpenMX website, which was benchmarked by the delta gauge method.[5] Real space grid techniques were used for the numerical integrations and the solution of the Poisson equation using fast Fourier transform with an energy cutoff of 300 Ryd.[6] We used a generalized gradient approximation (GGA) proposed by Perdew, Burke, and Ernzerhof to the exchange–correlation functional.[7] An electronic temperature of 300 K was

used to count the number of electrons by the Fermi–Dirac function for all the systems considered. For k-point sampling, we used a regular mesh of 1 × 1 × 1. Grimme's DFT-D3 dispersion correction was applied to take into account van der Waals interactions.[8,9]

For the initial state of **2**·3Bz, single-crystal structure was used and only hydrogen atoms were geometrically optimized. The optimized **2**·3Bz structure was divided into two parts: the guest Bz-*X* (*X* = A, B, C) part (= Bz-*X*⊂**2**·3Bz) and the host **2**·2Bz (= **2**·3Bz−Bz-*X*). The interaction energies of the guest Bz-*X* (*X* = A, B, C) with the host **2**·2Bz were evaluated by using the total energies of the optimized **2**·3Bz, the guest Bz-*X*⊂**2**·3Bz (*X* = A, B, C), and the host **2**·3Bz−Bz-*X* structures, where basis set superposition errors (BSSEs) were corrected based on counterpoise method.

$$\Delta E_X = E(\mathbf{2}\cdot 3\mathrm{Bz}) - E(\mathbf{2}\cdot 3\mathrm{Bz\text{-}Bz\text{-}}X) - E(\mathrm{Bz\text{-}}X\subset\mathbf{2}\cdot 3\mathrm{Bz})$$

The calculated ΔE_X values are quite low ($\Delta E_X < -300$ kcal/mol). This is because the hypothetical structures of the host **2**·3Bz−Bz-*X* are not the optimized stable structures for **2**·2Bz and possess drastically different molecular arrangements from the experimentally observed structure of **2**·2Bz (Figure S19). Still, the relative interaction energies and the stability trend can be discussed.

a)

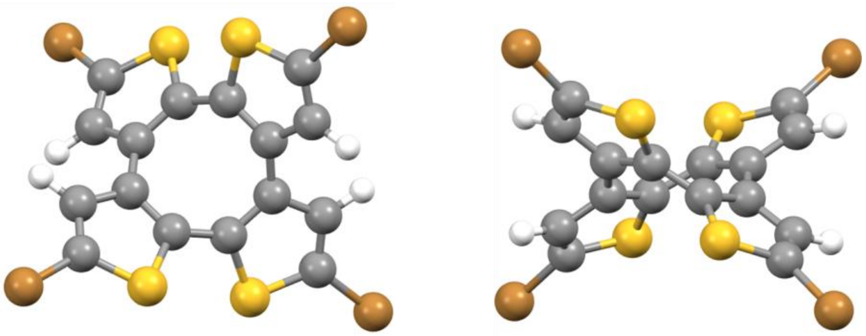

b)

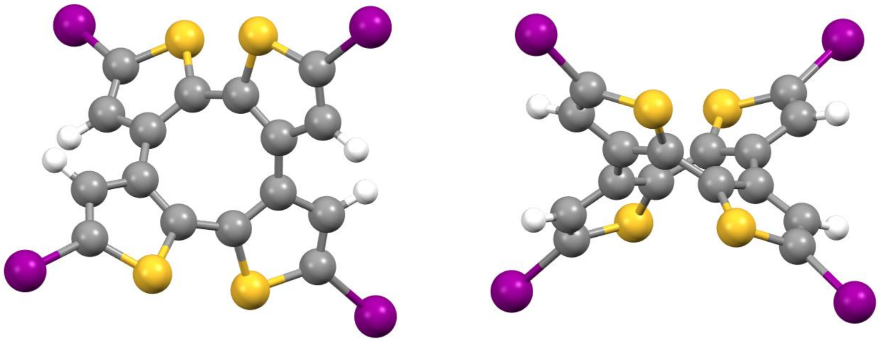

Figure S1. Optimized structures of isolated molecules a) **1** and b) **2** by DFT calculations.

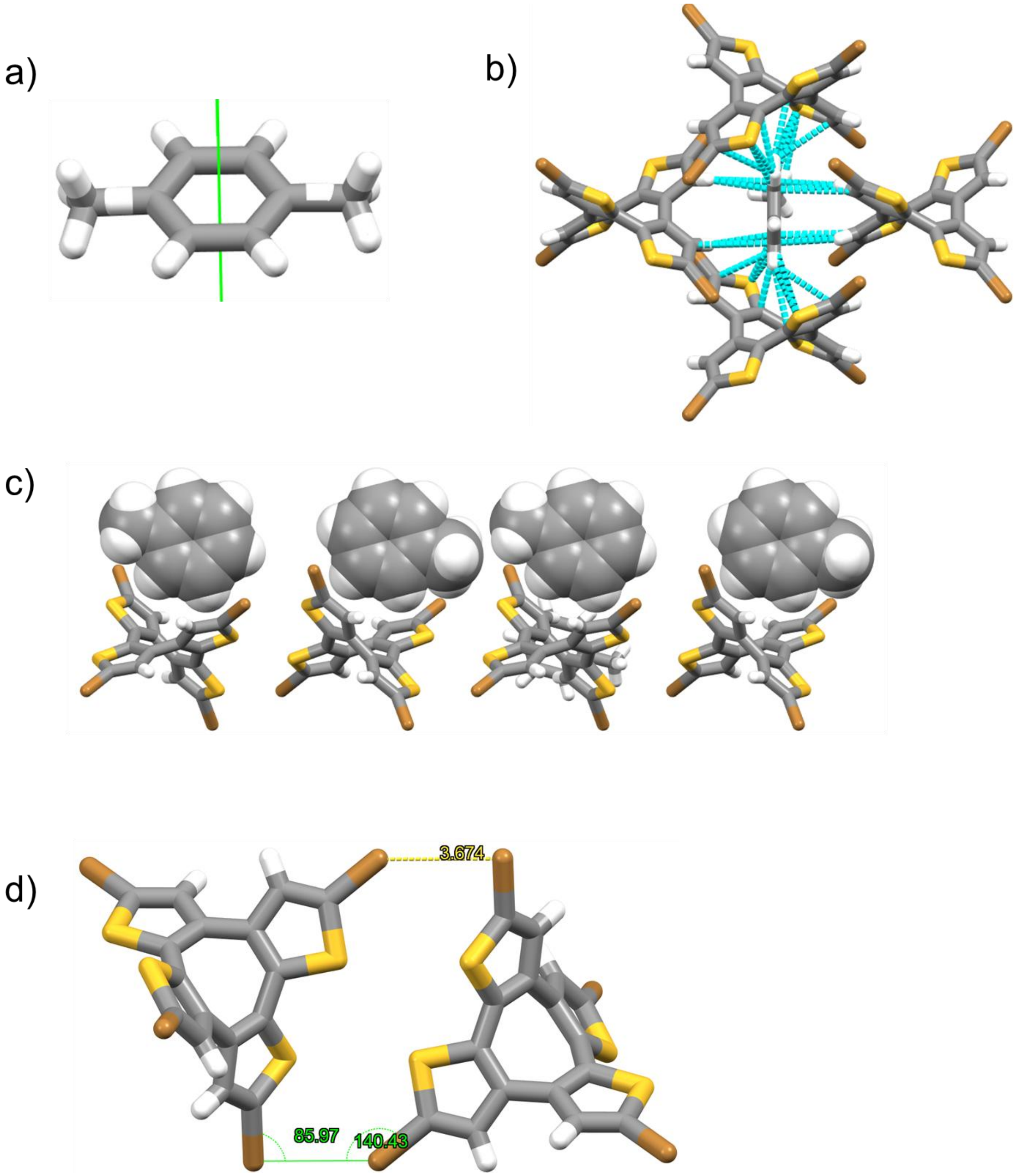

Figure S2. **1**•Tol crystal at 100 K. a) Orientationally disordered Tol molecules (green line: two-fold rotation axis), b) intermolecular interactions of Tol molecules in the channel, and c) molecular arrangement pattern in the channel. d) Br-Br distance (yellow label) and C-Br···Br angle (green label)

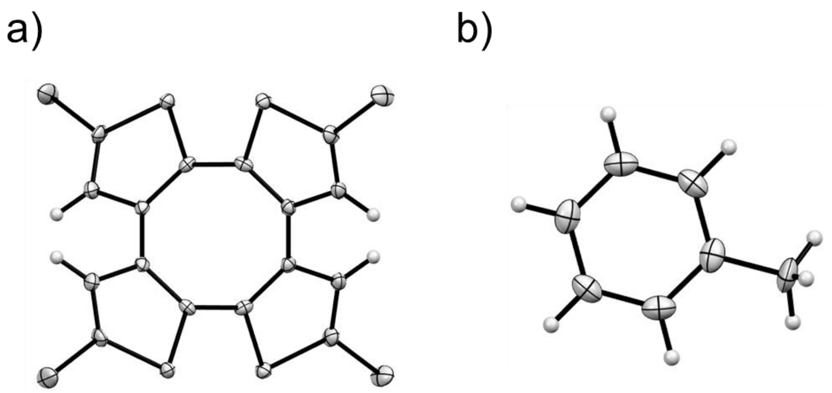

Figure S3. ORTEP diagram of **1**•Tol crystal at 100 K. a) host thienylene and b) guest toluene.

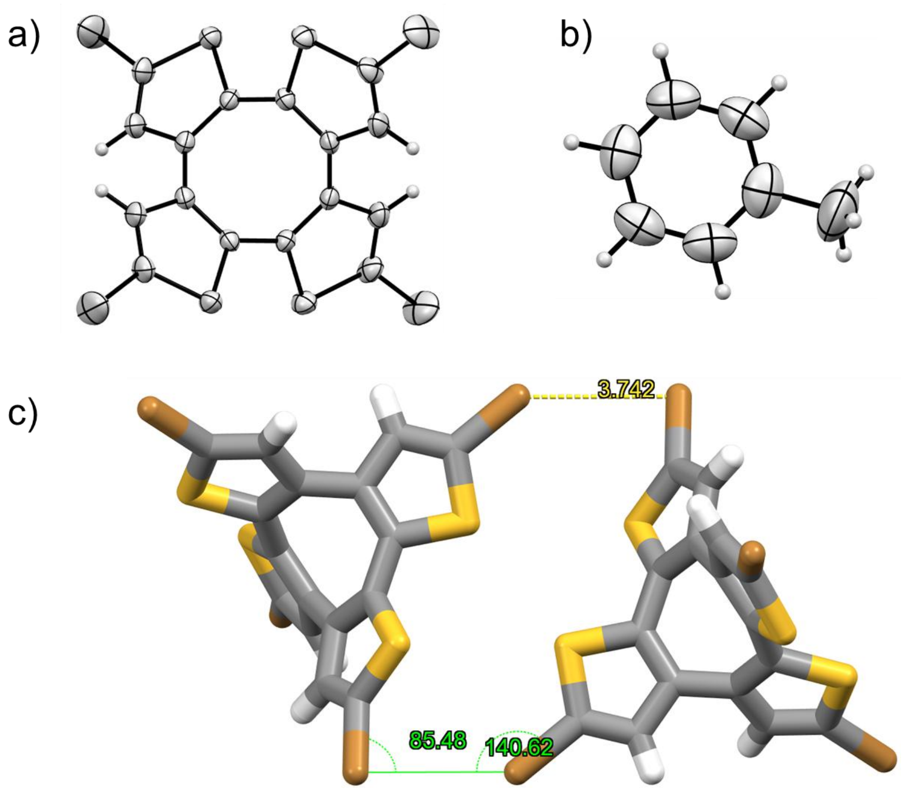

Figure S4. **1**•Tol crystal at 293 K. ORTEP diagram of a) host thienylene and b) guest toluene. c) Br-Br distance (yellow label) and C-Br···Br angle (green label)

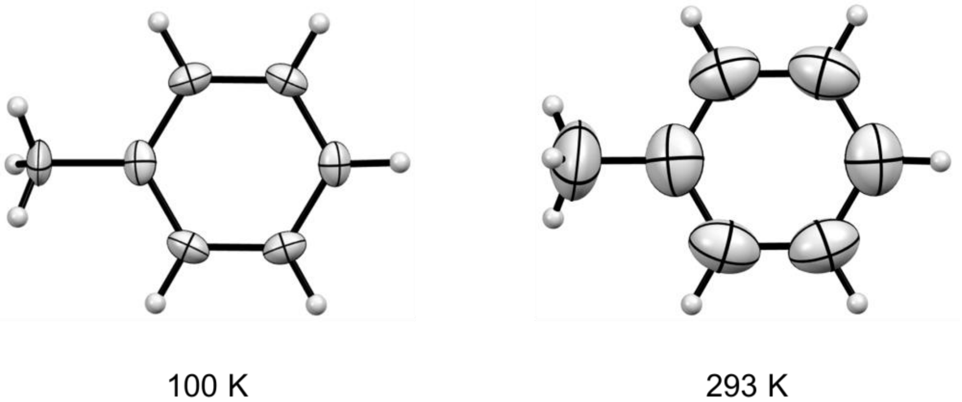

Figure S5. Changes in the temperature factor of Tol molecules in the **1**•Tol crystal at 100 K (left) and 293 K (right).

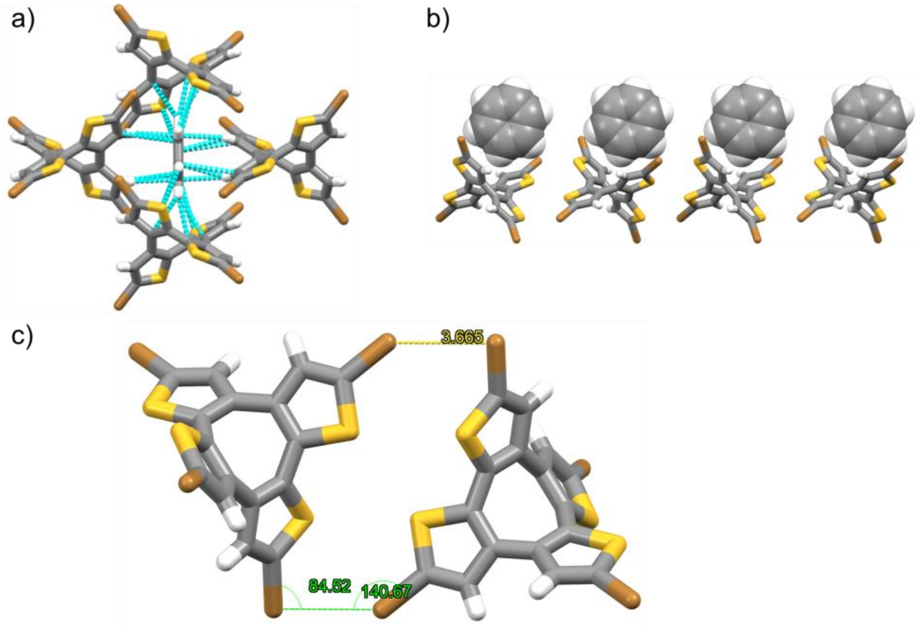

Figure S6. **1**•Bz crystal. a) Intermolecular interactions of Bz molecules in the channel and b) molecular arrangement pattern in the channel. c) Br-Br distance (yellow label) and C-Br···Br angle (green label)

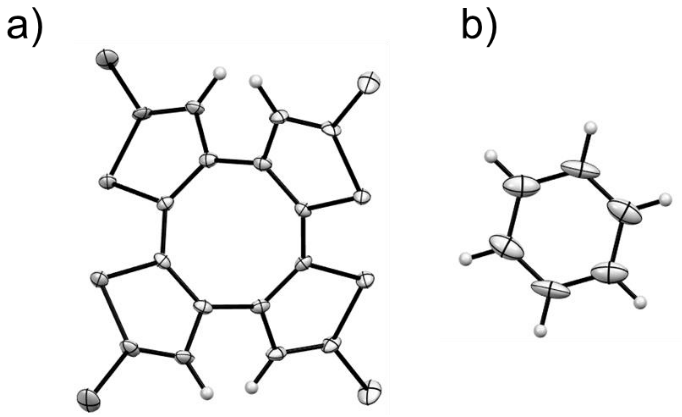

Figure S7. ORTEP diagram of **1**•Bz crystal. a) host thienylene and b) guest benzene.

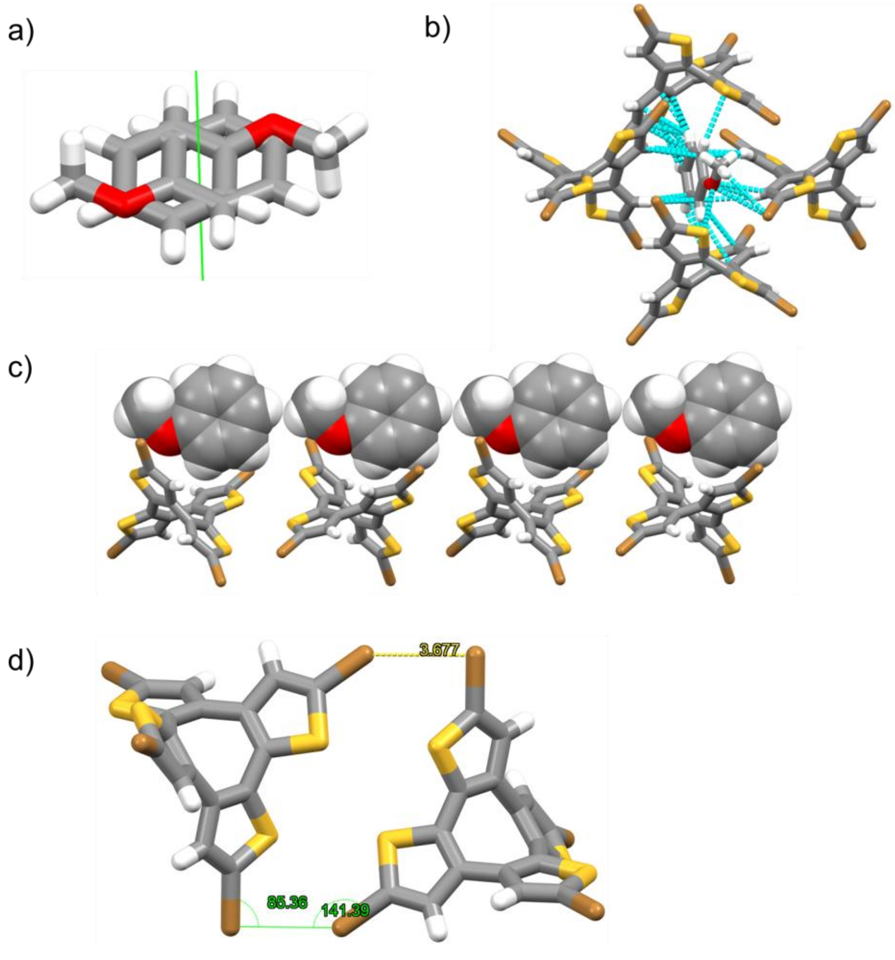

Figure S8. **1**•An crystal. a) Orientationally disordered An molecules (green line: 2-fold rotation axis), b) intermolecular interactions of An molecules in the channel, and c) molecular arrangement pattern in the channel. d) Br-Br distance (yellow label) and C-Br···Br angle (green label)

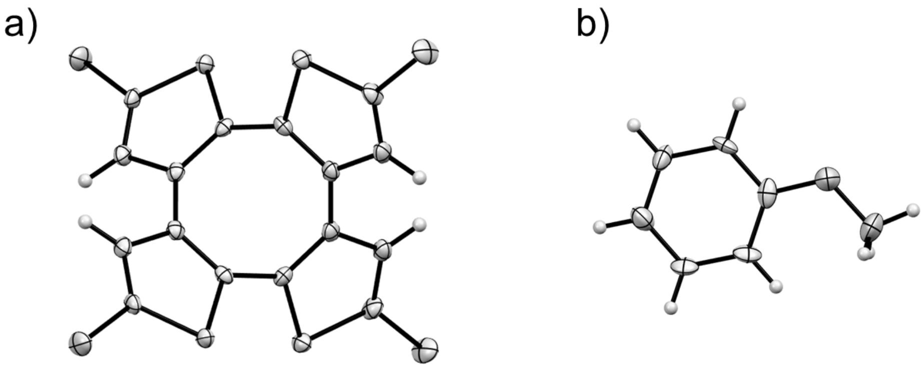

Figure S9. ORTEP diagram of **1**•An crystal. a) host thienylene and b) guest anisole.

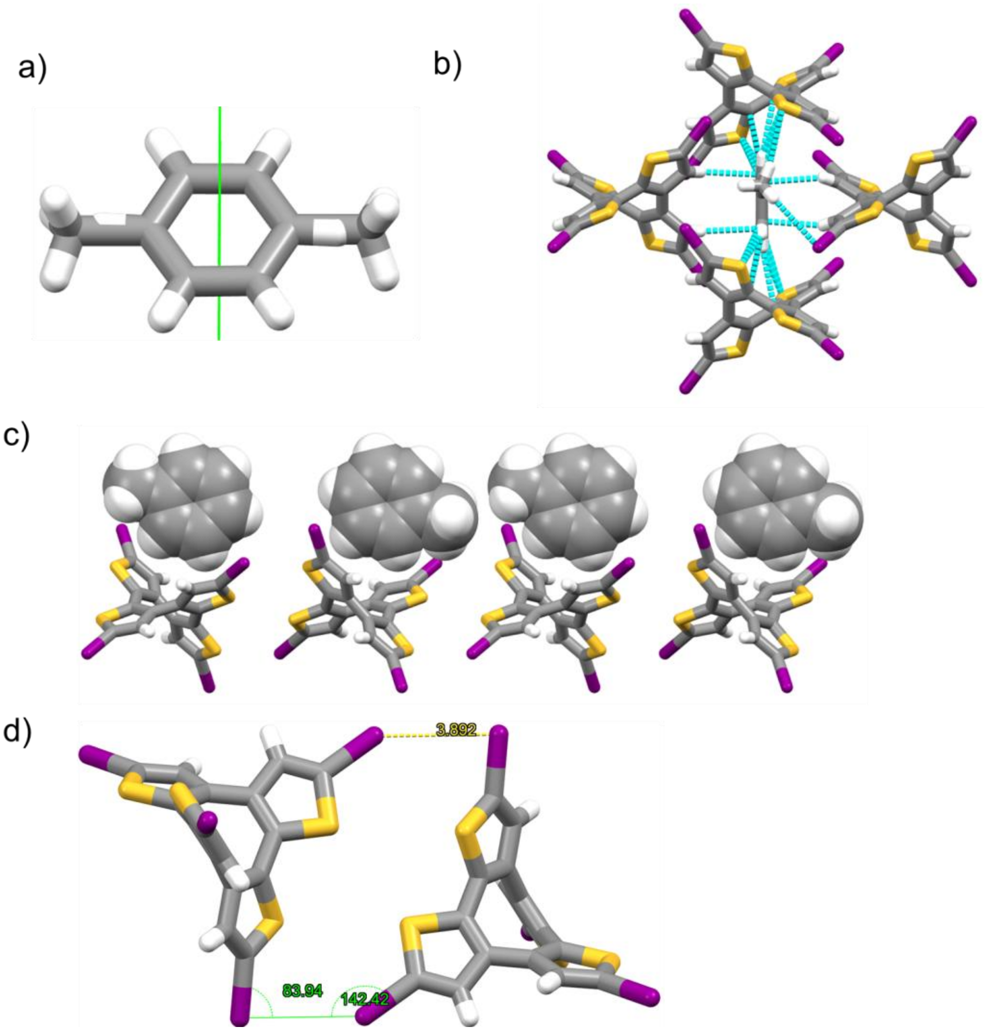

Figure S10. **2**•Tol crystal at 100 K. a) Orientationally disordered Tol molecules (green line: 2-fold rotation axis), b) intermolecular interactions of Tol molecules in the channel, and c) molecular arrangement pattern in the channel. d) I-I distance (yellow label) and C-I···I angle (green label)

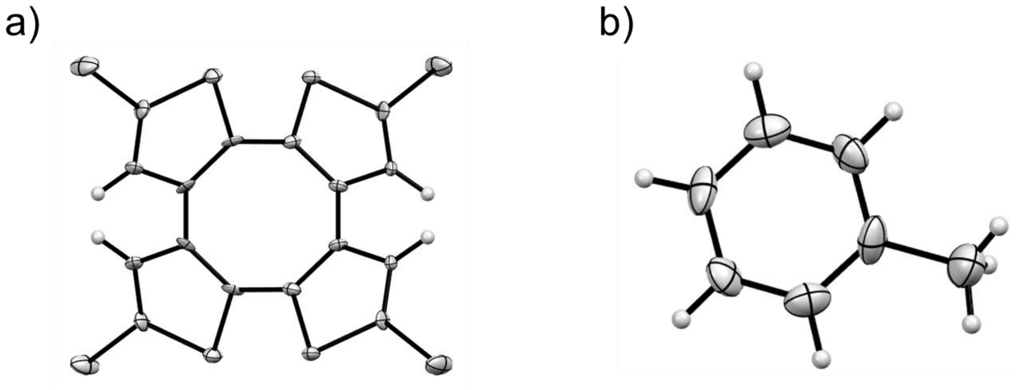

Figure S11. ORTEP diagram of **2**•Tol crystal in 100 K. a) host thienylene and b) guest toluene.

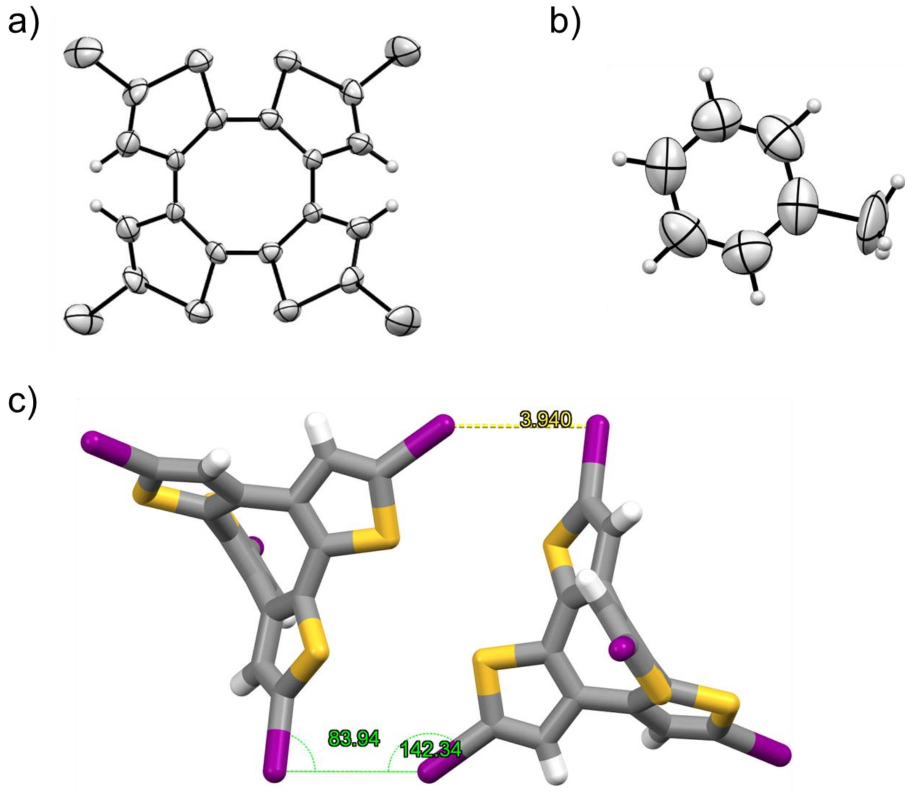

Figure S12. **2**•Tol crystal at 293 K. ORTEP diagram of a) host thienylene and b) guest toluene. c) I-I distance (yellow label) and C-I···I angle (green label)

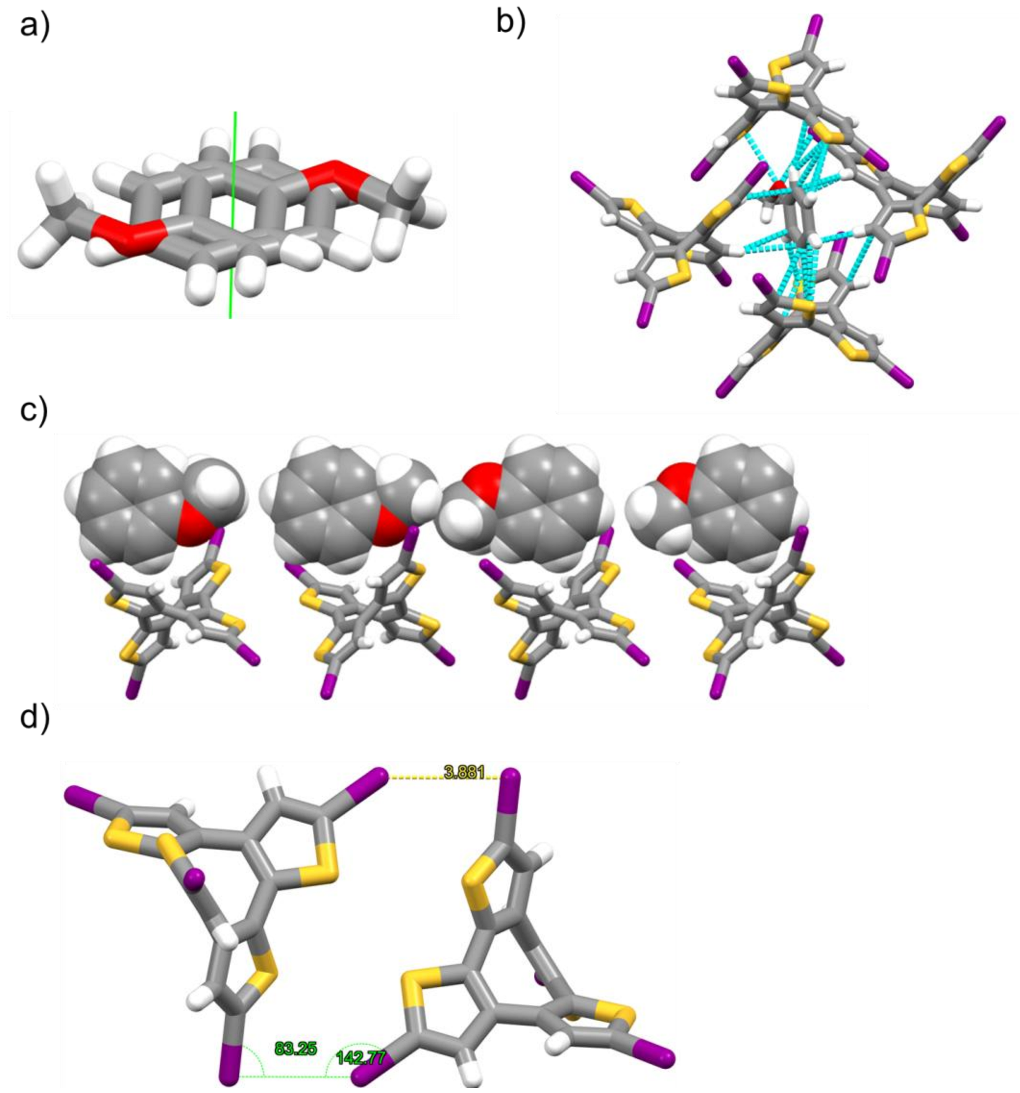

Figure S13. **2**•An crystal. a) Orientationally disordered An molecules (green line: 2-fold rotation axis), b) intermolecular interactions of An molecules in the channel, and c) molecular arrangement pattern in the channel. d) I-I distance (yellow label) and C-I···I angle (green label)

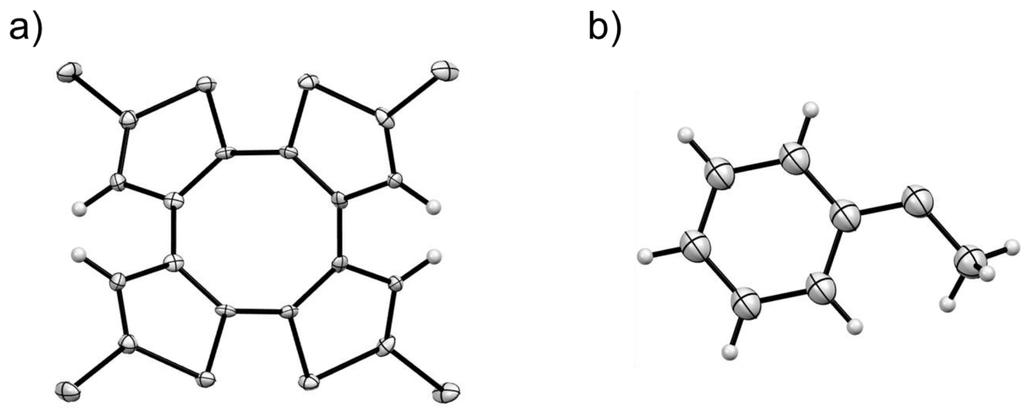

Figure S14. ORTEP diagram of **2**•An crystal. a) host thienylene and b) guest anisole.

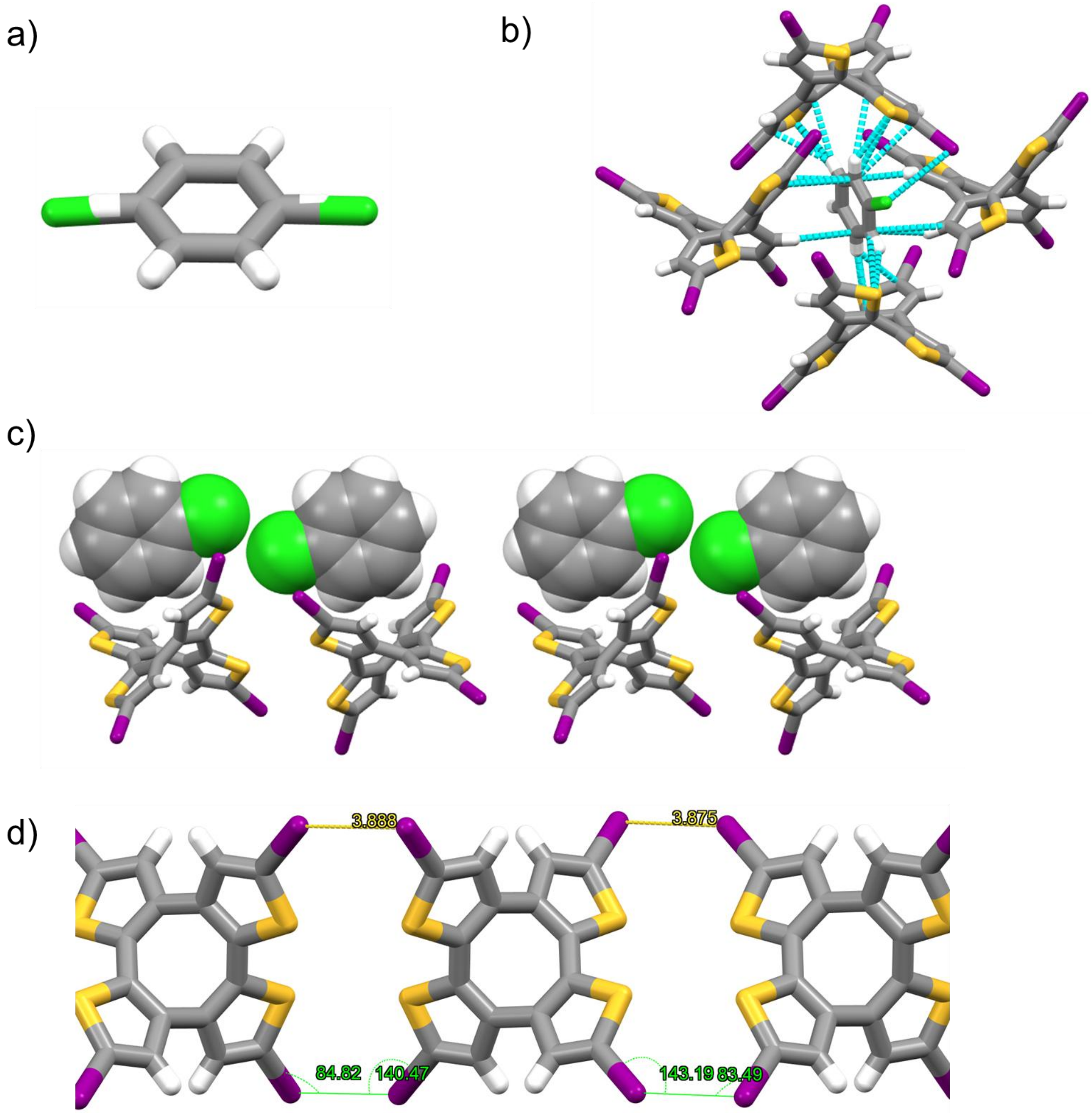

Figure S15. **2**•ClBz crystal at 100 K. a) Orientationally disordered ClBz molecules, b) intermolecular interactions of ClBz molecules in the channel, and c) molecular arrangement pattern in the channel. d) I-I distance (yellow label) and C-I···I angle (green label)

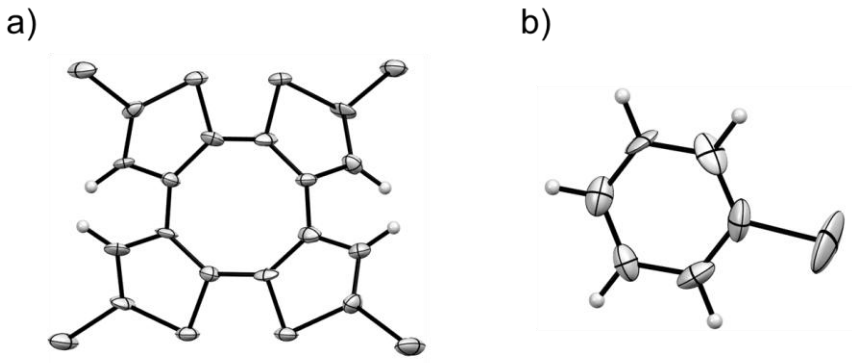

Figure S16. ORTEP diagram of **2**•ClBz crystal at 100 K. a) host thienylene and b) guest chlorobenzene.

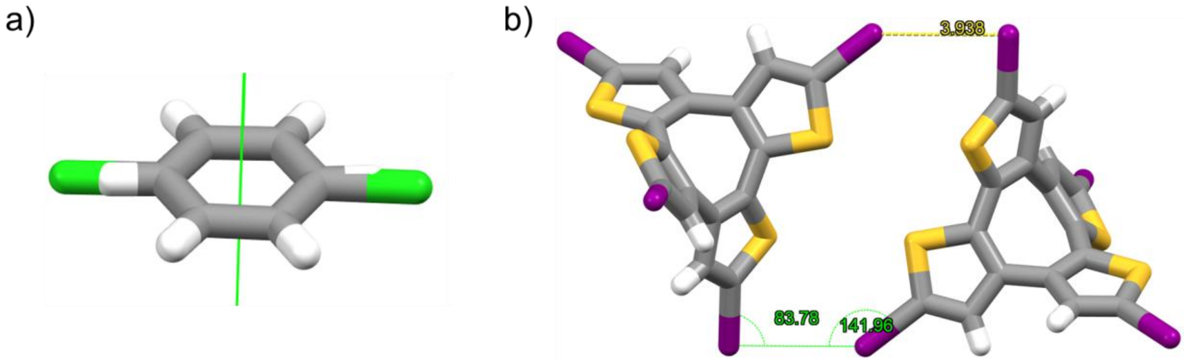

Figure S17. **2**•ClBz crystal at 293 K. a) Orientationally disordered ClBz molecules (green line: 2-fold rotation axis), b) I-I distance (yellow label) and C-I···I angle (green label)

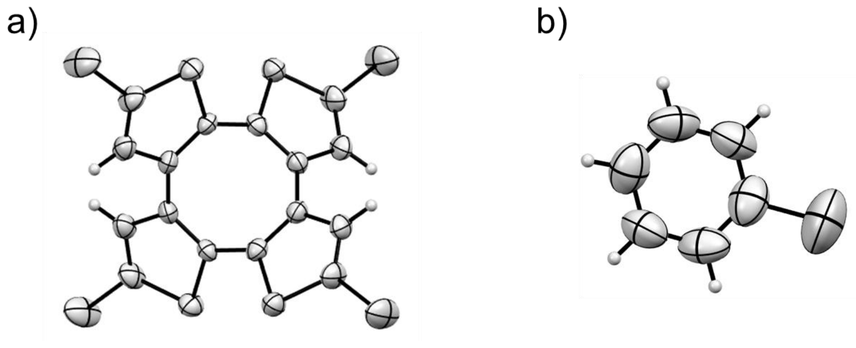

Figure S18. ORTEP diagram of **2**•ClBz crystal at 293 K. a) host thienylene and b) guest chlorobenzene

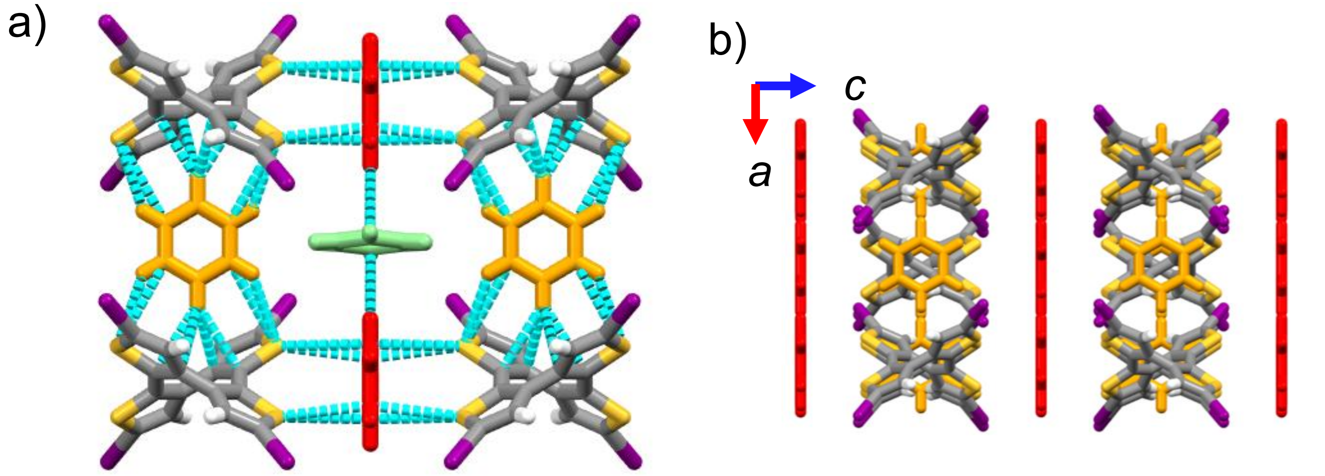

Figure S19. Crystal structures of **2**•3(Bz) and **2**•2(Bz). a) Bz-A, Bz-B, and Bz-C molecules of **2**•3(Bz). b) Molecular arrangement pattern viewed along the *a*-axis of **2**•2(Bz) after Bz-C desorption.

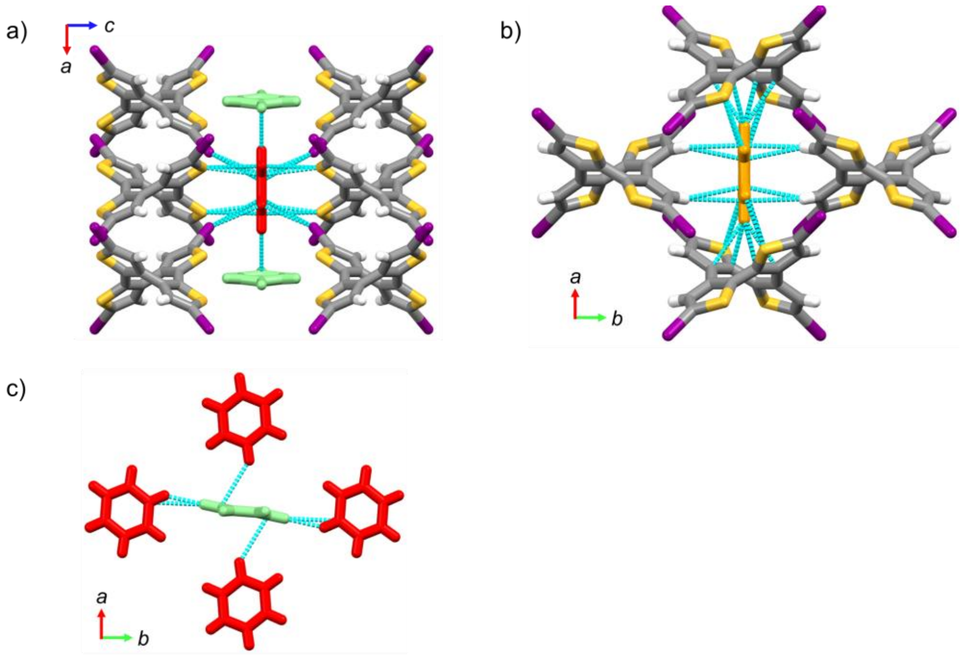

Figure S20. Short contacts around guest molecules of **2**•3(Bz). a) around Bz-A. C-H···π (3.071, 3.110 Å) and other contacts was observed. b) around Bz-B. C-H···π (2.974, 3.028, 3.058, 3.148 Å) and other contacts was observed. c) around Bz-C. C-H···π (3.071 – 3.110 Å) and other contacts was observed.

Table S2. Summary of X-X distance and C-X···X angle in type A crystals.

Host-guest crystals	X-X distance (Å)	C-X…X angle (°)
**1**•Bz	3.665	84.52, 140.67
**1**•Tol (100 K)	3.674	85.97, 140.43
**1**•Tol (293 K)	3.742	85.48, 140.62
**1**•An	3.677	85.36, 141.39
**2**•Tol (100 K)	3.892	83.94, 142.42
**2**•Tol (293 K)	3.940	83.94, 142.34
**2**•An	3.881	83.25, 142.77
**2**•ClBz (100 K)	3.888 / 3.875	84.82, 140.47 / 83.49, 143.19
**2**•ClBz (293 K)	3.938	83.78, 141.96

Table S3. The calculated total energies (per formula) of the optimized **2**·3Bz, the guest Bz-*X*⊂**2**·3Bz (*X* = A, B, C), and the host **2**·3Bz−Bz-*X* structures.

	Bz-A	Bz-B	Bz-C
E(**2**·3Bz) / kcal mol^{-1}	−770659.833	−770659.833	−770659.833
E(**2**·3Bz−Bz-*X*) / kcal mol^{-1}	−672857.421	−672832.052	−672868.517
E(Bz-*X*⊂**2**·3Bz) / kcal mol^{-1}	−96477.669	−96490.080	−96468.709
Δ*E* / kcal mol^{-1}	−331.186	−334.425	−330.652
Δ*E*−Δ*E*(Bz-C) / kcal mol^{-1}	−0.534	−3.774	0

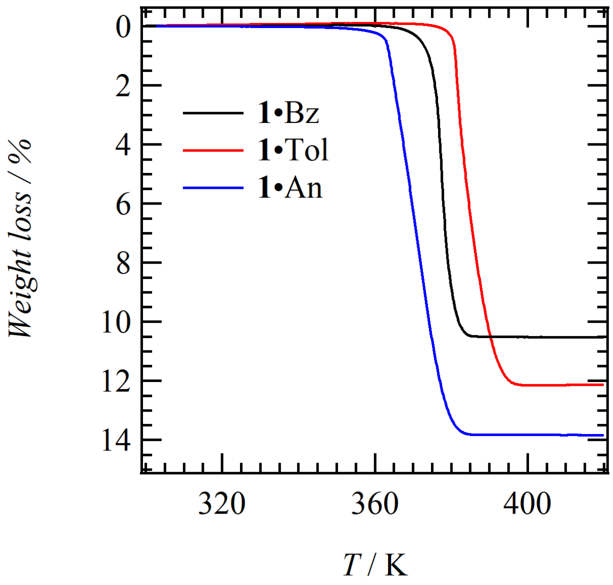

Figure S21. TG charts of **1**•Bz, **1**•Tol, and **1**•An.

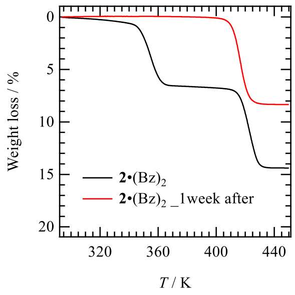

Figure S22. TG charts of **2**•2(Bz).

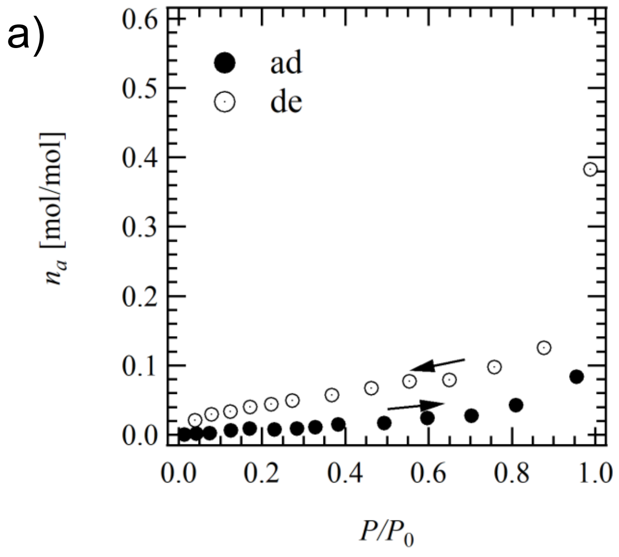

Figure S23. Adsorption isotherms of a) **1** and b) **2** for Bz at 298 K.

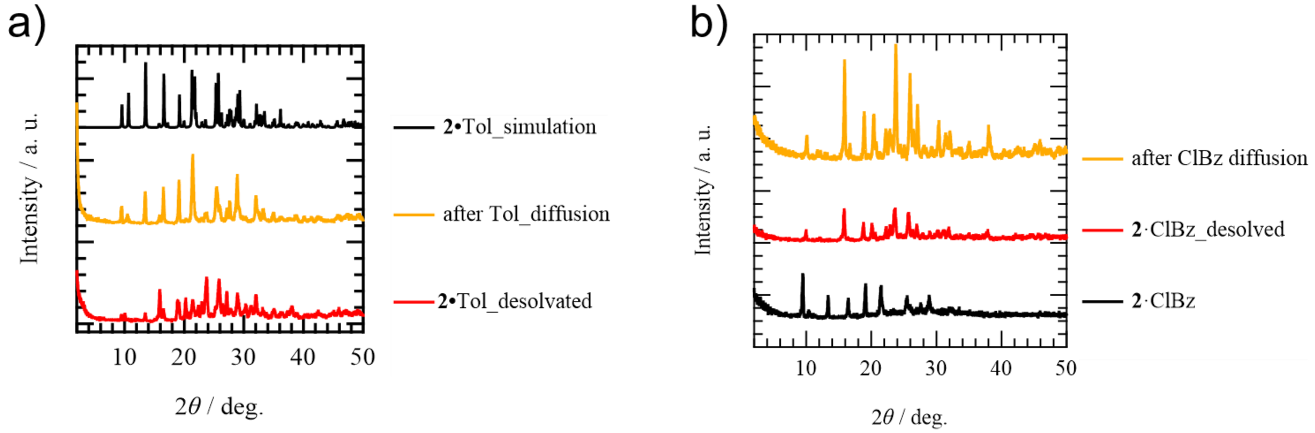

Figure S24. Structural changes due to re-adsorption of a) **2**•Tol and b) **2**•ClBz.

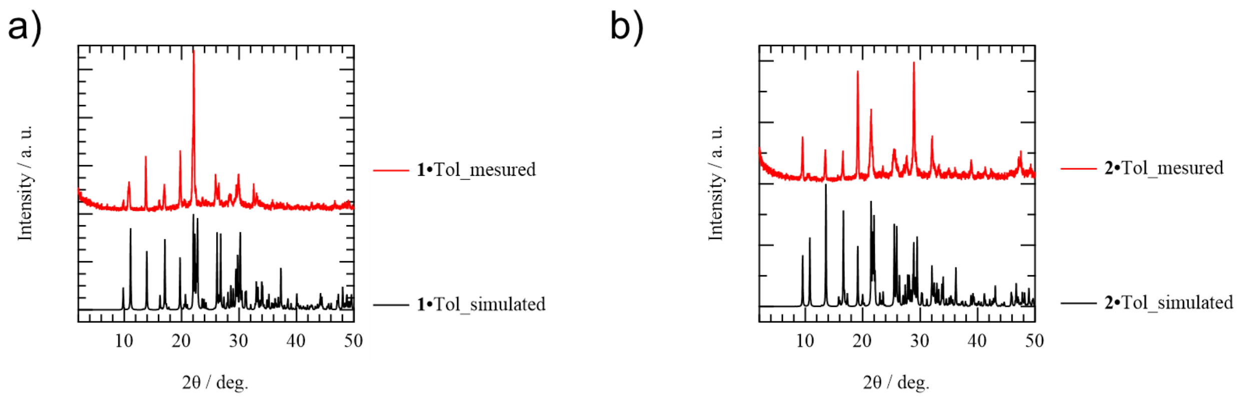

Figure S25. Comparison of PXRD patterns at 293 K and simulated XRD patterns calculated from single crystal X-ray crystallographic analysis at 100 K for a) **1**•Tol and b) **2**•Tol crystals.

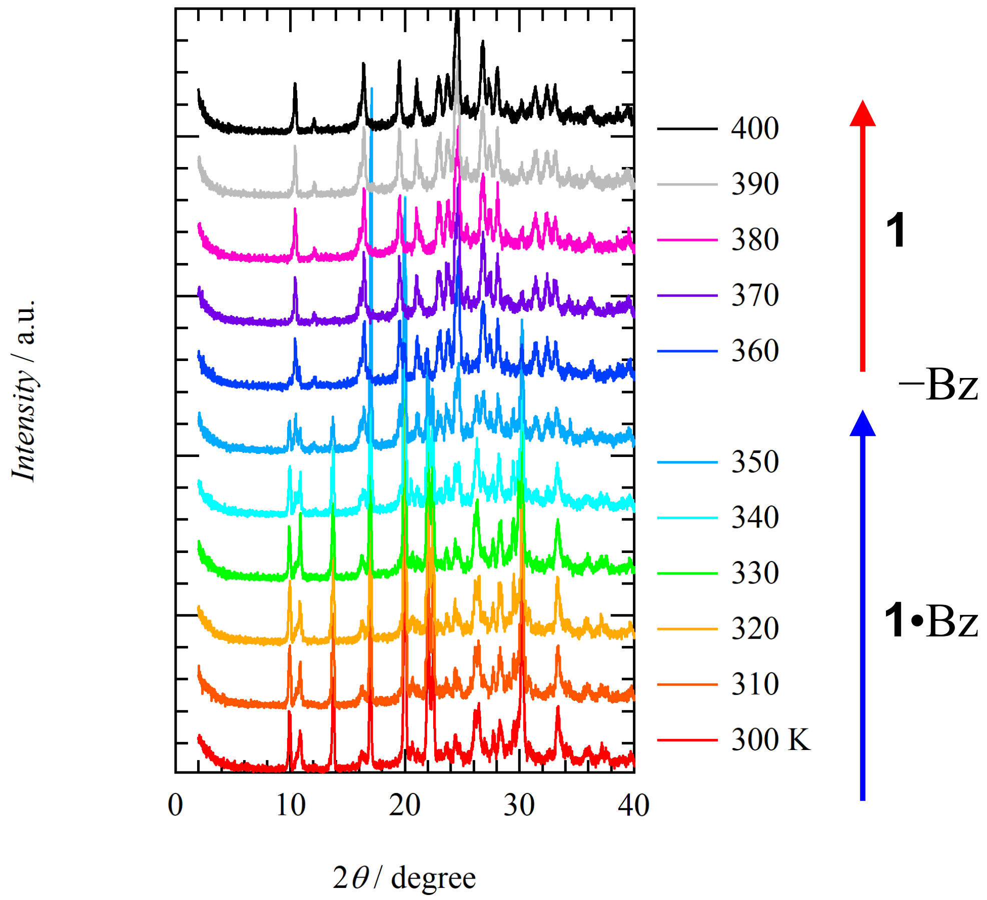

Figure S26. Temperature variable PXRD patterns of **1**•Bz.

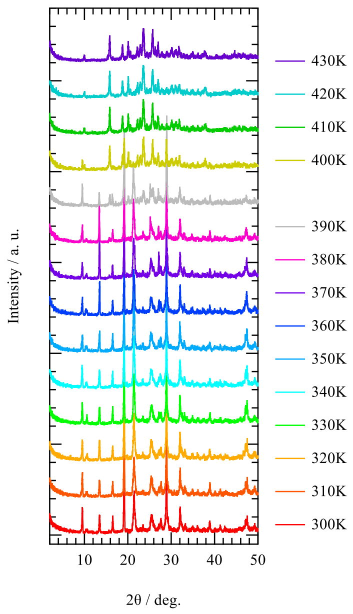

Figure S27. Temperature variable PXRD patterns of **2**•Tol.

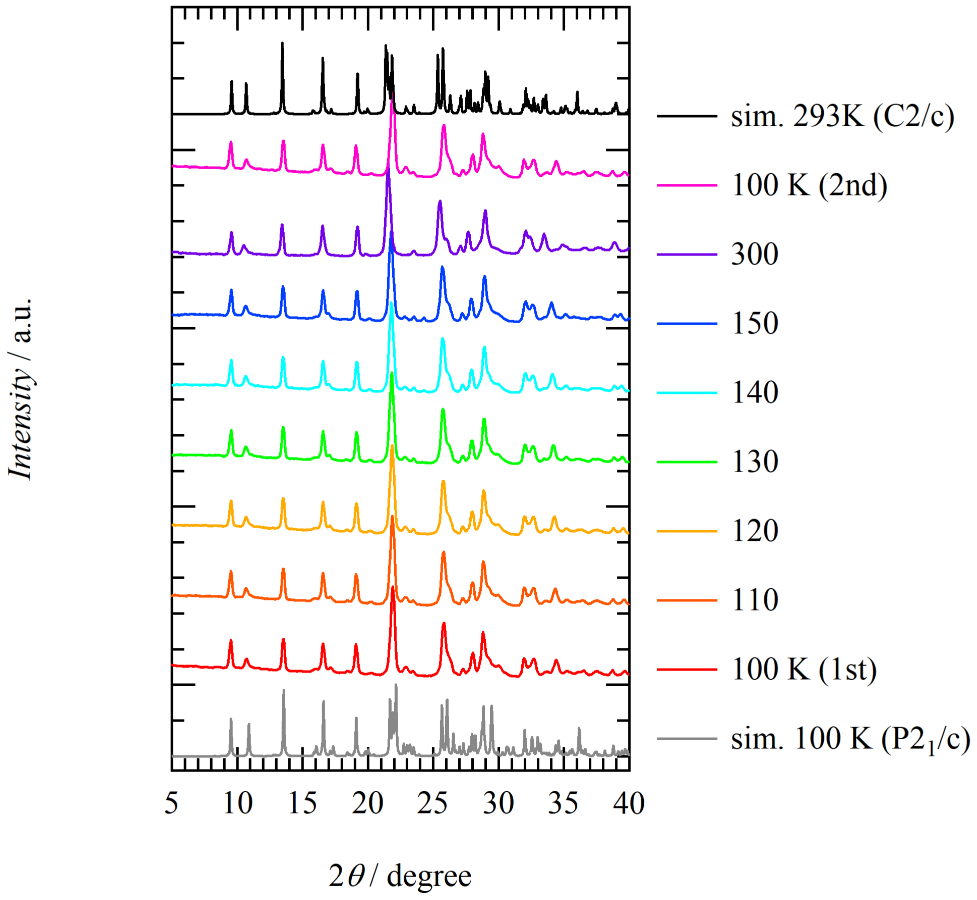

Figure S28. Temperature variable PXRD patterns of **2**•ClBz.

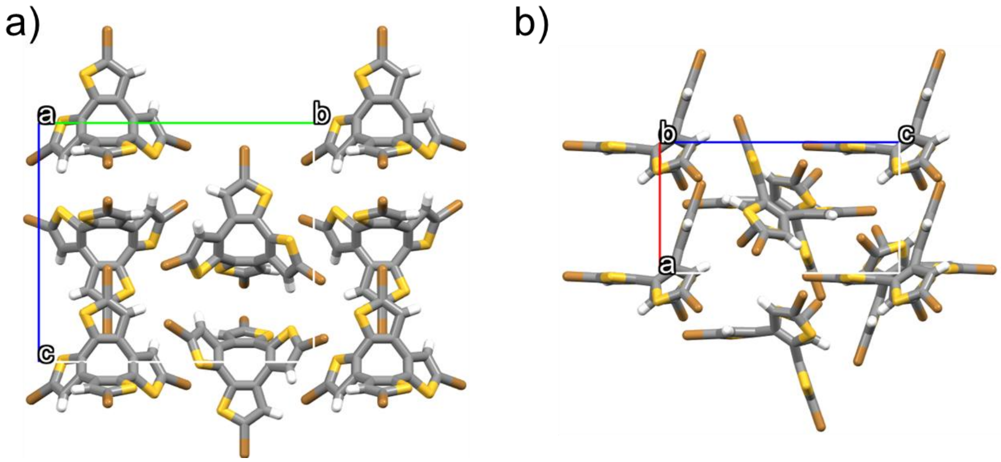

Figure S29. Crystal structure of **1**. Unit cell viewed along the a) *a*-axis and b) *b*-axis.

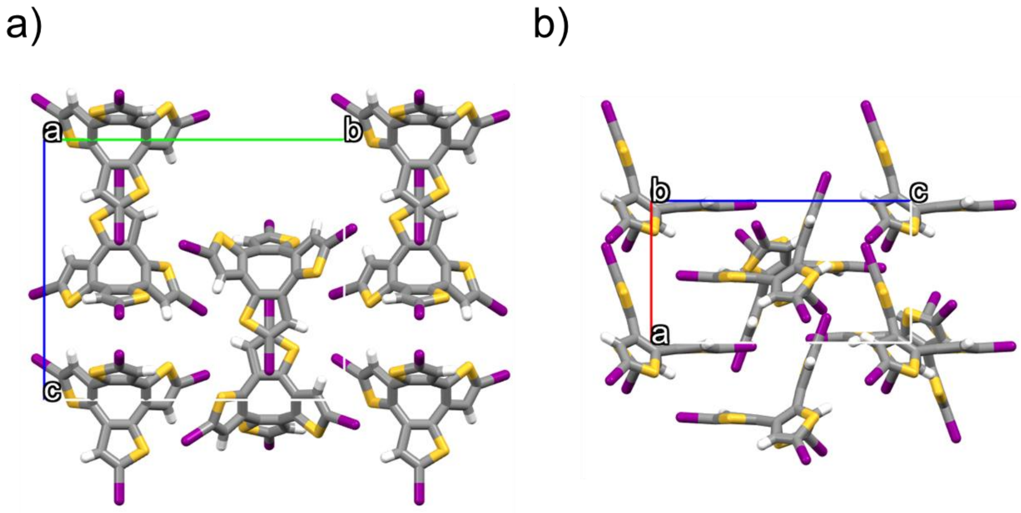

Figure S30. Crystal structure of **2**. Unit cell viewed along the a) *a*-axis and b) *b*-axis.

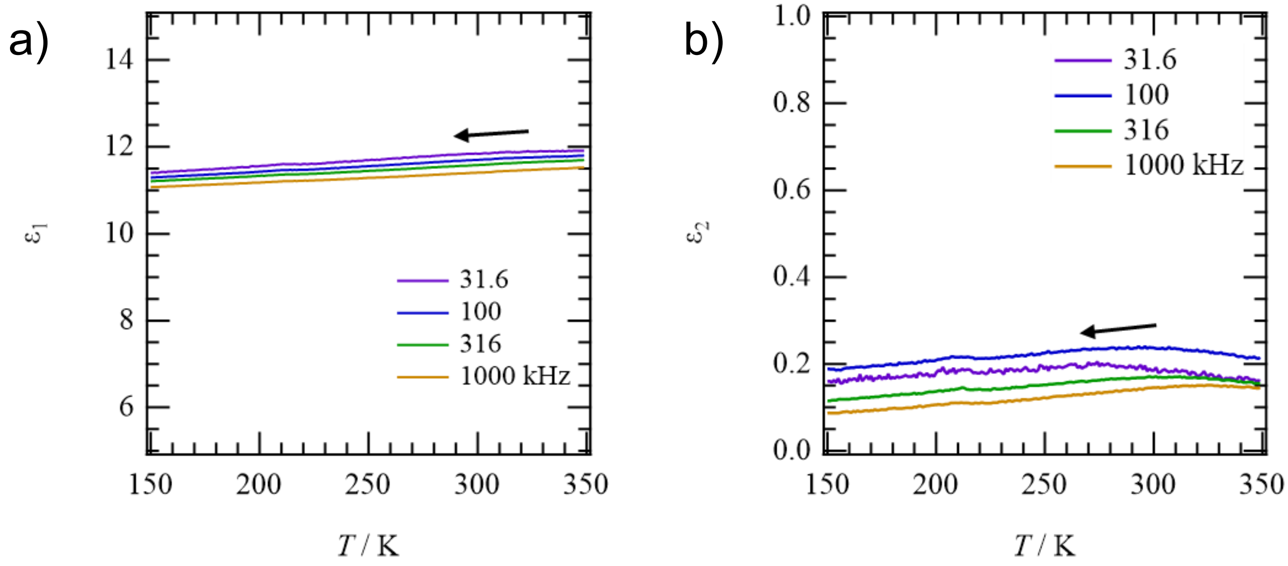

Figure S31. a) Real part and b) imaginary part of the dielectric constant of **1**•Bz.

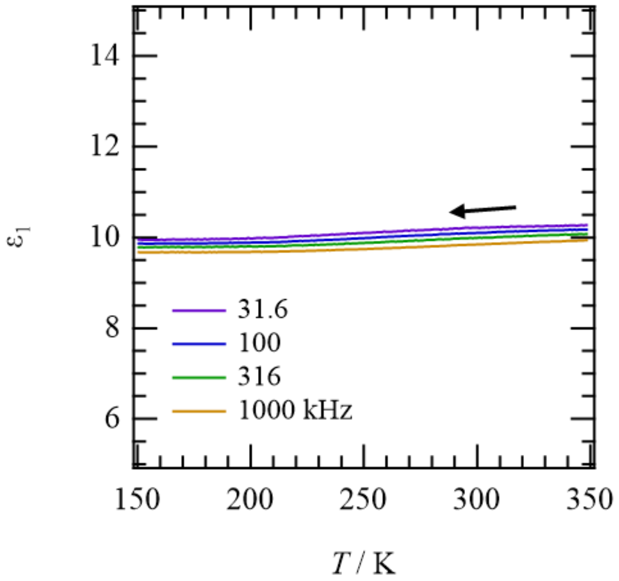

Figure S32. Real part of the dielectric constant of **1**•Tol.

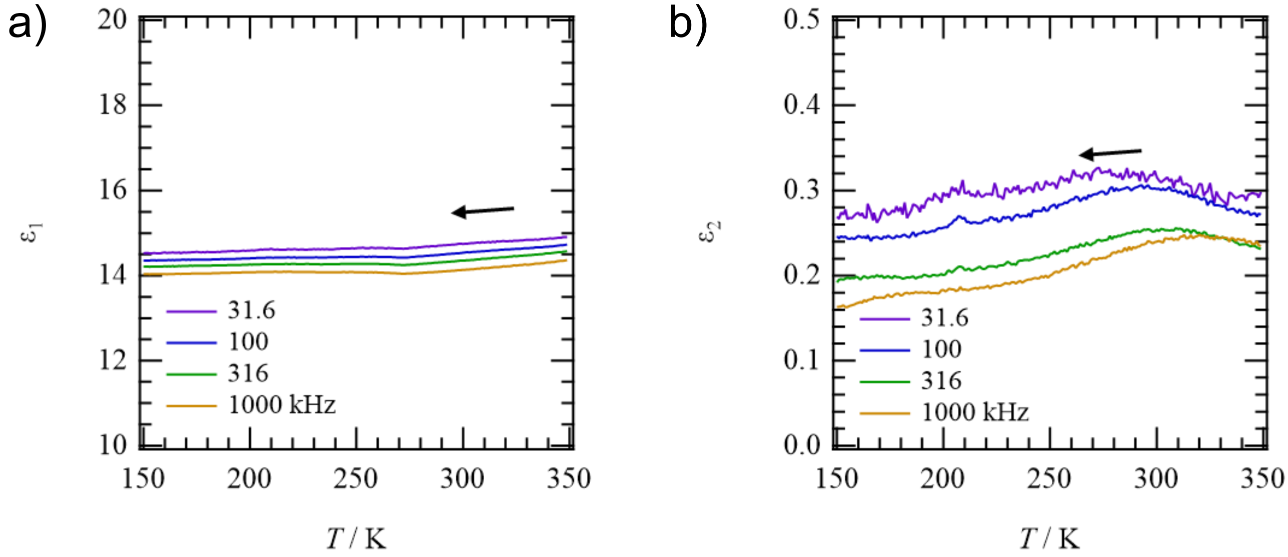

Figure S33. a) Real part and b) imaginary part of the dielectric constant of **1**• An.

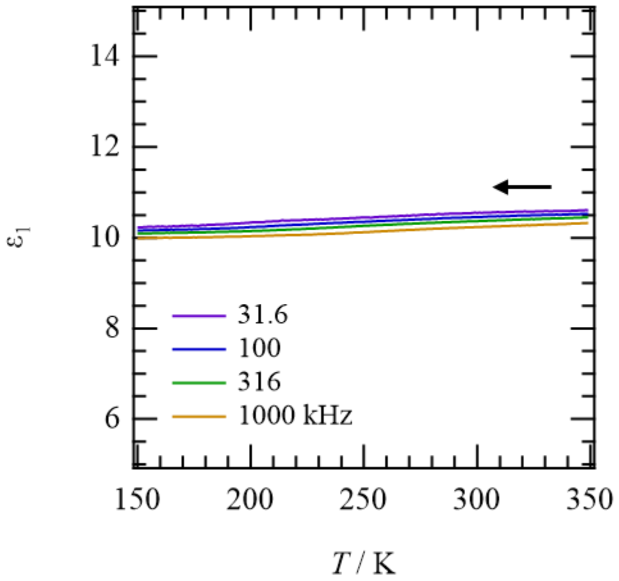

Figure S34. Real part dielectric constant of **2**•Tol.

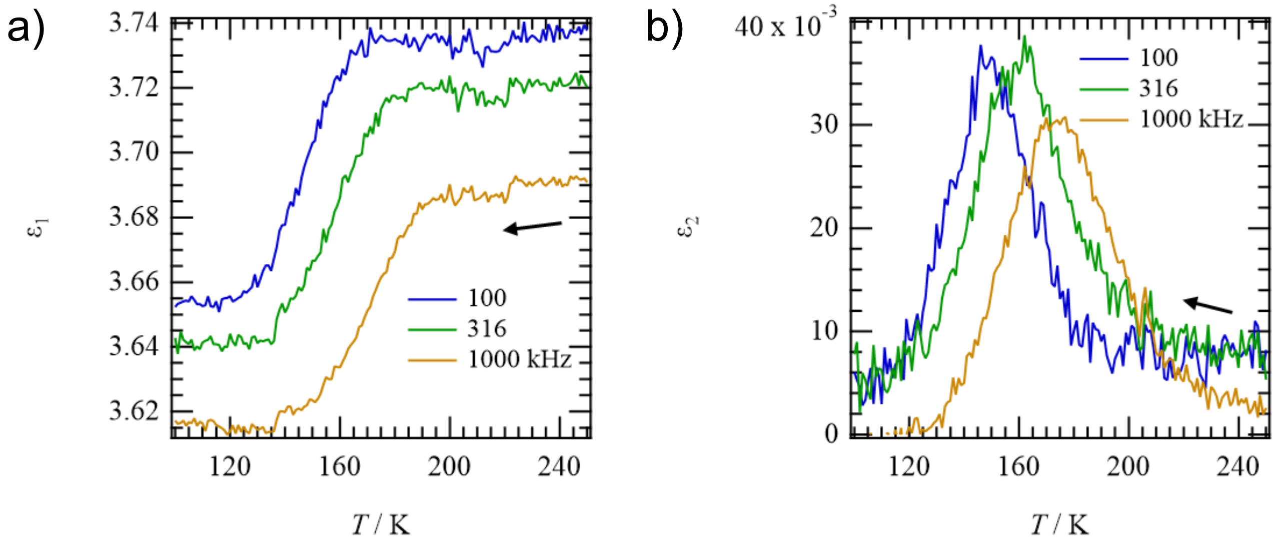

Figure S35. a) Real part and b) imaginary part of the dielectric constant of **2**•An.

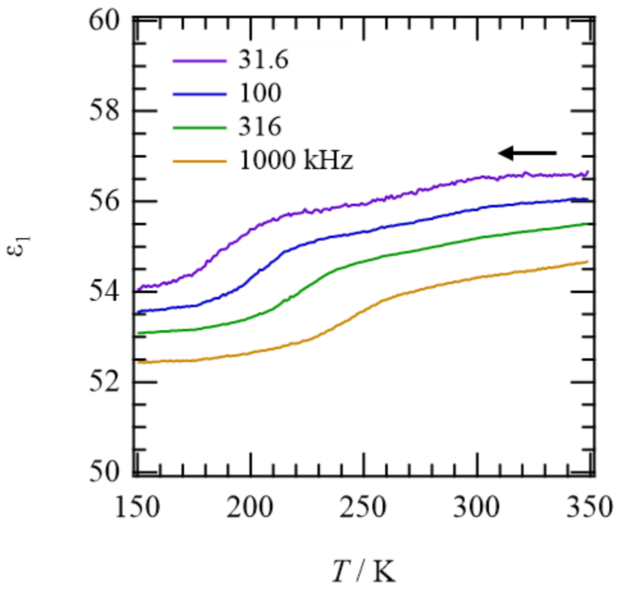

Figure S36. Real part dielectric constant of **2**•ClBz.

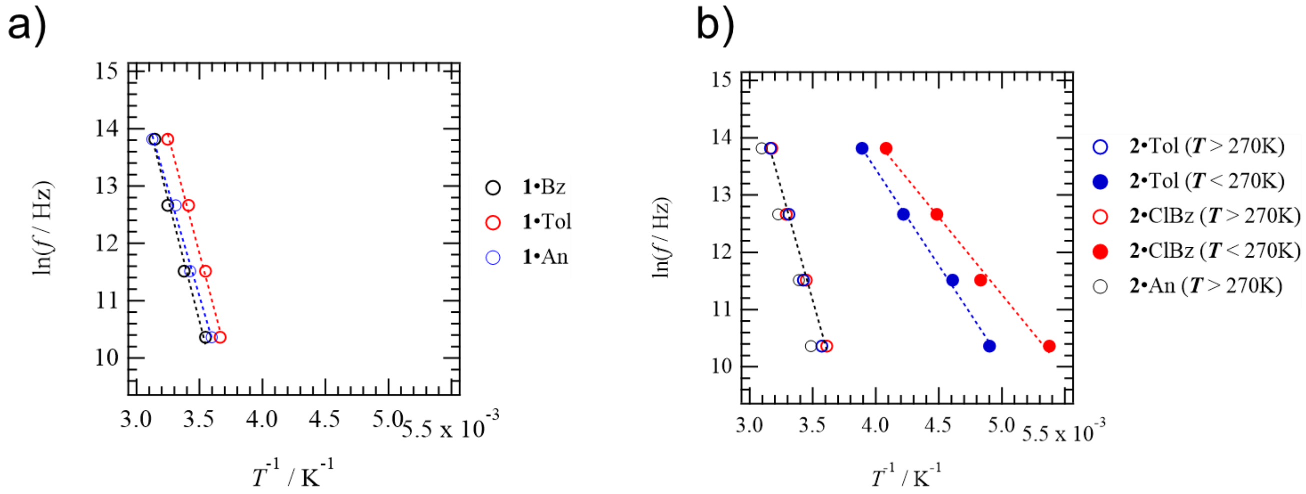

Figure S37. ln $f - T^{-1}$ plots of a) **1**•Bz, **1**•Tol, and **1**•An, b) **2**•Tol, **2**•An, and **2**•ClBz.

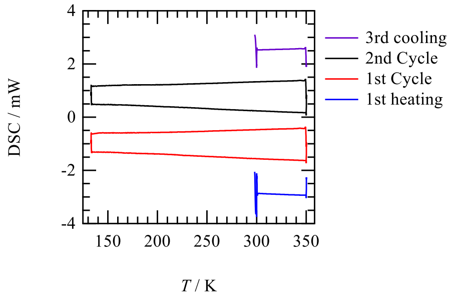

Figure S38. DSC chart of **2•**ClBz.

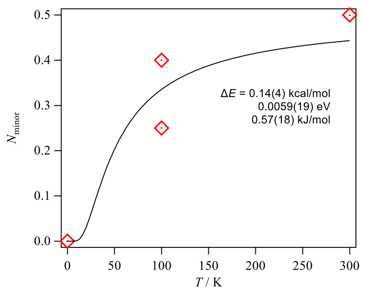

Figure S39. Analysis of the occupancy of ClBz in **2**·ClBz single crystal using a model assuming a Boltzmann distribution of a two-level model.[S3]

References

Frisch, M. J.; Trucks, G. W.; Schlegel, H. B.; Scuseria, G. E.; Robb, M. A.; Cheeseman, J. R.; Scalmani, G.; Barone, V.; Petersson, G. A.; Nakatsuji, H.; Li, X.; Caricato, M.; Marenich, A. V.; Bloino, J.; Janesko, B. G.; Gomperts, R.; Mennucci, B.; Hratchian, H. P.; Ortiz, J. V.; Izmaylov, A. F.; Sonnenberg, J. L.; Williams; Ding, F.; Lipparini, F.; Egidi, F.; Goings, J.; Peng, B.; Petrone, A.; Henderson, T.; Ranasinghe, D.; Zakrzewski, V. G.; Gao, J.; Rega, N.; Zheng, G.; Liang, W.; Hada, M.; Ehara, M.; Toyota, K.; Fukuda, R.; Hasegawa, J.; Ishida, M.; Nakajima, T.; Honda, Y.; Kitao, O.; Nakai, H.; Vreven, T.; Throssell, K.; Montgomery Jr., J. A.; Peralta, J. E.; Ogliaro, F.; Bearpark, M. J.; Heyd, J. J.; Brothers, E. N.; Kudin, K. N.; Staroverov, V. N.; Keith, T. A.; Kobayashi, R.; Normand, J.; Raghavachari, K.; Rendell, A. P.; Burant, J. C.; Iyengar, S. S.; Tomasi, J.; Cossi, M.; Millam, J. M.; Klene, M.; Adamo, C.; Cammi, R.; Ochterski, J. W.; Martin, R. L.; Morokuma, K.; Farkas, O.; Foresman, J. B.; Fox, D. J. *Gaussian 16 Rev. A.03*, Wallingford, CT, 2016.

S1. Koenig, J. D. B.; Laventure, A.; Welch, G. C. Harnessing Direct (Hetero)Arylation in Pursuit of a Saddle-Shaped Perylene Diimide Tetramer. *ACS Appl. Energy Mater.* **2019**, *2* (12), 8939–8945.

S2. Zhao, L.; Qiu, L.; Xia, D.; Liu, S.; Yi, X.; Fan, J.; Lin, K.; Fan, R.; Guo, Y.; Yang, Y. Cyclooctatetrathiophene-Cored Three-Dimensional Hole Transport Material Enabling over 19% Efficiency of Perovskite Solar Cells. *ACS Appl. Energy Mater.* **2019**, *2* (11), 8173–8180.

S3. Ozaki, T. Variationally Optimized Atomic Orbitals for Large-Scale Electronic Structures. *Phys. Rev. B Condens. Matter* **2003**, *67* (15), 155108.

S4. Ozaki, T.; Kino, H. Numerical Atomic Basis Orbitals from H to Kr. *Phys. Rev. B Condens. Matter* **2004**, *69* (19), 195113.

S5. Lejaeghere, K.; Bihlmayer, G.; Bjorkman, T.; Blaha, P.; Blugel, S.; Blum, V.; Caliste, D.; Castelli, I. E.; Clark, S. J.; Dal Corso, A.; de Gironcoli, S.; Deutsch, T.; Dewhurst, J. K.; Di Marco, I.; Draxl, C.; Du ak, M.; Eriksson, O.; Flores-Livas, J. A.; Garrity, K. F.; Genovese, L.; Giannozzi, P.; Giantomassi, M.; Goedecker, S.; Gonze, X.; Granas, O.; Gross, E. K. U.; Gulans, A.; Gygi, F.; Hamann, D. R.; Hasnip, P. J.; Holzwarth, N. A. W.; Iu an, D.; Jochym, D. B.; Jollet, F.; Jones, D.; Kresse, G.; Koepernik, K.; Kucukbenli, E.; Kvashnin, Y. O.; Locht, I. L. M.; Lubeck, S.; Marsman, M.; Marzari, N.; Nitzsche, U.; Nordstrom, L.; Ozaki, T.; Paulatto, L.; Pickard, C. J.; Poelmans, W.; Probert, M. I. J.; Refson, K.; Richter, M.; Rignanese, G.-M.; Saha, S.; Scheffler, M.; Schlipf, M.; Schwarz, K.; Sharma, S.; Tavazza, F.; Thunstrom, P.; Tkatchenko, A.; Torrent, M.; Vanderbilt, D.; van Setten, M. J.; Van Speybroeck, V.; Wills, J. M.; Yates, J. R.; Zhang, G.-X.; Cottenier, S. Reproducibility in Density Functional Theory Calculations of Solids. *Science* **2016**, *351* (6280), aad3000–aad3000.

S6. Ozaki, T.; Kino, H. Efficient Projector Expansion for the ¥textitab initio LCAO Method. *Phys. Rev. B Condens. Matter* **2005**, *72* (4), 045121.

S7. Perdew, J. P.; Burke, K.; Ernzerhof, M. Generalized Gradient Approximation Made Simple. *Phys. Rev. Lett.* **1996**, *77* (18), 3865–3868.

S8. Grimme, S.; Antony, J.; Ehrlich, S.; Krieg, H. A Consistent and Accurate Ab Initio Parametrization of Density Functional Dispersion Correction (DFT-D) for the 94 Elements H-Pu. *J. Chem. Phys.* **2010**, *132* (15), 154104.
S9. Grimme, S.; Ehrlich, S.; Goerigk, L. Effect of the Damping Function in Dispersion Corrected Density Functional Theory. *Journal of Computational Chemistry* **2011**, *32* (7), 1456–1465.
