# Supplementary material for: Porous Organic Frameworks Utilizing Halogen···Halogen Interactions of X4–tetra[2,3]Thienylene (X = Br, I): Guest Dynamics and Dielectric Response
Source: Chemistry. 2025 Nov 10;31(71):e02872. doi: 10.1002/chem.202502872 (PMC12734654; doi:10.1002/chem.202502872)

checkCIF/PLATON report

Structure factors have been supplied for datablock(s) shelx_trans

THIS REPORT IS FOR GUIDANCE ONLY. IF USED AS PART OF A REVIEW PROCEDURE FOR PUBLICATION, IT SHOULD NOT REPLACE THE EXPERTISE OF AN EXPERIENCED CRYSTALLOGRAPHIC REFEREE.

No syntax errors found. CIF dictionary Interpreting this report

Datablock: shelx_trans

Bond precision: C-C = 0.0086 A Wavelength=1.54180

Cell:	a=10.9873(2)	b=12.9319(8)	c=19.0732(10)
	alpha=90	beta=105.739(4)	gamma=90
Temperature:	293 K		

	Calculated	Reported
Volume	2608.4(2)	2608.4(2)
Space group	I 2/a	I 1 2/a 1
Hall group	-I 2ya	-I 2ya
Moiety formula	C16 H4 I4 S4, C7 H8 O	C16 H4 I4 S4, C7 H8 O
Sum formula	C23 H12 I4 O S4	C23 H12 I4 O S4
Mr	940.17	940.17
Dx,g cm-3	2.394	2.394
Z	4	4
Mu (mm-1)	40.647	40.646
F000	1736.0	1736.0
F000'	1742.00	
h,k,lmax	13,15,22	13,15,22
Nref	2395	2392
Tmin,Tmax		0.275,1.000
Tmin'		

Correction method= # Reported T Limits: Tmin=0.275 Tmax=1.000
AbsCorr = EMPIRICAL

Data completeness= 0.999 Theta(max)= 68.204

R(reflections)= 0.0532(2085) wR2(reflections)= 0.1389(2392)

S = 1.059 Npar= 170

The following ALERTS were generated. Each ALERT has the format
test-name_ALERT_alert-type_alert-level.
Click on the hyperlinks for more details of the test.

Alert level B

```
PLAT971_ALERT_2_B Check Calcd Resid. Dens.  0.86Ang From I002          2.88 eA-3
PLAT971_ALERT_2_B Check Calcd Resid. Dens.  0.97Ang From I001          2.52 eA-3
```

Alert level C

```
PLAT053_ALERT_1_C Minimum Crystal Dimension Missing (or Error) ...     Please Check
PLAT054_ALERT_1_C Medium  Crystal Dimension Missing (or Error) ...     Please Check
PLAT055_ALERT_1_C Maximum Crystal Dimension Missing (or Error) ...     Please Check
PLAT342_ALERT_3_C Low Bond Precision on  C-C Bonds ...............    0.00856 Ang.
PLAT906_ALERT_3_C Large K Value in the Analysis of Variance ......      3.721 Check
PLAT971_ALERT_2_C Check Calcd Resid. Dens.  0.81Ang From I002          1.83 eA-3
PLAT971_ALERT_2_C Check Calcd Resid. Dens.  0.91Ang From I001          1.65 eA-3
PLAT971_ALERT_2_C Check Calcd Resid. Dens.  0.96Ang From I002          1.57 eA-3
PLAT971_ALERT_2_C Check Calcd Resid. Dens.  0.87Ang From I001          1.55 eA-3
PLAT972_ALERT_2_C Check Calcd Resid. Dens.  0.85Ang From I001         -1.98 eA-3
PLAT972_ALERT_2_C Check Calcd Resid. Dens.  0.89Ang From I002         -1.96 eA-3
PLAT972_ALERT_2_C Check Calcd Resid. Dens.  0.92Ang From I002         -1.75 eA-3
PLAT972_ALERT_2_C Check Calcd Resid. Dens.  0.96Ang From I001         -1.55 eA-3
PLAT977_ALERT_2_C Check Negative Difference Density on H7C       .      -0.54 eA-3
```

Alert level G

```
PLAT003_ALERT_2_G Number of Uiso or U(i,j)  Restrained non-H-Atoms          9 Report
PLAT083_ALERT_2_G SHELXL Second Parameter in WGHT  Unusually Large       9.82 Why ?
PLAT178_ALERT_4_G The CIF-Embedded .res File Contains SIMU Records          1 Report
PLAT186_ALERT_4_G The CIF-Embedded .res File Contains ISOR Records          1 Report
PLAT188_ALERT_3_G A Non-default SIMU Restraint Value has been used     0.0010 Report
PLAT199_ALERT_1_G Reported _cell_measurement_temperature ..... (K)        293 Check
PLAT200_ALERT_1_G Reported   _diffrn_ambient_temperature ..... (K)        293 Check
PLAT299_ALERT_4_G Atom Site Occupancy Constrained at ............         0.5 Check
              O       C1A     C2      C5      C7      C00F    C00E    C11
              H1A     H2      H00F    H00E    H7A     H7B     H7C     H11
PLAT302_ALERT_4_G Anion/Solvent/Minor-Residue Disorder (Resd    2)       100% Note
PLAT720_ALERT_4_G Number of Unusual/Non-Standard Labels ..........         18 Note
              I001    I002    S003    S004    C005    C006    C007    C008
              C009    C00A    C00B    H00B    C00C    H00C    C00F    H00F
              C00E    H00E
PLAT764_ALERT_4_G Overcomplete CIF Bond List Detected (Rep/Expd) .       1.13 Ratio
PLAT773_ALERT_2_G Check long C-C Bond in CIF: C1A      --C00F            1.88 Ang.
PLAT774_ALERT_1_G Check   X-Y Bond in CIF: O        --C7         ..      5.34 Ang.
PLAT779_ALERT_4_G Suspect or Irrelevant (Bond) Angle(s) in CIF ...      11.00 Deg.
              C5   -O    -C7      1_555   1_555   2_656 ......     #   29 Check
PLAT779_ALERT_4_G Suspect or Irrelevant (Bond) Angle(s) in CIF ...      15.60 Deg.
              C5   -O    -C7      2_656   1_555   2_656 ......     #   31 Check
PLAT779_ALERT_4_G Suspect or Irrelevant (Bond) Angle(s) in CIF ...       5.30 Deg.
              C5   -O    -C5      1_555   1_555   2_656 ......     #   32 Check
PLAT779_ALERT_4_G Suspect or Irrelevant (Bond) Angle(s) in CIF ...      30.10 Deg.
              C2   -C1A  -C00F    1_555   1_555   2_656 ......     #   51 Check
PLAT789_ALERT_4_G Atoms with Negative _atom_site_disorder_group  #         16 Check
```

```
PLAT822_ALERT_4_G CIF-embedded .res Contains Negative PART Numbers          1 Check
PLAT860_ALERT_3_G Number of Least-Squares Restraints .............         54 Note
PLAT883_ALERT_1_G Absent Datum for _atom_sites_solution_primary ..     Please Do !
PLAT912_ALERT_4_G Missing # of FCF Reflections Above STh/L=  0.600          2 Note
PLAT969_ALERT_5_G The 'Henn et al.' R-Factor-gap value ...........      2.399 Note
              Predicted wR2: Based on SigI**2  5.79 or SHELX Weight 13.11
PLAT978_ALERT_2_G Number C-C Bonds with Positive Residual Density.          0 Info
```

```
   0 ALERT level A = Most likely a serious problem - resolve or explain
   2 ALERT level B = A potentially serious problem, consider carefully
  14 ALERT level C = Check. Ensure it is not caused by an omission or oversight
  24 ALERT level G = General information/check it is not something unexpected

   7 ALERT type 1 CIF construction/syntax error, inconsistent or missing data
  15 ALERT type 2 Indicator that the structure model may be wrong or deficient
   4 ALERT type 3 Indicator that the structure quality may be low
  13 ALERT type 4 Improvement, methodology, query or suggestion
   1 ALERT type 5 Informative message, check
```

It is advisable to attempt to resolve as many as possible of the alerts in all categories. Often the minor alerts point to easily fixed oversights, errors and omissions in your CIF or refinement strategy, so attention to these fine details can be worthwhile. In order to resolve some of the more serious problems it may be necessary to carry out additional measurements or structure refinements. However, the purpose of your study may justify the reported deviations and the more serious of these should normally be commented upon in the discussion or experimental section of a paper or in the "special_details" fields of the CIF. checkCIF was carefully designed to identify outliers and unusual parameters, but every test has its limitations and alerts that are not important in a particular case may appear. Conversely, the absence of alerts does not guarantee there are no aspects of the results needing attention. It is up to the individual to critically assess their own results and, if necessary, seek expert advice.

Publication of your CIF in IUCr journals

A basic structural check has been run on your CIF. These basic checks will be run on all CIFs submitted for publication in IUCr journals (*Acta Crystallographica*, *Journal of Applied Crystallography*, *Journal of Synchrotron Radiation*); however, if you intend to submit to *Acta Crystallographica Section C* or *E* or *IUCrData*, you should make sure that full publication checks are run on the final version of your CIF prior to submission.

Publication of your CIF in other journals

Please refer to the *Notes for Authors* of the relevant journal for any special instructions relating to CIF submission.

PLATON version of 04/06/2025; check.def file version of 30/05/2025

Datablock shelx_trans - ellipsoid plot

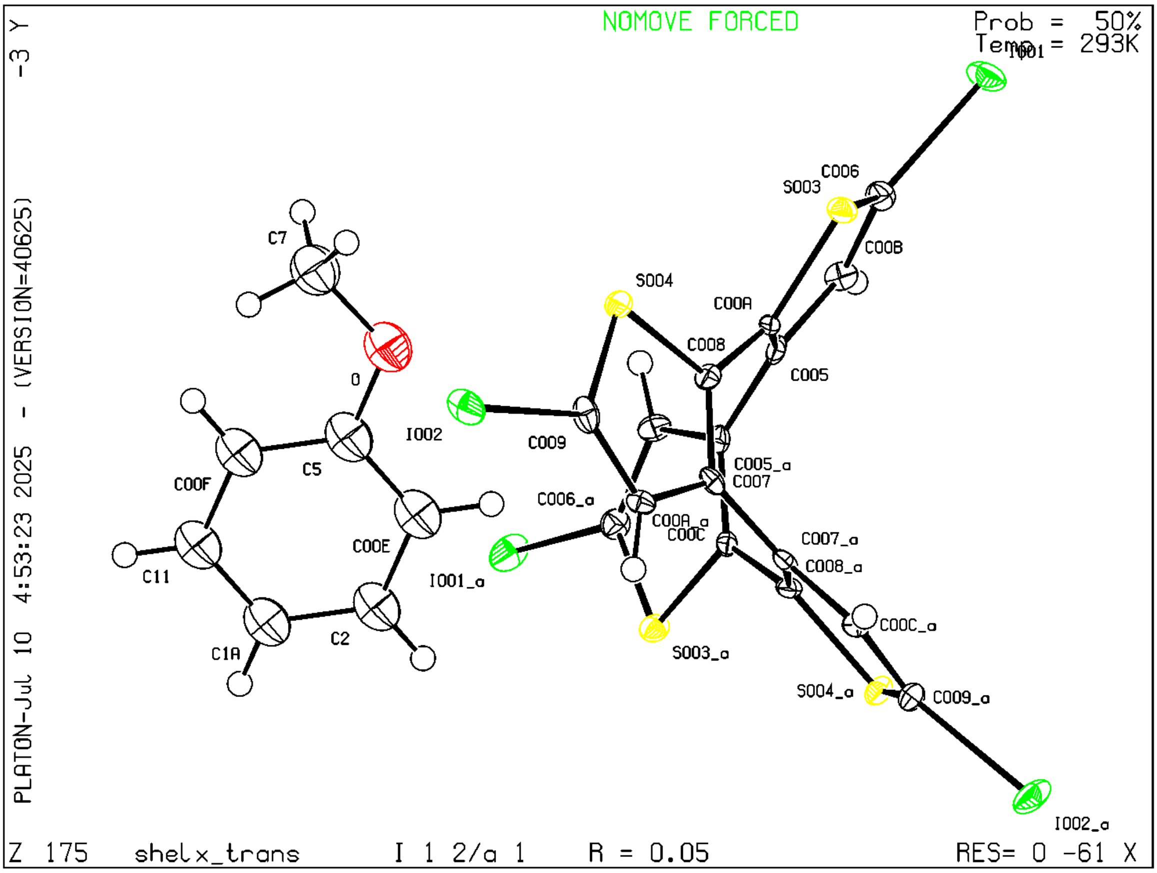

Supplement: Supplementary file 2 — Supporting Information [file CHEM-31-e02872-s001.zip › I_An_100K.pdf]
