# Supplementary material for: Porous Organic Frameworks Utilizing Halogen···Halogen Interactions of X4–tetra[2,3]Thienylene (X = Br, I): Guest Dynamics and Dielectric Response
Source: Chemistry. 2025 Nov 10;31(71):e02872. doi: 10.1002/chem.202502872 (PMC12734654; doi:10.1002/chem.202502872)

checkCIF/PLATON report

Structure factors have been supplied for datablock(s) shelx

THIS REPORT IS FOR GUIDANCE ONLY. IF USED AS PART OF A REVIEW PROCEDURE FOR PUBLICATION, IT SHOULD NOT REPLACE THE EXPERTISE OF AN EXPERIENCED CRYSTALLOGRAPHIC REFEREE.

No syntax errors found. CIF dictionary Interpreting this report

Datablock: shelx

```
Bond precision:   C-C = 0.0160 A          Wavelength=1.54180

Cell:             a=19.2156(8)     b=13.0505(6)        c=10.7236(4)
                  alpha=90         beta=104.995(7)     gamma=90
Temperature:      100 K

                Calculated                    Reported
Volume          2597.6(2)                     2597.6(2)
Space group     P 21/c                        P 1 21/c 1
Hall group      -P 2ybc                       -P 2ybc
Moiety formula  C16 H4 I4 S4, C6 H5 Cl        C16 H4 I4 S4, C6 H5 Cl
Sum formula     C22 H9 Cl I4 S4               C22 H9 Cl I4 S4
Mr              944.58                        944.58
Dx,g cm-3       2.415                         2.415
Z               4                             4
Mu (mm-1)       41.716                        41.716
F000            1736.0                        1736.0
F000'           1743.37
h,k,lmax        23,15,12                      23,15,12
Nref            4749                          4743
Tmin,Tmax       0.005,0.015                   0.310,1.000
Tmin'           0.001

Correction method= # Reported T Limits: Tmin=0.310 Tmax=1.000
AbsCorr = EMPIRICAL

Data completeness= 0.999          Theta(max)= 68.244

                                                         wR2(reflections)=
R(reflections)= 0.0684( 3276)                            0.1735( 4743)

S = 1.093                  Npar= 297
```

The following ALERTS were generated. Each ALERT has the format
test-name_ALERT_alert-type_alert-level.
Click on the hyperlinks for more details of the test.

Alert level C

```
RINTA01_ALERT_3_C  The value of Rint is greater than 0.12
            Rint given   0.144
PLAT213_ALERT_2_C Atom C00G           has ADP max/min Ratio .....        3.2 prolat
PLAT213_ALERT_2_C Atom C00N           has ADP max/min Ratio .....        3.1 oblate
PLAT223_ALERT_4_C Solv./Anion  Resd 2  H   Ueq(max)/Ueq(min) Range       4.3 Ratio
PLAT250_ALERT_2_C Large U3/U1 Ratio for <U(i,j)> Tensor(Resd    1)       2.9 Note
PLAT250_ALERT_2_C Large U3/U1 Ratio for <U(i,j)> Tensor(Resd    2)       2.6 Note
PLAT331_ALERT_2_C Small Aver Phenyl C-C Dist C00P     --C00T     .      1.37 Ang.
PLAT342_ALERT_3_C Low Bond Precision on  C-C Bonds ..............    0.01605 Ang.
PLAT906_ALERT_3_C Large K Value in the Analysis of Variance ......     7.150 Check
PLAT906_ALERT_3_C Large K Value in the Analysis of Variance ......     2.215 Check
PLAT911_ALERT_3_C Missing FCF Refl Between Thmin & STh/L=    0.600         5 Report
              -13  0 12, -13  1 12, -12  0 12, -12  1 12,  -8  0 12,
PLAT934_ALERT_3_C Number of (Iobs-Icalc)/Sigma(W) > 10 Outliers ..         1 Check
              -17  7  8,
PLAT971_ALERT_2_C Check Calcd Resid. Dens.  0.96Ang From I002           2.40 eA-3
PLAT971_ALERT_2_C Check Calcd Resid. Dens.  0.16Ang From I002           2.40 eA-3
PLAT971_ALERT_2_C Check Calcd Resid. Dens.  0.97Ang From I001           2.33 eA-3
PLAT971_ALERT_2_C Check Calcd Resid. Dens.  0.12Ang From I001           2.13 eA-3
PLAT971_ALERT_2_C Check Calcd Resid. Dens.  0.04Ang From I003           2.07 eA-3
PLAT971_ALERT_2_C Check Calcd Resid. Dens.  0.18Ang From I004           1.89 eA-3
PLAT971_ALERT_2_C Check Calcd Resid. Dens.  0.91Ang From I003           1.68 eA-3
PLAT971_ALERT_2_C Check Calcd Resid. Dens.  0.95Ang From I002           1.65 eA-3
PLAT971_ALERT_2_C Check Calcd Resid. Dens.  1.00Ang From I003           1.62 eA-3
PLAT971_ALERT_2_C Check Calcd Resid. Dens.  0.97Ang From I003           1.61 eA-3
PLAT971_ALERT_2_C Check Calcd Resid. Dens.  0.95Ang From I001           1.51 eA-3
PLAT972_ALERT_2_C Check Calcd Resid. Dens.  0.82Ang From I002          -2.27 eA-3
PLAT972_ALERT_2_C Check Calcd Resid. Dens.  0.88Ang From I002          -2.25 eA-3
PLAT972_ALERT_2_C Check Calcd Resid. Dens.  0.85Ang From I003          -2.07 eA-3
PLAT972_ALERT_2_C Check Calcd Resid. Dens.  0.83Ang From I001          -2.07 eA-3
PLAT972_ALERT_2_C Check Calcd Resid. Dens.  0.80Ang From I001          -2.06 eA-3
PLAT972_ALERT_2_C Check Calcd Resid. Dens.  0.80Ang From I004          -1.93 eA-3
PLAT972_ALERT_2_C Check Calcd Resid. Dens.  0.89Ang From I003          -1.88 eA-3
PLAT972_ALERT_2_C Check Calcd Resid. Dens.  0.74Ang From I001          -1.66 eA-3
PLAT972_ALERT_2_C Check Calcd Resid. Dens.  0.85Ang From I004          -1.58 eA-3
PLAT972_ALERT_2_C Check Calcd Resid. Dens.  1.54Ang From I002          -1.58 eA-3
PLAT972_ALERT_2_C Check Calcd Resid. Dens.  0.69Ang From I002          -1.58 eA-3
PLAT977_ALERT_2_C Check Negative Difference Density on H00U      .     -0.32 eA-3
```

Alert level G

```
PLAT002_ALERT_2_G Number of Distance or Angle Restraints on AtSite         4 Note
PLAT003_ALERT_2_G Number of Uiso or U(i,j)  Restrained non-H-Atoms         1 Report
PLAT020_ALERT_3_G The Value of Rint is Greater Than 0.12 .........     0.144 Report
PLAT172_ALERT_4_G The CIF-Embedded .res File Contains DFIX Records         2 Report
PLAT174_ALERT_4_G The CIF-Embedded .res File Contains FLAT Records         1 Report
PLAT186_ALERT_4_G The CIF-Embedded .res File Contains ISOR Records         1 Report
PLAT300_ALERT_4_G Atom Site Occupancy of Cl1        Constrained at      0.78 Check
PLAT300_ALERT_4_G Atom Site Occupancy of Cl2        Constrained at      0.22 Check
PLAT300_ALERT_4_G Atom Site Occupancy of H00P       Constrained at      0.78 Check
```

```
PLAT300_ALERT_4_G Atom Site Occupancy of H00U       Constrained at       0.22 Check
PLAT302_ALERT_4_G Anion/Solvent/Minor-Residue Disorder (Resd    2)        14% Note
PLAT720_ALERT_4_G Number of Unusual/Non-Standard Labels .........          40 Note
              I001    I002    I003    I004    S005    S006    S007    S008
              C00A    C00B    C00C    C00D    C00E    H00E    C00F    H00F
              C00G    H00G    C00H    C00I    C00J    H00J    C00K    C00L
              C00M    C00N    C00O    C00P    C00Q    H00Q    C00R    C00S
              H00S    C00T    H00T    C00U    C00V    H00V    H00U    H00P
PLAT860_ALERT_3_G Number of Least-Squares Restraints ............          11 Note
PLAT883_ALERT_1_G Absent Datum for _atom_sites_solution_primary ..     Please Do !
PLAT910_ALERT_3_G Missing FCF Reflection(s) Below Theta(Min)[Deg]=       4.14 Note
                1  0  0,
PLAT969_ALERT_5_G The 'Henn et al.' R-Factor-gap value ..........      1.741 Note
              Predicted wR2: Based on SigI**2  9.97 or SHELX Weight 15.87
PLAT978_ALERT_2_G Number C-C Bonds with Positive Residual Density.          0 Info
```

---

  0 **ALERT level A** = Most likely a serious problem - resolve or explain
  0 **ALERT level B** = A potentially serious problem, consider carefully
 35 **ALERT level C** = Check. Ensure it is not caused by an omission or oversight
 17 **ALERT level G** = General information/check it is not something unexpected

  1 ALERT type 1 CIF construction/syntax error, inconsistent or missing data
 31 ALERT type 2 Indicator that the structure model may be wrong or deficient
  9 ALERT type 3 Indicator that the structure quality may be low
 10 ALERT type 4 Improvement, methodology, query or suggestion
  1 ALERT type 5 Informative message, check

It is advisable to attempt to resolve as many as possible of the alerts in all categories. Often the minor alerts point to easily fixed oversights, errors and omissions in your CIF or refinement strategy, so attention to these fine details can be worthwhile. In order to resolve some of the more serious problems it may be necessary to carry out additional measurements or structure refinements. However, the purpose of your study may justify the reported deviations and the more serious of these should normally be commented upon in the discussion or experimental section of a paper or in the "special_details" fields of the CIF. checkCIF was carefully designed to identify outliers and unusual parameters, but every test has its limitations and alerts that are not important in a particular case may appear. Conversely, the absence of alerts does not guarantee there are no aspects of the results needing attention. It is up to the individual to critically assess their own results and, if necessary, seek expert advice.

Publication of your CIF in IUCr journals

A basic structural check has been run on your CIF. These basic checks will be run on all CIFs submitted for publication in IUCr journals (*Acta Crystallographica*, *Journal of Applied Crystallography*, *Journal of Synchrotron Radiation*); however, if you intend to submit to *Acta Crystallographica Section C* or *E* or *IUCrData*, you should make sure that full publication checks are run on the final version of your CIF prior to submission.

Publication of your CIF in other journals

Please refer to the *Notes for Authors* of the relevant journal for any special instructions relating to CIF submission.

PLATON version of 04/06/2025; check.def file version of 30/05/2025

Datablock shelx - ellipsoid plot

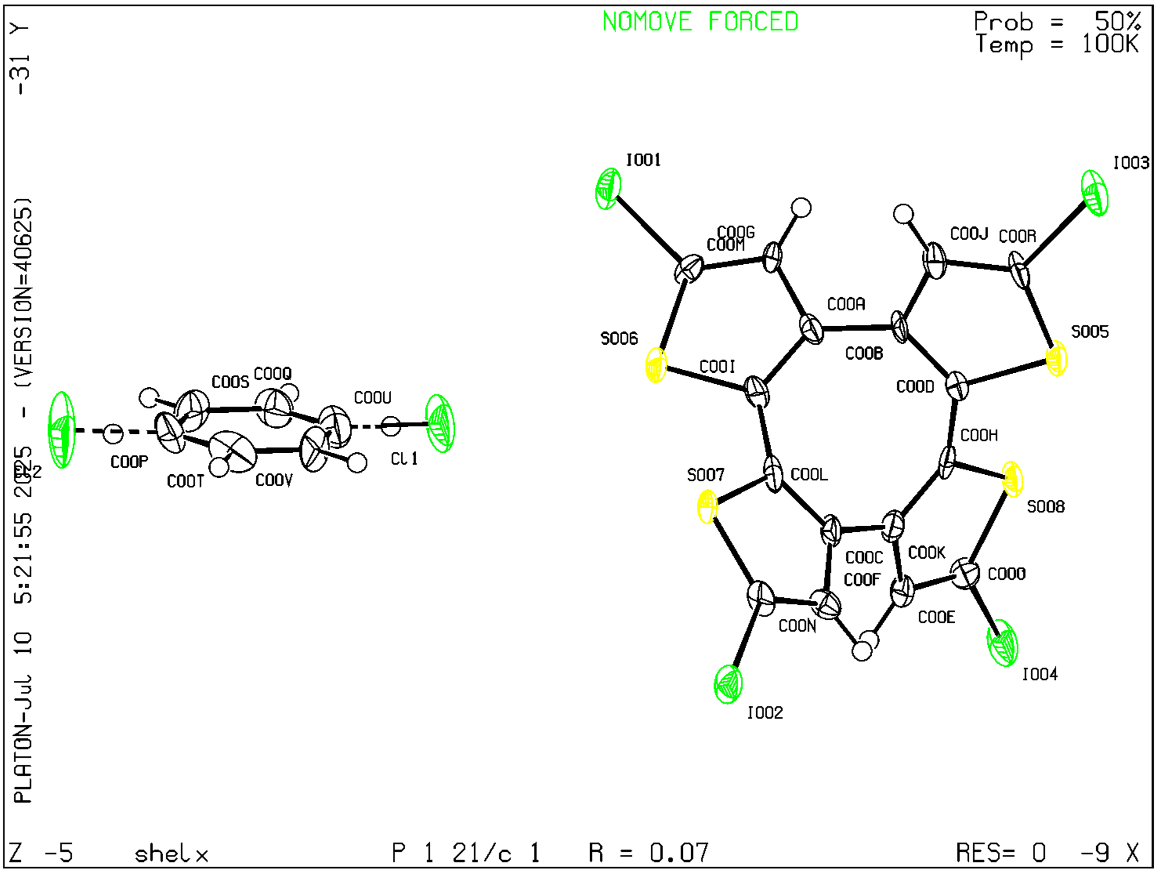

Supplement: Supplementary file 2 — Supporting Information [file CHEM-31-e02872-s001.zip › I_ClBz_100K.pdf]
