# Supplementary material for: Porous Organic Frameworks Utilizing Halogen···Halogen Interactions of X4–tetra[2,3]Thienylene (X = Br, I): Guest Dynamics and Dielectric Response
Source: Chemistry. 2025 Nov 10;31(71):e02872. doi: 10.1002/chem.202502872 (PMC12734654; doi:10.1002/chem.202502872)

checkCIF/PLATON report

Structure factors have been supplied for datablock(s) shelx_trans

THIS REPORT IS FOR GUIDANCE ONLY. IF USED AS PART OF A REVIEW PROCEDURE FOR PUBLICATION, IT SHOULD NOT REPLACE THE EXPERTISE OF AN EXPERIENCED CRYSTALLOGRAPHIC REFEREE.

No syntax errors found.   CIF dictionary   Interpreting this report

Datablock: shelx_trans

Bond precision:	C-C = 0.0107 A		Wavelength=1.54180
Cell:	a=11.0401(4)	b=13.1470(5)	c=19.1636(15)
	alpha=90	beta=105.497(6)	gamma=90
Temperature:	293 K		

	Calculated	Reported
Volume	2680.4(3)	2680.4(3)
Space group	I 2/a	I 1 2/a 1
Hall group	-I 2ya	-I 2ya
Moiety formula	C16 H4 I4 S4, C6 H5 Cl	C16 H4 I4 S4, C6 H5 Cl
Sum formula	C22 H9 Cl I4 S4	C22 H9 Cl I4 S4
Mr	944.58	944.58
Dx,g cm-3	2.341	2.341
Z	4	4
Mu (mm-1)	40.427	40.428
F000	1736.0	1736.0
F000'	1743.37	
h,k,lmax	13,15,23	13,15,23
Nref	2454	2452
Tmin,Tmax		0.132,1.000
Tmin'		

Correction method= # Reported T Limits: Tmin=0.132 Tmax=1.000
AbsCorr = EMPIRICAL

Data completeness= 0.999   Theta(max)= 68.206

R(reflections)= 0.0534(1976)   wR2(reflections)= 0.1430(2452)

S = 1.023   Npar= 149

The following ALERTS were generated. Each ALERT has the format
test-name_ALERT_alert-type_alert-level.
Click on the hyperlinks for more details of the test.

Alert level C

PLAT053_ALERT_1_C Minimum Crystal Dimension Missing (or Error) ... Please Check
PLAT054_ALERT_1_C Medium Crystal Dimension Missing (or Error) ... Please Check
PLAT055_ALERT_1_C Maximum Crystal Dimension Missing (or Error) ... Please Check
PLAT234_ALERT_4_C Large Hirshfeld Difference C00F --C00G . 0.17 Ang.
PLAT244_ALERT_4_C Low 'Solvent' Ueq as Compared to Neighbors of C00F Check
PLAT260_ALERT_2_C Large Average Ueq of Residue Including Cl00 0.119 Check
PLAT342_ALERT_3_C Low Bond Precision on C-C Bonds .... 0.01067 Ang.
PLAT906_ALERT_3_C Large K Value in the Analysis of Variance ...... 4.625 Check
PLAT972_ALERT_2_C Check Calcd Resid. Dens. 0.86Ang From I001 -1.51 eA-3

Alert level G

PLAT002_ALERT_2_G Number of Distance or Angle Restraints on AtSite 5 Note
PLAT172_ALERT_4_G The CIF-Embedded .res File Contains DFIX Records 2 Report
PLAT173_ALERT_4_G The CIF-Embedded .res File Contains DANG Records 1 Report
PLAT174_ALERT_4_G The CIF-Embedded .res File Contains FLAT Records 1 Report
PLAT199_ALERT_1_G Reported _cell_measurement_temperature ..... (K) 293 Check
PLAT200_ALERT_1_G Reported _diffrn_ambient_temperature ..... (K) 293 Check
PLAT299_ALERT_4_G Atom Site Occupancy Constrained at .... 0.5 Check
Cl00 H00F
PLAT302_ALERT_4_G Anion/Solvent/Minor-Residue Disorder (Resd 2) 14% Note
PLAT720_ALERT_4_G Number of Unusual/Non-Standard Labels .... 21 Note
I001 I002 S003 S004 C005 C006 C007 C008
C009 H009 C00A H00A C00B C00C Cl00 C00E
H00E C00F C00G H00G H00F
PLAT860_ALERT_3_G Number of Least-Squares Restraints .... 6 Note
PLAT912_ALERT_4_G Missing # of FCF Reflections Above STh/L= 0.600 1 Note
PLAT969_ALERT_5_G The 'Henn et al.' R-Factor-gap value .... 2.137 Note
Predicted wR2: Based on SigI**2 6.69 or SHELX Weight 13.98
PLAT978_ALERT_2_G Number C-C Bonds with Positive Residual Density. 0 Info

0 **ALERT level A** = Most likely a serious problem - resolve or explain
0 **ALERT level B** = A potentially serious problem, consider carefully
9 **ALERT level C** = Check. Ensure it is not caused by an omission or oversight
13 **ALERT level G** = General information/check it is not something unexpected

5 ALERT type 1 CIF construction/syntax error, inconsistent or missing data
4 ALERT type 2 Indicator that the structure model may be wrong or deficient
3 ALERT type 3 Indicator that the structure quality may be low
9 ALERT type 4 Improvement, methodology, query or suggestion
1 ALERT type 5 Informative message, check

It is advisable to attempt to resolve as many as possible of the alerts in all categories. Often the minor alerts point to easily fixed oversights, errors and omissions in your CIF or refinement strategy, so attention to these fine details can be worthwhile. In order to resolve some of the more serious problems it may be necessary to carry out additional measurements or structure refinements. However, the purpose of your study may justify the reported deviations and the more serious of these should normally be commented upon in the discussion or experimental section of a paper or in the "special_details" fields of the CIF. checkCIF was carefully designed to identify outliers and unusual parameters, but every test has its limitations and alerts that are not important in a particular case may appear. Conversely, the absence of alerts does not guarantee there are no aspects of the results needing attention. It is up to the individual to critically assess their own results and, if necessary, seek expert advice.

Publication of your CIF in IUCr journals

A basic structural check has been run on your CIF. These basic checks will be run on all CIFs submitted for publication in IUCr journals (*Acta Crystallographica*, *Journal of Applied Crystallography*, *Journal of Synchrotron Radiation*); however, if you intend to submit to *Acta Crystallographica Section C* or *E* or *IUCrData*, you should make sure that full publication checks are run on the final version of your CIF prior to submission.

Publication of your CIF in other journals

Please refer to the *Notes for Authors* of the relevant journal for any special instructions relating to CIF submission.

PLATON version of 04/06/2025; check.def file version of 30/05/2025

Datablock shelx_trans - ellipsoid plot

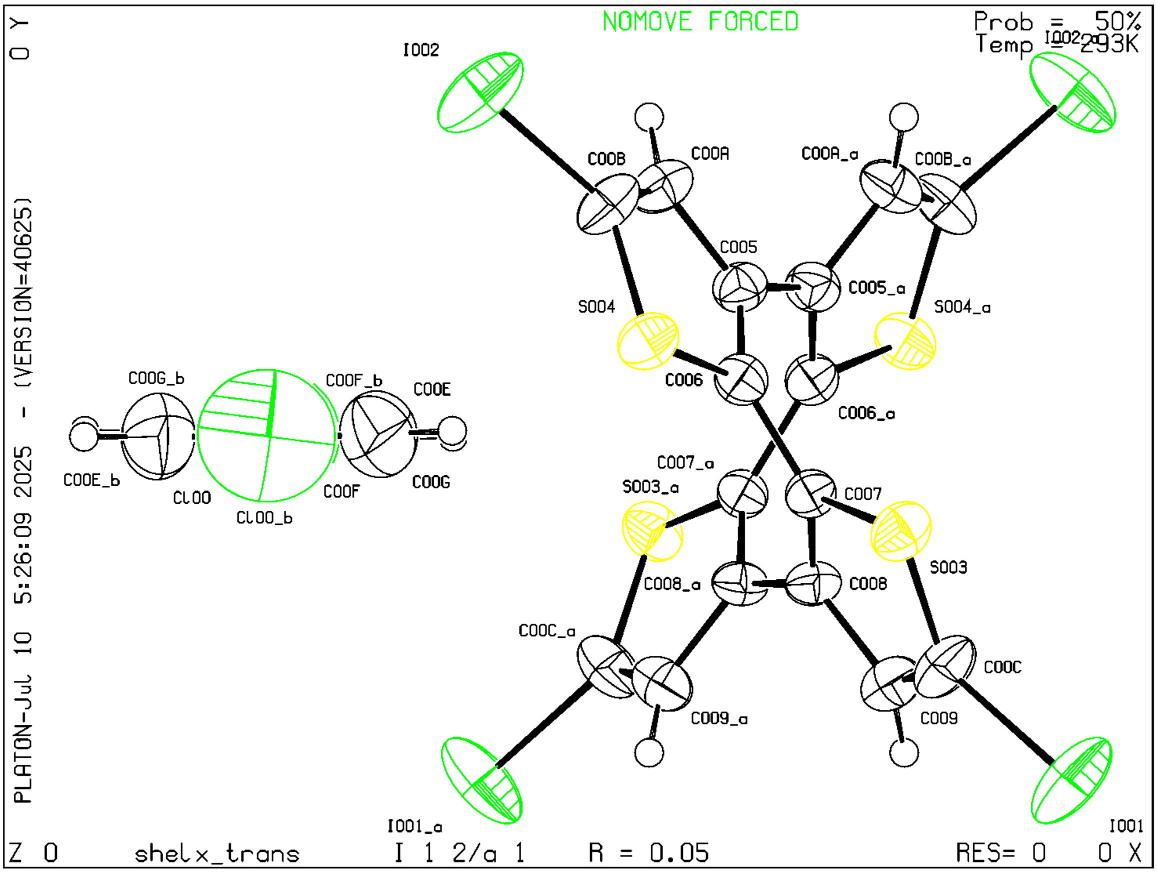

Supplement: Supplementary file 2 — Supporting Information [file CHEM-31-e02872-s001.zip › I_ClBz_293K.pdf]
