# Supplementary material for: Porous Organic Frameworks Utilizing Halogen···Halogen Interactions of X4–tetra[2,3]Thienylene (X = Br, I): Guest Dynamics and Dielectric Response
Source: Chemistry. 2025 Nov 10;31(71):e02872. doi: 10.1002/chem.202502872 (PMC12734654; doi:10.1002/chem.202502872)

# checkCIF/PLATON report

Structure factors have been supplied for datablock(s) shelx

THIS REPORT IS FOR GUIDANCE ONLY. IF USED AS PART OF A REVIEW PROCEDURE FOR PUBLICATION, IT SHOULD NOT REPLACE THE EXPERTISE OF AN EXPERIENCED CRYSTALLOGRAPHIC REFEREE.

No syntax errors found. CIF dictionary Interpreting this report

# Datablock: shelx

---

| | | | |
|---|---|---|---|
| Bond precision: | C-C = 0.0174 A | | Wavelength=1.54180 |
| Cell: | a=8.1735(6) | b=17.3132(13) | c=14.9519(9) |
| | alpha=90 | beta=90 | gamma=90 |
| Temperature: | 100 K | | |

| | Calculated | Reported |
|---|---|---|
| Volume | 2115.8(3) | 2115.8(3) |
| Space group | P n m a | P n m a |
| Hall group | -P 2ac 2n | -P 2ac 2n |
| Moiety formula | C16 H4 I4 S4 | C16 H4 I4 S4 |
| Sum formula | C16 H4 I4 S4 | C16 H4 I4 S4 |
| Mr | 832.03 | 832.03 |
| Dx,g cm-3 | 2.612 | 2.612 |
| Z | 4 | 4 |
| Mu (mm-1) | 49.933 | 49.932 |
| F000 | 1504.0 | 1504.0 |
| F000' | 1509.92 | |
| h,k,lmax | 9,20,18 | 9,20,17 |
| Nref | 2002 | 2001 |
| Tmin,Tmax | 0.006,0.007 | 0.300,1.000 |
| Tmin' | 0.000 | |

Correction method= # Reported T Limits: Tmin=0.300 Tmax=1.000
AbsCorr = EMPIRICAL

```
Data completeness= 1.000          Theta(max)= 68.219

                                                      wR2(reflections)=
R(reflections)= 0.0698( 1040)                         0.1833( 2001)

S = 0.973                 Npar= 169
```

---

```
The following ALERTS were generated. Each ALERT has the format
       test-name_ALERT_alert-type_alert-level.
Click on the hyperlinks for more details of the test.
```

---

**Alert level B**

```
RINTA01_ALERT_3_B  The value of Rint is greater than 0.18
            Rint given   0.183
```

---

**Alert level C**

```
PLAT234_ALERT_4_C Large Hirshfeld Difference I001     --C009     .       0.18 Ang.
PLAT234_ALERT_4_C Large Hirshfeld Difference S006     --C00D     .       0.18 Ang.
PLAT342_ALERT_3_C Low Bond Precision on  C-C Bonds ..............     0.0174 Ang.
PLAT906_ALERT_3_C Large K Value in the Analysis of Variance ......     16.632 Check
PLAT906_ALERT_3_C Large K Value in the Analysis of Variance ......      6.142 Check
PLAT906_ALERT_3_C Large K Value in the Analysis of Variance ......      2.244 Check
```

---

**Alert level G**

```
PLAT002_ALERT_2_G Number of Distance or Angle Restraints on AtSite         14 Note
PLAT003_ALERT_2_G Number of Uiso or U(i,j)  Restrained non-H-Atoms          7 Report
PLAT020_ALERT_3_G The Value of Rint is Greater Than 0.12 .........      0.183 Report
PLAT172_ALERT_4_G The CIF-Embedded .res File Contains DFIX Records          4 Report
PLAT178_ALERT_4_G The CIF-Embedded .res File Contains SIMU Records          1 Report
PLAT186_ALERT_4_G The CIF-Embedded .res File Contains ISOR Records          1 Report
PLAT188_ALERT_3_G A Non-default SIMU Restraint Value has been used     0.0100 Report
PLAT299_ALERT_4_G Atom Site Occupancy Constrained at ............         0.5 Check
              I003    I004    S005    S006    S007    S0      C1      C008
              C2      C3      C3A     C4      H1      H008    H2      H4
PLAT301_ALERT_3_G Main Residue  Disorder .............(Resd    1)        50% Note
PLAT720_ALERT_4_G Number of Unusual/Non-Standard Labels .........         16 Note
              I001    I002    I003    I004    S005    S006    S007    C008
              H008    C009    C00A    C00B    C00C    C00D    C00E    S0
PLAT764_ALERT_4_G Overcomplete CIF Bond List Detected (Rep/Expd) .       1.16 Ratio
PLAT789_ALERT_4_G Atoms with Negative _atom_site_disorder_group  #         10 Check
PLAT811_ALERT_5_G No ADDSYM Analysis: Too Many Excluded Atoms ....          ! Info
PLAT822_ALERT_4_G CIF-embedded .res Contains Negative PART Numbers          7 Check
PLAT860_ALERT_3_G Number of Least-Squares Restraints ............         46 Note
PLAT883_ALERT_1_G Absent Datum for _atom_sites_solution_primary ..     Please Do !
PLAT969_ALERT_5_G The 'Henn et al.' R-Factor-gap value ..........      2.092 Note
              Predicted wR2: Based on SigI**2  8.76 or SHELX Weight 18.85
PLAT978_ALERT_2_G Number C-C Bonds with Positive Residual Density.          0 Info
```

---

0 **ALERT level A** = Most likely a serious problem - resolve or explain
1 **ALERT level B** = A potentially serious problem, consider carefully
6 **ALERT level C** = Check. Ensure it is not caused by an omission or oversight

```
18 ALERT level G = General information/check it is not something unexpected

 1 ALERT type 1 CIF construction/syntax error, inconsistent or missing data
 3 ALERT type 2 Indicator that the structure model may be wrong or deficient
 9 ALERT type 3 Indicator that the structure quality may be low
10 ALERT type 4 Improvement, methodology, query or suggestion
 2 ALERT type 5 Informative message, check
```

---

---

It is advisable to attempt to resolve as many as possible of the alerts in all categories. Often the minor alerts point to easily fixed oversights, errors and omissions in your CIF or refinement strategy, so attention to these fine details can be worthwhile. It is up to the individual to critically assess their own results and, if necessary, seek expert advice.

---

## PLATON version of 04/06/2025; check.def file version of 30/05/2025

**Datablock shelx** - ellipsoid plot

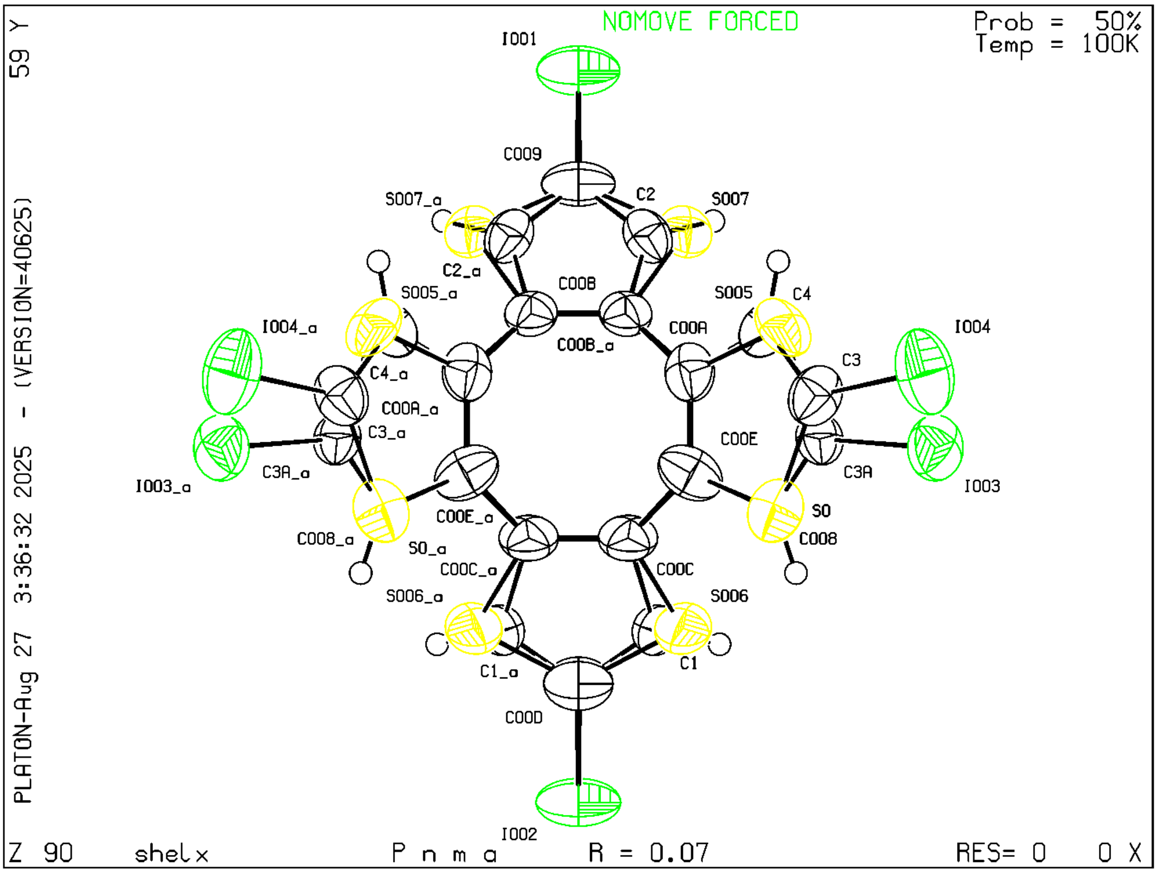

Supplement: Supplementary file 2 — Supporting Information [file CHEM-31-e02872-s001.zip › I_host_100K.pdf]
