# Supplementary material for: Porous Organic Frameworks Utilizing Halogen···Halogen Interactions of X4–tetra[2,3]Thienylene (X = Br, I): Guest Dynamics and Dielectric Response
Source: Chemistry. 2025 Nov 10;31(71):e02872. doi: 10.1002/chem.202502872 (PMC12734654; doi:10.1002/chem.202502872)

# checkCIF/PLATON report

Structure factors have been supplied for datablock(s) shelx_trans

THIS REPORT IS FOR GUIDANCE ONLY. IF USED AS PART OF A REVIEW PROCEDURE FOR PUBLICATION, IT SHOULD NOT REPLACE THE EXPERTISE OF AN EXPERIENCED CRYSTALLOGRAPHIC REFEREE.

No syntax errors found. CIF dictionary Interpreting this report

## Datablock: shelx_trans

---

```
Bond precision:   C-C = 0.0098 A          Wavelength=1.54180

Cell:             a=10.9151(3)    b=13.0111(4)       c=19.1733(14)
                  alpha=90        beta=105.147(6)    gamma=90
Temperature:      100 K

                 Calculated                   Reported
Volume           2628.3(2)                    2628.3(2)
Space group      I 2/a                        I 1 2/a 1
Hall group       -I 2ya                       -I 2ya
Moiety formula   C16 H4 I4 S4, C7 H8          C16 H4 I4 S4, C7 H8
Sum formula      C23 H12 I4 S4                C23 H12 I4 S4
Mr               924.17                       924.17
Dx,g cm-3        2.336                        2.335
Z                4                            4
Mu (mm-1)        40.293                       40.292
F000             1704.0                       1704.0
F000'            1709.87
h,k,lmax         13,15,23                     13,15,23
Nref             2412                         2412
Tmin,Tmax                                     0.191,1.000
Tmin'

Correction method= # Reported T Limits: Tmin=0.191 Tmax=1.000
AbsCorr = EMPIRICAL

Data completeness= 1.000          Theta(max)= 68.244

                                                    wR2(reflections)=
R(reflections)= 0.0626( 2046)                       0.1609( 2412)

S = 1.054                  Npar= 150
```

The following ALERTS were generated. Each ALERT has the format
**test-name_ALERT_alert-type_alert-level.**
Click on the hyperlinks for more details of the test.

### Alert level B

```
PLAT971_ALERT_2_B Check Calcd Resid. Dens.  1.05Ang From I001          3.23 eA-3
PLAT971_ALERT_2_B Check Calcd Resid. Dens.  1.10Ang From I002          3.16 eA-3
PLAT971_ALERT_2_B Check Calcd Resid. Dens.  0.90Ang From I002          2.98 eA-3
PLAT971_ALERT_2_B Check Calcd Resid. Dens.  0.91Ang From I001          2.70 eA-3
```

### Alert level C

```
RINTA01_ALERT_3_C  The value of Rint is greater than 0.12
          Rint given   0.134
PLAT053_ALERT_1_C Minimum Crystal Dimension Missing (or Error) ...     Please Check
PLAT054_ALERT_1_C Medium  Crystal Dimension Missing (or Error) ...     Please Check
PLAT055_ALERT_1_C Maximum Crystal Dimension Missing (or Error) ...     Please Check
PLAT213_ALERT_2_C Atom C00C            has ADP max/min Ratio .....        3.3 prolat
PLAT250_ALERT_2_C Large U3/U1 Ratio for <U(i,j)> Tensor(Resd    1)        2.7 Note
PLAT342_ALERT_3_C Low Bond Precision on  C-C Bonds ..............    0.00975 Ang.
PLAT971_ALERT_2_C Check Calcd Resid. Dens.  0.92Ang From I001          2.05 eA-3
PLAT971_ALERT_2_C Check Calcd Resid. Dens.  0.92Ang From I002          1.80 eA-3
PLAT972_ALERT_2_C Check Calcd Resid. Dens.  0.88Ang From I001         -1.77 eA-3
PLAT972_ALERT_2_C Check Calcd Resid. Dens.  0.99Ang From I002         -1.58 eA-3
```

### Alert level G

```
PLAT002_ALERT_2_G Number of Distance or Angle Restraints on AtSite          2 Note
PLAT020_ALERT_3_G The Value of Rint is Greater Than 0.12 .........      0.134 Report
PLAT072_ALERT_2_G SHELXL First  Parameter in WGHT  Unusually Large       0.10 Report
PLAT172_ALERT_4_G The CIF-Embedded .res File Contains DFIX Records          1 Report
PLAT174_ALERT_4_G The CIF-Embedded .res File Contains FLAT Records          1 Report
PLAT299_ALERT_4_G Atom Site Occupancy Constrained at ............         0.5 Check
              C5      H00D    H5A     H5B     H5C
PLAT302_ALERT_4_G Anion/Solvent/Minor-Residue Disorder (Resd    2)        14% Note
PLAT720_ALERT_4_G Number of Unusual/Non-Standard Labels ..........         20 Note
              I001    I002    S003    S004    C005    C006    C007    H007
              C008    H008    C009    C00A    C00B    C00C    C00D    C00E
              H00E    C00F    H00F    H00D
PLAT860_ALERT_3_G Number of Least-Squares Restraints ............           2 Note
PLAT969_ALERT_5_G The 'Henn et al.' R-Factor-gap value ..........       2.033 Note
              Predicted wR2: Based on SigI**2  7.91 or SHELX Weight 15.27
PLAT978_ALERT_2_G Number C-C Bonds with Positive Residual Density.          2 Info
```

0 **ALERT level A** = Most likely a serious problem - resolve or explain
4 **ALERT level B** = A potentially serious problem, consider carefully
11 **ALERT level C** = Check. Ensure it is not caused by an omission or oversight
11 **ALERT level G** = General information/check it is not something unexpected

3 ALERT type 1 CIF construction/syntax error, inconsistent or missing data

```
13 ALERT type 2 Indicator that the structure model may be wrong or deficient
 4 ALERT type 3 Indicator that the structure quality may be low
 5 ALERT type 4 Improvement, methodology, query or suggestion
 1 ALERT type 5 Informative message, check
```

---

It is advisable to attempt to resolve as many as possible of the alerts in all categories. Often the minor alerts point to easily fixed oversights, errors and omissions in your CIF or refinement strategy, so attention to these fine details can be worthwhile. In order to resolve some of the more serious problems it may be necessary to carry out additional measurements or structure refinements. However, the purpose of your study may justify the reported deviations and the more serious of these should normally be commented upon in the discussion or experimental section of a paper or in the "special_details" fields of the CIF. checkCIF was carefully designed to identify outliers and unusual parameters, but every test has its limitations and alerts that are not important in a particular case may appear. Conversely, the absence of alerts does not guarantee there are no aspects of the results needing attention. It is up to the individual to critically assess their own results and, if necessary, seek expert advice.

**Publication of your CIF in IUCr journals**

A basic structural check has been run on your CIF. These basic checks will be run on all CIFs submitted for publication in IUCr journals (*Acta Crystallographica*, *Journal of Applied Crystallography*, *Journal of Synchrotron Radiation*); however, if you intend to submit to *Acta Crystallographica Section C* or *E* or *IUCrData*, you should make sure that full publication checks are run on the final version of your CIF prior to submission.

**Publication of your CIF in other journals**

Please refer to the *Notes for Authors* of the relevant journal for any special instructions relating to CIF submission.

---

**PLATON version of 04/06/2025; check.def file version of 30/05/2025**

**Datablock shelx_trans** - ellipsoid plot

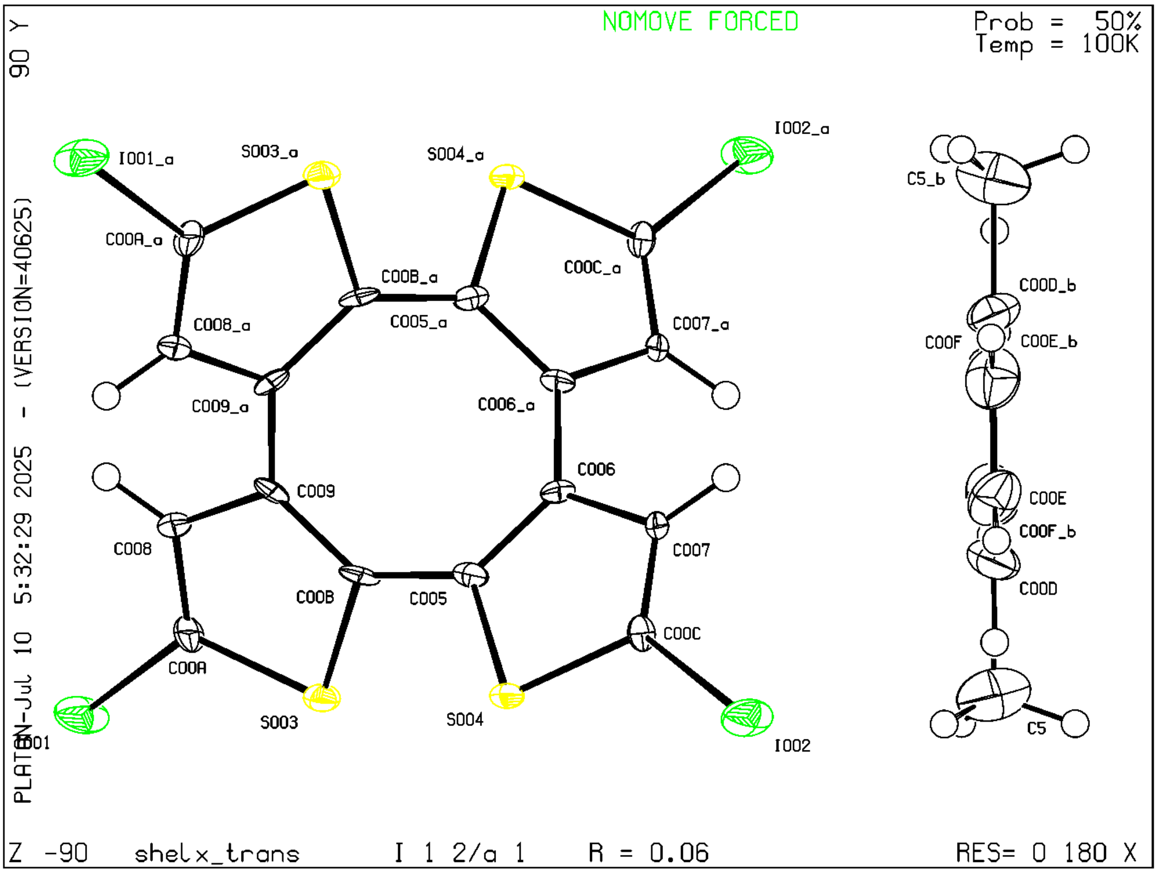

Supplement: Supplementary file 2 — Supporting Information [file CHEM-31-e02872-s001.zip › I_Tol_100K.pdf]
