# Supplementary material for: Porous Organic Frameworks Utilizing Halogen···Halogen Interactions of X4–tetra[2,3]Thienylene (X = Br, I): Guest Dynamics and Dielectric Response
Source: Chemistry. 2025 Nov 10;31(71):e02872. doi: 10.1002/chem.202502872 (PMC12734654; doi:10.1002/chem.202502872)

checkCIF/PLATON report

Structure factors have been supplied for datablock(s) shelx_trans

THIS REPORT IS FOR GUIDANCE ONLY. IF USED AS PART OF A REVIEW PROCEDURE FOR PUBLICATION, IT SHOULD NOT REPLACE THE EXPERTISE OF AN EXPERIENCED CRYSTALLOGRAPHIC REFEREE.

No syntax errors found. CIF dictionary Interpreting this report

Datablock: shelx_trans

Bond precision: C-C = 0.0066 A Wavelength=1.54180

Cell:	a=7.8916(2)	b=16.5739(4)	c=14.4346(3)
	alpha=90	beta=90	gamma=90
Temperature:	100 K		

	Calculated	Reported
Volume	1887.97(8)	1887.97(8)
Space group	P n m a	P n m a
Hall group	-P 2ac 2n	-P 2ac 2n
Moiety formula	C16 H4 Br4 S4	C16 H4 Br4 S4
Sum formula	C16 H4 Br4 S4	C16 H4 Br4 S4
Mr	644.03	644.07
Dx,g cm-3	2.266	2.266
Z	4	4
Mu (mm-1)	14.517	14.517
F000	1216.0	1216.0
F000’	1211.87	
h,k,lmax	9,19,17	9,19,17
Nref	1794	1794
Tmin,Tmax		0.553,1.000
Tmin’		

Correction method= # Reported T Limits: Tmin=0.553 Tmax=1.000
AbsCorr = EMPIRICAL

Data completeness= 1.000 Theta(max)= 68.232

R(reflections)= 0.0440(1631) wR2(reflections)= 0.0949(1794)

S = 1.135 Npar= 169

The following ALERTS were generated. Each ALERT has the format
test-name_ALERT_alert-type_alert-level.
Click on the hyperlinks for more details of the test.

Alert level C

```
PLAT053_ALERT_1_C Minimum Crystal Dimension Missing (or Error) ...     Please Check
PLAT054_ALERT_1_C Medium  Crystal Dimension Missing (or Error) ...     Please Check
PLAT055_ALERT_1_C Maximum Crystal Dimension Missing (or Error) ...     Please Check
PLAT234_ALERT_4_C Large Hirshfeld Difference C1       --C00A     .       0.16 Ang.
PLAT234_ALERT_4_C Large Hirshfeld Difference C3       --C00C     .       0.18 Ang.
PLAT341_ALERT_3_C Low Bond Precision on  C-C Bonds ..............     0.0066 Ang.
PLAT906_ALERT_3_C Large K Value in the Analysis of Variance ......      7.058 Check
```

Alert level G

```
PLAT002_ALERT_2_G Number of Distance or Angle Restraints on AtSite          8 Note
PLAT003_ALERT_2_G Number of Uiso or U(i,j)  Restrained non-H-Atoms          5 Report
PLAT083_ALERT_2_G SHELXL Second Parameter in WGHT  Unusually Large       7.16 Why ?
PLAT172_ALERT_4_G The CIF-Embedded .res File Contains DFIX Records          2 Report
PLAT178_ALERT_4_G The CIF-Embedded .res File Contains SIMU Records          1 Report
PLAT186_ALERT_4_G The CIF-Embedded .res File Contains ISOR Records          1 Report
PLAT188_ALERT_3_G A Non-default SIMU Restraint Value has been used     0.0100 Report
PLAT299_ALERT_4_G Atom Site Occupancy Constrained at ............         0.5 Check
              Br      Br1     S005    S006    S007    S008    C1      C2
              C3      C00F    C0      C00G    H1      H0      H2      H3
PLAT301_ALERT_3_G Main Residue  Disorder .............(Resd    1)         50% Note
PLAT720_ALERT_4_G Number of Unusual/Non-Standard Labels ..........         16 Note
              Br01    Br02    S005    S006    S007    S008    C009    C00A
              C00B    C00C    C00D    C00E    C00F    C0      H0      C00G
PLAT764_ALERT_4_G Overcomplete CIF Bond List Detected (Rep/Expd) .       1.16 Ratio
PLAT789_ALERT_4_G Atoms with Negative _atom_site_disorder_group  #         10 Check
PLAT811_ALERT_5_G No ADDSYM Analysis: Too Many Excluded Atoms ....          ! Info
PLAT822_ALERT_4_G CIF-embedded .res Contains Negative PART Numbers          5 Check
PLAT860_ALERT_3_G Number of Least-Squares Restraints ............          35 Note
PLAT883_ALERT_1_G Absent Datum for _atom_sites_solution_primary ..     Please Do !
PLAT969_ALERT_5_G The 'Henn et al.' R-Factor-gap value ..........      3.384 Note
              Predicted wR2: Based on SigI**2  2.80 or SHELX Weight  8.36
PLAT978_ALERT_2_G Number C-C Bonds with Positive Residual Density.          2 Info
```

0 **ALERT level A** = Most likely a serious problem - resolve or explain
0 **ALERT level B** = A potentially serious problem, consider carefully
7 **ALERT level C** = Check. Ensure it is not caused by an omission or oversight
18 **ALERT level G** = General information/check it is not something unexpected

4 ALERT type 1 CIF construction/syntax error, inconsistent or missing data
4 ALERT type 2 Indicator that the structure model may be wrong or deficient
5 ALERT type 3 Indicator that the structure quality may be low
10 ALERT type 4 Improvement, methodology, query or suggestion
2 ALERT type 5 Informative message, check

It is advisable to attempt to resolve as many as possible of the alerts in all categories. Often the minor alerts point to easily fixed oversights, errors and omissions in your CIF or refinement strategy, so attention to these fine details can be worthwhile. In order to resolve some of the more serious problems it may be necessary to carry out additional measurements or structure refinements. However, the purpose of your study may justify the reported deviations and the more serious of these should normally be commented upon in the discussion or experimental section of a paper or in the "special_details" fields of the CIF. checkCIF was carefully designed to identify outliers and unusual parameters, but every test has its limitations and alerts that are not important in a particular case may appear. Conversely, the absence of alerts does not guarantee there are no aspects of the results needing attention. It is up to the individual to critically assess their own results and, if necessary, seek expert advice.

Publication of your CIF in IUCr journals

A basic structural check has been run on your CIF. These basic checks will be run on all CIFs submitted for publication in IUCr journals (*Acta Crystallographica*, *Journal of Applied Crystallography*, *Journal of Synchrotron Radiation*); however, if you intend to submit to *Acta Crystallographica Section C* or *E* or *IUCrData*, you should make sure that full publication checks are run on the final version of your CIF prior to submission.

Publication of your CIF in other journals

Please refer to the *Notes for Authors* of the relevant journal for any special instructions relating to CIF submission.

PLATON version of 04/06/2025; check.def file version of 30/05/2025

Datablock shelx_trans - ellipsoid plot

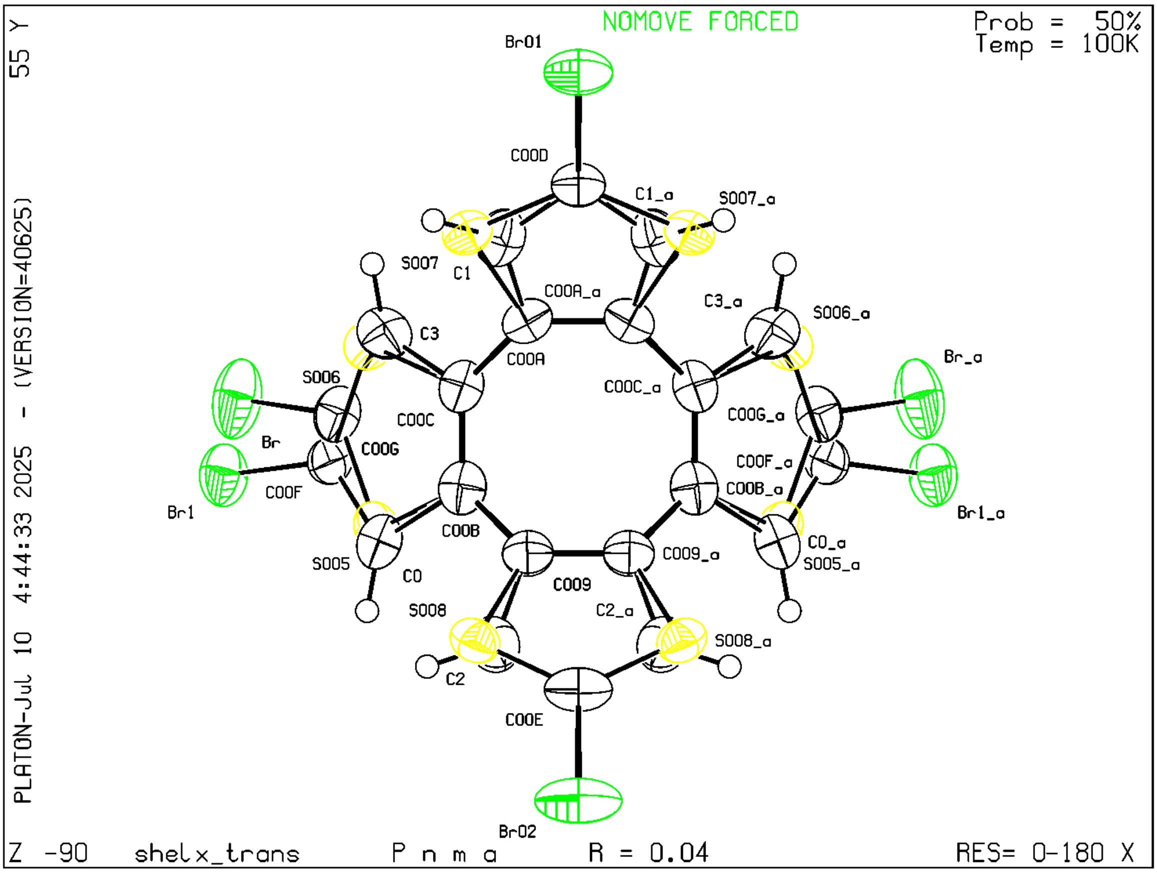

Supplement: Supplementary file 2 — Supporting Information [file CHEM-31-e02872-s001.zip › Br_host_100K.pdf]
