# Supplementary material for: Porous Organic Frameworks Utilizing Halogen···Halogen Interactions of X4–tetra[2,3]Thienylene (X = Br, I): Guest Dynamics and Dielectric Response
Source: Chemistry. 2025 Nov 10;31(71):e02872. doi: 10.1002/chem.202502872 (PMC12734654; doi:10.1002/chem.202502872)

checkCIF/PLATON report

Structure factors have been supplied for datablock(s) shelx_trans

THIS REPORT IS FOR GUIDANCE ONLY. IF USED AS PART OF A REVIEW PROCEDURE FOR PUBLICATION, IT SHOULD NOT REPLACE THE EXPERTISE OF AN EXPERIENCED CRYSTALLOGRAPHIC REFEREE.

No syntax errors found.   CIF dictionary   Interpreting this report

Datablock: shelx_trans

Bond precision:	C-C = 0.0052 A		Wavelength=1.54180
Cell:	a=10.8442(4)	b=12.7904(5)	c=18.5673(16)
	alpha=90	beta=105.255(7)	gamma=90
Temperature:	293 K		

	Calculated	Reported
Volume	2484.6(3)	2484.6(3)
Space group	I 2/a	I 1 2/a 1
Hall group	-I 2ya	-I 2ya
Moiety formula	C16 H4 Br4 S4, C7 H8	C16 H4 Br4 S4, C7 H8
Sum formula	C23 H12 Br4 S4	C23 H12 Br4 S4
Mr	736.17	736.21
Dx,g cm-3	1.968	1.968
Z	4	4
Mu (mm-1)	11.133	11.134
F000	1416.0	1416.0
F000'	1412.31	
h,k,lmax	13,15,22	13,15,22
Nref	2278	2273
Tmin,Tmax		0.440,1.000
Tmin'		

Correction method= # Reported T Limits: Tmin=0.440 Tmax=1.000
AbsCorr = EMPIRICAL

Data completeness= 0.998   Theta(max)= 68.219

R(reflections)= 0.0391(2088)   wR2(reflections)= 0.0953(2273)

S = 1.111   Npar= 150

The following ALERTS were generated. Each ALERT has the format
test-name_ALERT_alert-type_alert-level.
Click on the hyperlinks for more details of the test.

Alert level C

```
PLAT053_ALERT_1_C Minimum Crystal Dimension Missing (or Error) ...     Please Check
PLAT054_ALERT_1_C Medium  Crystal Dimension Missing (or Error) ...     Please Check
PLAT055_ALERT_1_C Maximum Crystal Dimension Missing (or Error) ...     Please Check
PLAT244_ALERT_4_C Low    'Solvent' Ueq as Compared to Neighbors of       C00D Check
PLAT250_ALERT_2_C Large U3/U1 Ratio for <U(i,j)> Tensor(Resd    1)        2.2 Note
PLAT331_ALERT_2_C Small Aver Phenyl C-C Dist C00D     --C00F_b   .       1.36 Ang.
PLAT906_ALERT_3_C Large K Value in the Analysis of Variance ......      3.837 Check
PLAT911_ALERT_3_C Missing FCF Refl Between Thmin & STh/L=    0.600          3 Report
                0  9 17,  -8  0 20,  -2  0 22,
```

Alert level G

```
PLAT002_ALERT_2_G Number of Distance or Angle Restraints on AtSite          4 Note
PLAT083_ALERT_2_G SHELXL Second Parameter in WGHT  Unusually Large       6.14 Why ?
PLAT172_ALERT_4_G The CIF-Embedded .res File Contains DFIX Records          2 Report
PLAT174_ALERT_4_G The CIF-Embedded .res File Contains FLAT Records          1 Report
PLAT199_ALERT_1_G Reported _cell_measurement_temperature ..... (K)        293 Check
PLAT200_ALERT_1_G Reported   _diffrn_ambient_temperature ..... (K)        293 Check
PLAT299_ALERT_4_G Atom Site Occupancy Constrained at ............         0.5 Check
              C5      H00D    H5A     H5B     H5C
PLAT302_ALERT_4_G Anion/Solvent/Minor-Residue Disorder (Resd    2)        14% Note
PLAT720_ALERT_4_G Number of Unusual/Non-Standard Labels ..........         20 Note
              Br01    Br02    S003    S004    C005    C006    C007    C008
              C009    H009    C00A    H00A    C00B    C00C    C00D    C00E
              H00E    C00F    H00F    H00D
PLAT860_ALERT_3_G Number of Least-Squares Restraints ............           5 Note
PLAT912_ALERT_4_G Missing # of FCF Reflections Above STh/L=  0.600          2 Note
PLAT969_ALERT_5_G The 'Henn et al.' R-Factor-gap value ..........       2.918 Note
              Predicted wR2: Based on SigI**2  3.27 or SHELX Weight  8.58
PLAT978_ALERT_2_G Number C-C Bonds with Positive Residual Density.          3 Info
```

```
   0 ALERT level A = Most likely a serious problem - resolve or explain
   0 ALERT level B = A potentially serious problem, consider carefully
   8 ALERT level C = Check. Ensure it is not caused by an omission or oversight
  13 ALERT level G = General information/check it is not something unexpected

   5 ALERT type 1 CIF construction/syntax error, inconsistent or missing data
   5 ALERT type 2 Indicator that the structure model may be wrong or deficient
   3 ALERT type 3 Indicator that the structure quality may be low
   7 ALERT type 4 Improvement, methodology, query or suggestion
   1 ALERT type 5 Informative message, check
```

It is advisable to attempt to resolve as many as possible of the alerts in all categories. Often the minor alerts point to easily fixed oversights, errors and omissions in your CIF or refinement strategy, so attention to these fine details can be worthwhile. In order to resolve some of the more serious problems it may be necessary to carry out additional measurements or structure refinements. However, the purpose of your study may justify the reported deviations and the more serious of these should normally be commented upon in the discussion or experimental section of a paper or in the "special_details" fields of the CIF. checkCIF was carefully designed to identify outliers and unusual parameters, but every test has its limitations and alerts that are not important in a particular case may appear. Conversely, the absence of alerts does not guarantee there are no aspects of the results needing attention. It is up to the individual to critically assess their own results and, if necessary, seek expert advice.

Publication of your CIF in IUCr journals

A basic structural check has been run on your CIF. These basic checks will be run on all CIFs submitted for publication in IUCr journals (*Acta Crystallographica*, *Journal of Applied Crystallography*, *Journal of Synchrotron Radiation*); however, if you intend to submit to *Acta Crystallographica Section C* or *E* or *IUCrData*, you should make sure that full publication checks are run on the final version of your CIF prior to submission.

Publication of your CIF in other journals

Please refer to the *Notes for Authors* of the relevant journal for any special instructions relating to CIF submission.

PLATON version of 04/06/2025; check.def file version of 30/05/2025

Datablock shelx_trans - ellipsoid plot

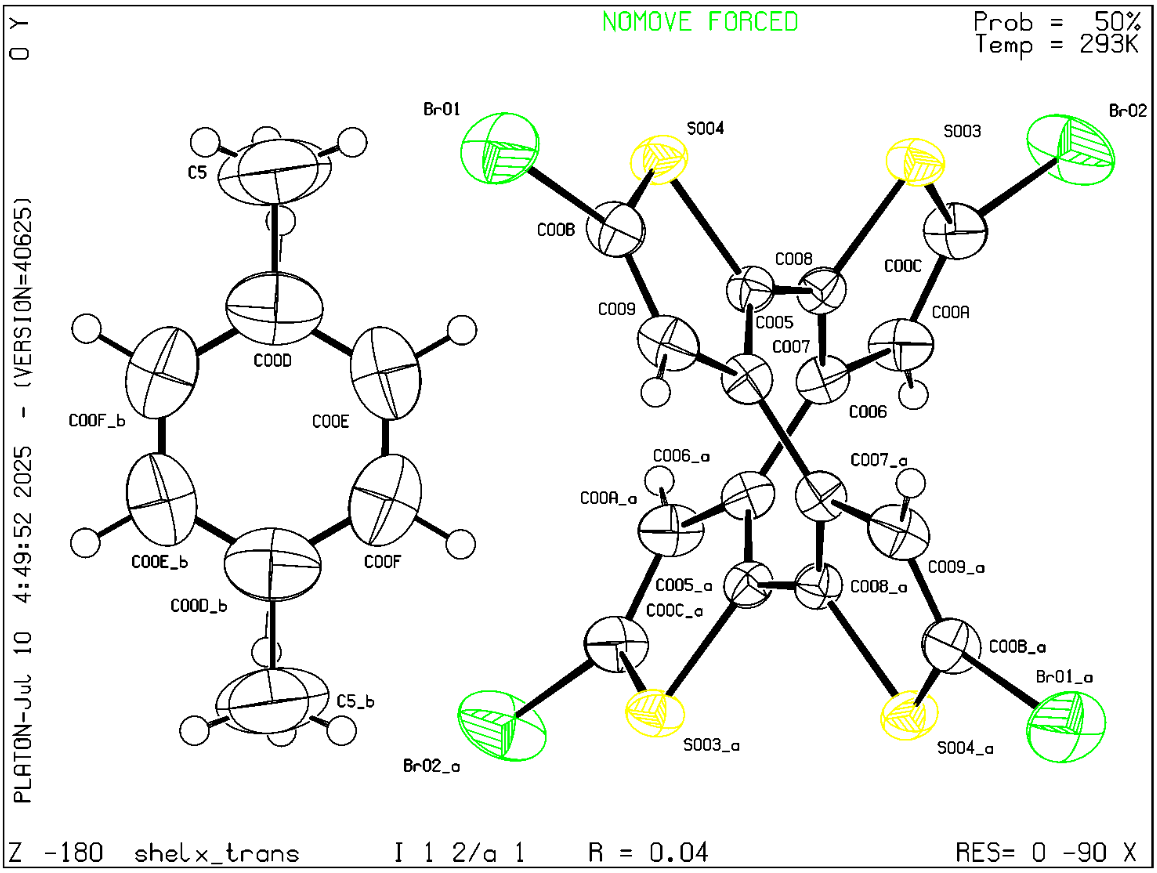

Supplement: Supplementary file 2 — Supporting Information [file CHEM-31-e02872-s001.zip › Br_Tol_293K.pdf]
