# Supplementary material for: Porous Organic Frameworks Utilizing Halogen···Halogen Interactions of X4–tetra[2,3]Thienylene (X = Br, I): Guest Dynamics and Dielectric Response
Source: Chemistry. 2025 Nov 10;31(71):e02872. doi: 10.1002/chem.202502872 (PMC12734654; doi:10.1002/chem.202502872)

checkCIF/PLATON report

Structure factors have been supplied for datablock(s) shelx_trans

THIS REPORT IS FOR GUIDANCE ONLY. IF USED AS PART OF A REVIEW PROCEDURE FOR PUBLICATION, IT SHOULD NOT REPLACE THE EXPERTISE OF AN EXPERIENCED CRYSTALLOGRAPHIC REFEREE.

No syntax errors found. CIF dictionary Interpreting this report

Datablock: shelx_trans

```
Bond precision:   C-C = 0.0066 A          Wavelength=1.54180

Cell:              a=7.8916(2)      b=16.5739(4)      c=14.4346(3)
                   alpha=90         beta=90           gamma=90
Temperature:       100 K

                Calculated                    Reported
Volume          1887.97(8)                    1887.97(8)
Space group     P n m a                       P n m a
Hall group      -P 2ac 2n                     -P 2ac 2n
Moiety formula  C16 H4 Br4 S4                 C16 H4 Br4 S4
Sum formula     C16 H4 Br4 S4                 C16 H4 Br4 S4
Mr              644.03                        644.07
Dx,g cm-3       2.266                         2.266
Z               4                             4
Mu (mm-1)       14.517                        14.517
F000            1216.0                        1216.0
F000'           1211.87
h,k,lmax        9,19,17                       9,19,17
Nref            1794                          1794
Tmin,Tmax                                     0.553,1.000
Tmin'

Correction method= # Reported T Limits: Tmin=0.553 Tmax=1.000
AbsCorr = EMPIRICAL

Data completeness= 1.000          Theta(max)= 68.232

R(reflections)= 0.0440( 1631)                      wR2(reflections)=
                                                   0.0949( 1794)
S = 1.135                  Npar= 169
```

The following ALERTS were generated. Each ALERT has the format
test-name_ALERT_alert-type_alert-level.
Click on the hyperlinks for more details of the test.

Alert level C

```
PLAT053_ALERT_1_C Minimum Crystal Dimension Missing (or Error) ...     Please Check
PLAT054_ALERT_1_C Medium  Crystal Dimension Missing (or Error) ...     Please Check
PLAT055_ALERT_1_C Maximum Crystal Dimension Missing (or Error) ...     Please Check
PLAT234_ALERT_4_C Large Hirshfeld Difference C1       --C00A     .       0.16 Ang.
PLAT234_ALERT_4_C Large Hirshfeld Difference C3       --C00C     .       0.18 Ang.
PLAT341_ALERT_3_C Low Bond Precision on  C-C Bonds ..............     0.0066 Ang.
PLAT906_ALERT_3_C Large K Value in the Analysis of Variance ......      7.058 Check
```

Alert level G

```
PLAT002_ALERT_2_G Number of Distance or Angle Restraints on AtSite          8 Note
PLAT003_ALERT_2_G Number of Uiso or U(i,j)  Restrained non-H-Atoms          5 Report
PLAT083_ALERT_2_G SHELXL Second Parameter in WGHT  Unusually Large       7.16 Why ?
PLAT172_ALERT_4_G The CIF-Embedded .res File Contains DFIX Records          2 Report
PLAT178_ALERT_4_G The CIF-Embedded .res File Contains SIMU Records          1 Report
PLAT186_ALERT_4_G The CIF-Embedded .res File Contains ISOR Records          1 Report
PLAT188_ALERT_3_G A Non-default SIMU Restraint Value has been used     0.0100 Report
PLAT299_ALERT_4_G Atom Site Occupancy Constrained at ............         0.5 Check
              Br      Br1     S005    S006    S007    S008    C1      C2
              C3      C00F    C0      C00G    H1      H0      H2      H3
PLAT301_ALERT_3_G Main Residue  Disorder .............(Resd    1)         50% Note
PLAT720_ALERT_4_G Number of Unusual/Non-Standard Labels ..........         16 Note
              Br01    Br02    S005    S006    S007    S008    C009    C00A
              C00B    C00C    C00D    C00E    C00F    C0      H0      C00G
PLAT764_ALERT_4_G Overcomplete CIF Bond List Detected (Rep/Expd) .       1.16 Ratio
PLAT789_ALERT_4_G Atoms with Negative _atom_site_disorder_group  #         10 Check
PLAT811_ALERT_5_G No ADDSYM Analysis: Too Many Excluded Atoms ....          ! Info
PLAT822_ALERT_4_G CIF-embedded .res Contains Negative PART Numbers          5 Check
PLAT860_ALERT_3_G Number of Least-Squares Restraints ............          35 Note
PLAT883_ALERT_1_G Absent Datum for _atom_sites_solution_primary ..     Please Do !
PLAT969_ALERT_5_G The 'Henn et al.' R-Factor-gap value ..........       3.384 Note
              Predicted wR2: Based on SigI**2  2.80 or SHELX Weight  8.36
PLAT978_ALERT_2_G Number C-C Bonds with Positive Residual Density.          2 Info
```

  0 **ALERT level A** = Most likely a serious problem - resolve or explain
  0 **ALERT level B** = A potentially serious problem, consider carefully
  7 **ALERT level C** = Check. Ensure it is not caused by an omission or oversight
 18 **ALERT level G** = General information/check it is not something unexpected

```
  4 ALERT type 1 CIF construction/syntax error, inconsistent or missing data
  4 ALERT type 2 Indicator that the structure model may be wrong or deficient
  5 ALERT type 3 Indicator that the structure quality may be low
 10 ALERT type 4 Improvement, methodology, query or suggestion
  2 ALERT type 5 Informative message, check
```

It is advisable to attempt to resolve as many as possible of the alerts in all categories. Often the minor alerts point to easily fixed oversights, errors and omissions in your CIF or refinement strategy, so attention to these fine details can be worthwhile. In order to resolve some of the more serious problems it may be necessary to carry out additional measurements or structure refinements. However, the purpose of your study may justify the reported deviations and the more serious of these should normally be commented upon in the discussion or experimental section of a paper or in the "special_details" fields of the CIF. checkCIF was carefully designed to identify outliers and unusual parameters, but every test has its limitations and alerts that are not important in a particular case may appear. Conversely, the absence of alerts does not guarantee there are no aspects of the results needing attention. It is up to the individual to critically assess their own results and, if necessary, seek expert advice.

Publication of your CIF in IUCr journals

A basic structural check has been run on your CIF. These basic checks will be run on all CIFs submitted for publication in IUCr journals (*Acta Crystallographica*, *Journal of Applied Crystallography*, *Journal of Synchrotron Radiation*); however, if you intend to submit to *Acta Crystallographica Section C* or *E* or *IUCrData*, you should make sure that full publication checks are run on the final version of your CIF prior to submission.

Publication of your CIF in other journals

Please refer to the *Notes for Authors* of the relevant journal for any special instructions relating to CIF submission.

PLATON version of 04/06/2025; check.def file version of 30/05/2025

Datablock shelx_trans - ellipsoid plot

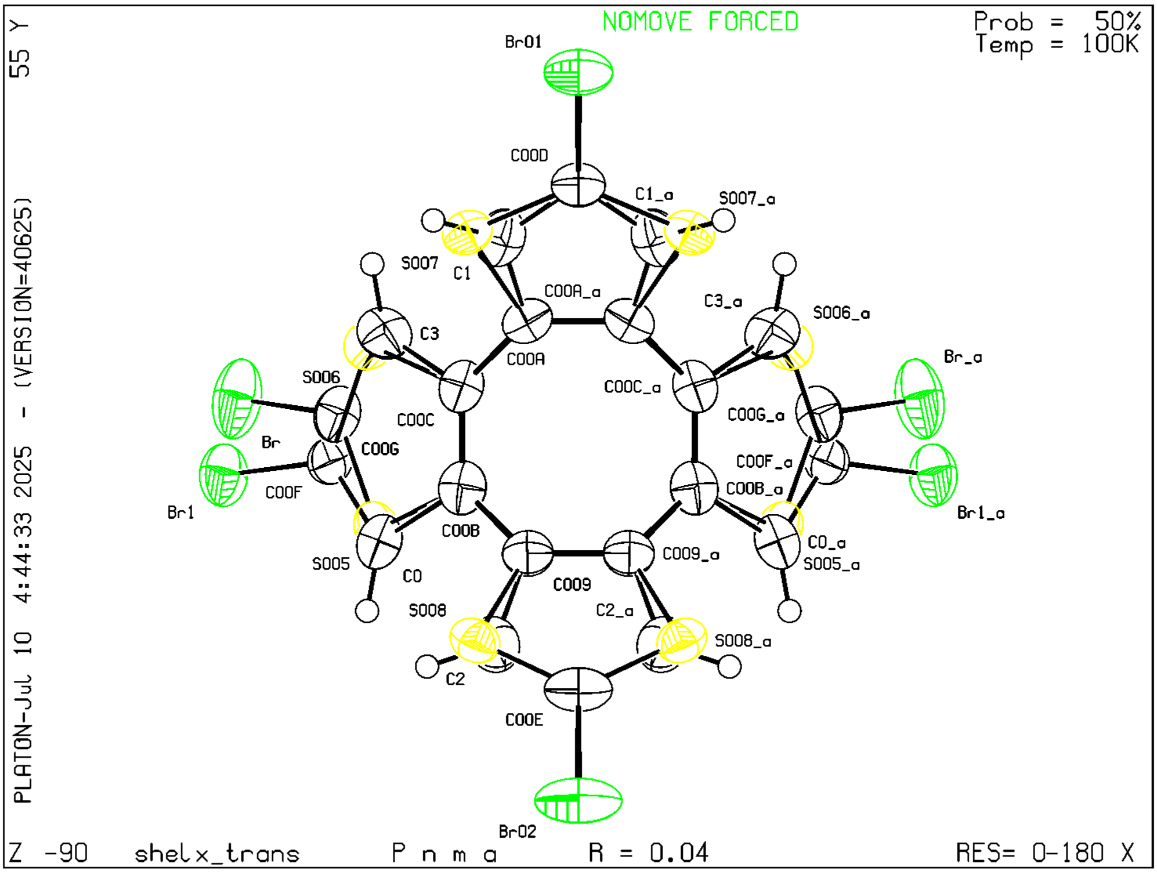

checkCIF/PLATON report

Structure factors have been supplied for datablock(s) shelx

THIS REPORT IS FOR GUIDANCE ONLY. IF USED AS PART OF A REVIEW PROCEDURE FOR PUBLICATION, IT SHOULD NOT REPLACE THE EXPERTISE OF AN EXPERIENCED CRYSTALLOGRAPHIC REFEREE.

No syntax errors found. CIF dictionary Interpreting this report

Datablock: shelx

```
Bond precision:   C-C = 0.0174 A          Wavelength=1.54180

Cell:              a=8.1735(6)      b=17.3132(13)      c=14.9519(9)
                   alpha=90         beta=90            gamma=90
Temperature:       100 K

                 Calculated                  Reported
Volume           2115.8(3)                   2115.8(3)
Space group      P n m a                     P n m a
Hall group       -P 2ac 2n                   -P 2ac 2n
Moiety formula   C16 H4 I4 S4                C16 H4 I4 S4
Sum formula      C16 H4 I4 S4                C16 H4 I4 S4
Mr               832.03                      832.03
Dx,g cm-3        2.612                       2.612
Z                4                           4
Mu (mm-1)        49.933                      49.932
F000             1504.0                      1504.0
F000'            1509.92
h,k,lmax         9,20,18                     9,20,17
Nref             2002                        2001
Tmin,Tmax        0.006,0.007                 0.300,1.000
Tmin'            0.000

Correction method= # Reported T Limits: Tmin=0.300 Tmax=1.000
AbsCorr = EMPIRICAL
```

Data completeness= 1.000 Theta(max)= 68.219

R(reflections)= 0.0698(1040) wR2(reflections)= 0.1833(2001)

S = 0.973 Npar= 169

The following ALERTS were generated. Each ALERT has the format
test-name_ALERT_alert-type_alert-level.
Click on the hyperlinks for more details of the test.

Alert level B

```
RINTA01_ALERT_3_B  The value of Rint is greater than 0.18
            Rint given   0.183
```

Alert level C

```
PLAT234_ALERT_4_C Large Hirshfeld Difference I001     --C009     .       0.18 Ang.
PLAT234_ALERT_4_C Large Hirshfeld Difference S006     --C00D     .       0.18 Ang.
PLAT342_ALERT_3_C Low Bond Precision on  C-C Bonds ..............      0.0174 Ang.
PLAT906_ALERT_3_C Large K Value in the Analysis of Variance ......     16.632 Check
PLAT906_ALERT_3_C Large K Value in the Analysis of Variance ......      6.142 Check
PLAT906_ALERT_3_C Large K Value in the Analysis of Variance ......      2.244 Check
```

Alert level G

```
PLAT002_ALERT_2_G Number of Distance or Angle Restraints on AtSite         14 Note
PLAT003_ALERT_2_G Number of Uiso or U(i,j)  Restrained non-H-Atoms          7 Report
PLAT020_ALERT_3_G The Value of Rint is Greater Than 0.12 .........      0.183 Report
PLAT172_ALERT_4_G The CIF-Embedded .res File Contains DFIX Records          4 Report
PLAT178_ALERT_4_G The CIF-Embedded .res File Contains SIMU Records          1 Report
PLAT186_ALERT_4_G The CIF-Embedded .res File Contains ISOR Records          1 Report
PLAT188_ALERT_3_G A Non-default SIMU Restraint Value has been used     0.0100 Report
PLAT299_ALERT_4_G Atom Site Occupancy Constrained at ............         0.5 Check
              I003    I004    S005    S006    S007    S0      C1      C008
              C2      C3      C3A     C4      H1      H008    H2      H4
PLAT301_ALERT_3_G Main Residue  Disorder .............(Resd    1)         50% Note
PLAT720_ALERT_4_G Number of Unusual/Non-Standard Labels .........          16 Note
              I001    I002    I003    I004    S005    S006    S007    C008
              H008    C009    C00A    C00B    C00C    C00D    C00E    S0
PLAT764_ALERT_4_G Overcomplete CIF Bond List Detected (Rep/Expd) .       1.16 Ratio
PLAT789_ALERT_4_G Atoms with Negative _atom_site_disorder_group  #         10 Check
PLAT811_ALERT_5_G No ADDSYM Analysis: Too Many Excluded Atoms ....          ! Info
PLAT822_ALERT_4_G CIF-embedded .res Contains Negative PART Numbers          7 Check
PLAT860_ALERT_3_G Number of Least-Squares Restraints ............          46 Note
PLAT883_ALERT_1_G Absent Datum for _atom_sites_solution_primary ..     Please Do !
PLAT969_ALERT_5_G The 'Henn et al.' R-Factor-gap value ..........       2.092 Note
              Predicted wR2: Based on SigI**2  8.76 or SHELX Weight 18.85
PLAT978_ALERT_2_G Number C-C Bonds with Positive Residual Density.          0 Info
```

---

0 **ALERT level A** = Most likely a serious problem - resolve or explain
1 **ALERT level B** = A potentially serious problem, consider carefully
6 **ALERT level C** = Check. Ensure it is not caused by an omission or oversight

18 **ALERT level G** = General information/check it is not something unexpected

1 ALERT type 1 CIF construction/syntax error, inconsistent or missing data
3 ALERT type 2 Indicator that the structure model may be wrong or deficient
9 ALERT type 3 Indicator that the structure quality may be low
10 ALERT type 4 Improvement, methodology, query or suggestion
2 ALERT type 5 Informative message, check

It is advisable to attempt to resolve as many as possible of the alerts in all categories. Often the minor alerts point to easily fixed oversights, errors and omissions in your CIF or refinement strategy, so attention to these fine details can be worthwhile. It is up to the individual to critically assess their own results and, if necessary, seek expert advice.

PLATON version of 04/06/2025; check.def file version of 30/05/2025

Datablock shelx - ellipsoid plot

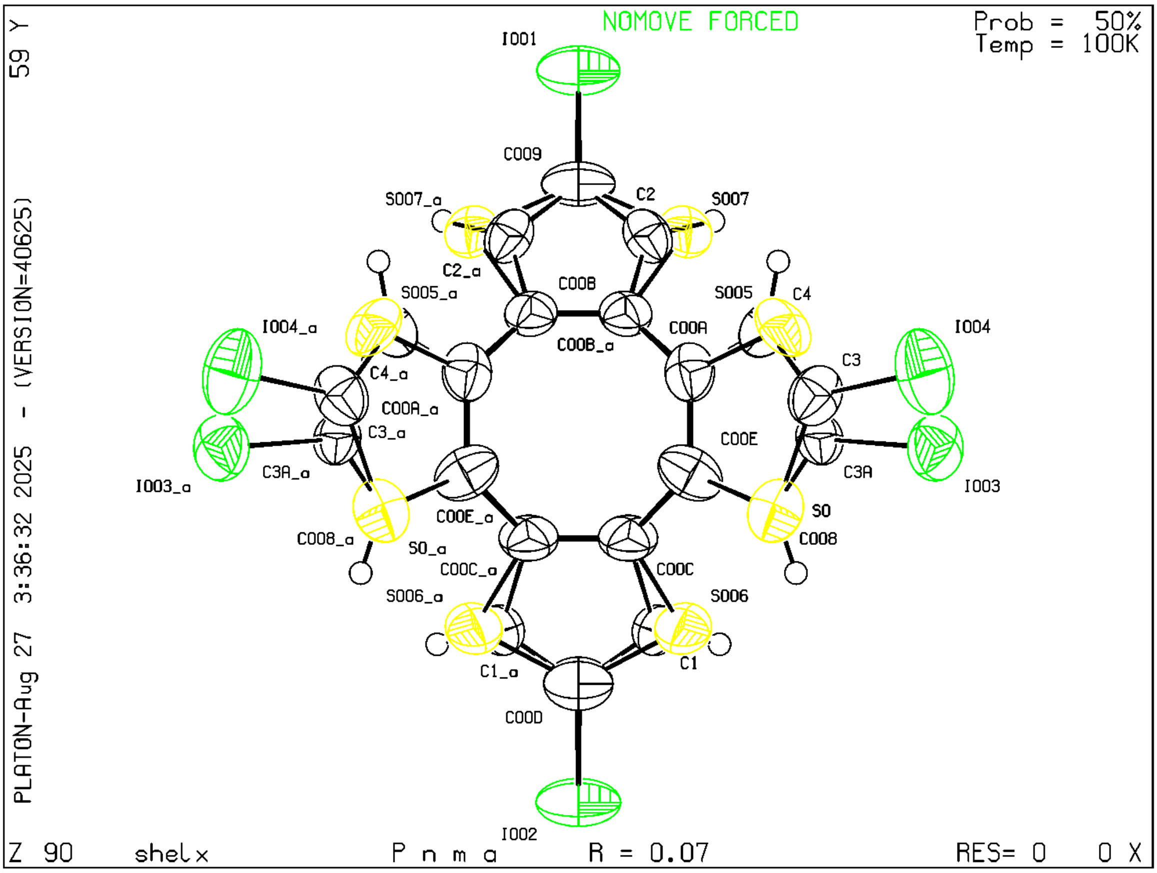

checkCIF/PLATON report

Structure factors have been supplied for datablock(s) shelx_trans

THIS REPORT IS FOR GUIDANCE ONLY. IF USED AS PART OF A REVIEW PROCEDURE FOR PUBLICATION, IT SHOULD NOT REPLACE THE EXPERTISE OF AN EXPERIENCED CRYSTALLOGRAPHIC REFEREE.

No syntax errors found.   CIF dictionary   Interpreting this report

Datablock: shelx_trans

Bond precision:	C-C = 0.0049 A		Wavelength=1.54180
Cell:	a=10.6989(4)	b=12.7048(5)	c=18.2406(16)
	alpha=90	beta=105.736(7)	gamma=90
Temperature:	100 K		

	Calculated	Reported
Volume	2386.5(3)	2386.5(3)
Space group	I 2/a	I 1 2/a 1
Hall group	-I 2ya	-I 2ya
Moiety formula	C16 H4 Br4 S4, C6 H6	C16 H4 Br4 S4, C6 H6
Sum formula	C22 H10 Br4 S4	C22 H10 Br4 S4
Mr	722.14	722.18
Dx,g cm-3	2.010	2.010
Z	4	4
Mu (mm-1)	11.576	11.576
F000	1384.0	1384.0
F000'	1380.25	
h,k,lmax	12,15,21	12,15,21
Nref	2182	2176
Tmin,Tmax		0.373,0.998
Tmin'		

Correction method= # Reported T Limits: Tmin=0.373 Tmax=0.998
AbsCorr = EMPIRICAL

Data completeness= 0.997   Theta(max)= 68.245

R(reflections)= 0.0362(2074)   wR2(reflections)= 0.0979(2176)

S = 1.128   Npar= 136

The following ALERTS were generated. Each ALERT has the format
test-name_ALERT_alert-type_alert-level.
Click on the hyperlinks for more details of the test.

Alert level C

```
PLAT053_ALERT_1_C Minimum Crystal Dimension Missing (or Error) ...     Please Check
PLAT054_ALERT_1_C Medium  Crystal Dimension Missing (or Error) ...     Please Check
PLAT055_ALERT_1_C Maximum Crystal Dimension Missing (or Error) ...     Please Check
PLAT094_ALERT_2_C Ratio of Maximum / Minimum Residual Density ....       2.39 Report
PLAT250_ALERT_2_C Large U3/U1 Ratio for <U(i,j)> Tensor(Resd    1)        2.2 Note
PLAT906_ALERT_3_C Large K Value in the Analysis of Variance ......      2.688 Check
PLAT911_ALERT_3_C Missing FCF Refl Between Thmin & STh/L=    0.600          6 Report
               -9  8  1,  -8  0  2,  11  5  2,  -8  8  2, -10  9  9,   4  0 18,
```

Alert level G

```
PLAT083_ALERT_2_G SHELXL Second Parameter in WGHT  Unusually Large       7.28 Why ?
PLAT720_ALERT_4_G Number of Unusual/Non-Standard Labels ..........         20 Note
              Br01    Br02    S003    S004    C005    C006    C007    C008
              H008    C009    H009    C00A    C00B    C00C    C00D    H00D
              C00E    H00E    C00F    H00F
PLAT969_ALERT_5_G The 'Henn et al.' R-Factor-gap value ...........      2.623 Note
              Predicted wR2: Based on SigI**2  3.73 or SHELX Weight  8.68
PLAT978_ALERT_2_G Number C-C Bonds with Positive Residual Density.          4 Info
```

0 **ALERT level A** = Most likely a serious problem - resolve or explain
0 **ALERT level B** = A potentially serious problem, consider carefully
7 **ALERT level C** = Check. Ensure it is not caused by an omission or oversight
4 **ALERT level G** = General information/check it is not something unexpected

3 ALERT type 1 CIF construction/syntax error, inconsistent or missing data
4 ALERT type 2 Indicator that the structure model may be wrong or deficient
2 ALERT type 3 Indicator that the structure quality may be low
1 ALERT type 4 Improvement, methodology, query or suggestion
1 ALERT type 5 Informative message, check

It is advisable to attempt to resolve as many as possible of the alerts in all categories. Often the minor alerts point to easily fixed oversights, errors and omissions in your CIF or refinement strategy, so attention to these fine details can be worthwhile. In order to resolve some of the more serious problems it may be necessary to carry out additional measurements or structure refinements. However, the purpose of your study may justify the reported deviations and the more serious of these should normally be commented upon in the discussion or experimental section of a paper or in the "special_details" fields of the CIF. checkCIF was carefully designed to identify outliers and unusual parameters, but every test has its limitations and alerts that are not important in a particular case may appear. Conversely, the absence of alerts does not guarantee there are no aspects of the results needing attention. It is up to the individual to critically assess their own results and, if necessary, seek expert advice.

Publication of your CIF in IUCr journals

A basic structural check has been run on your CIF. These basic checks will be run on all CIFs submitted for publication in IUCr journals (*Acta Crystallographica*, *Journal of Applied Crystallography*, *Journal of Synchrotron Radiation*); however, if you intend to submit to *Acta Crystallographica Section C* or *E* or *IUCrData*, you should make sure that full publication checks are run on the final version of your CIF prior to submission.

Publication of your CIF in other journals

Please refer to the *Notes for Authors* of the relevant journal for any special instructions relating to CIF submission.

PLATON version of 04/06/2025; check.def file version of 30/05/2025

Datablock shelx_trans - ellipsoid plot

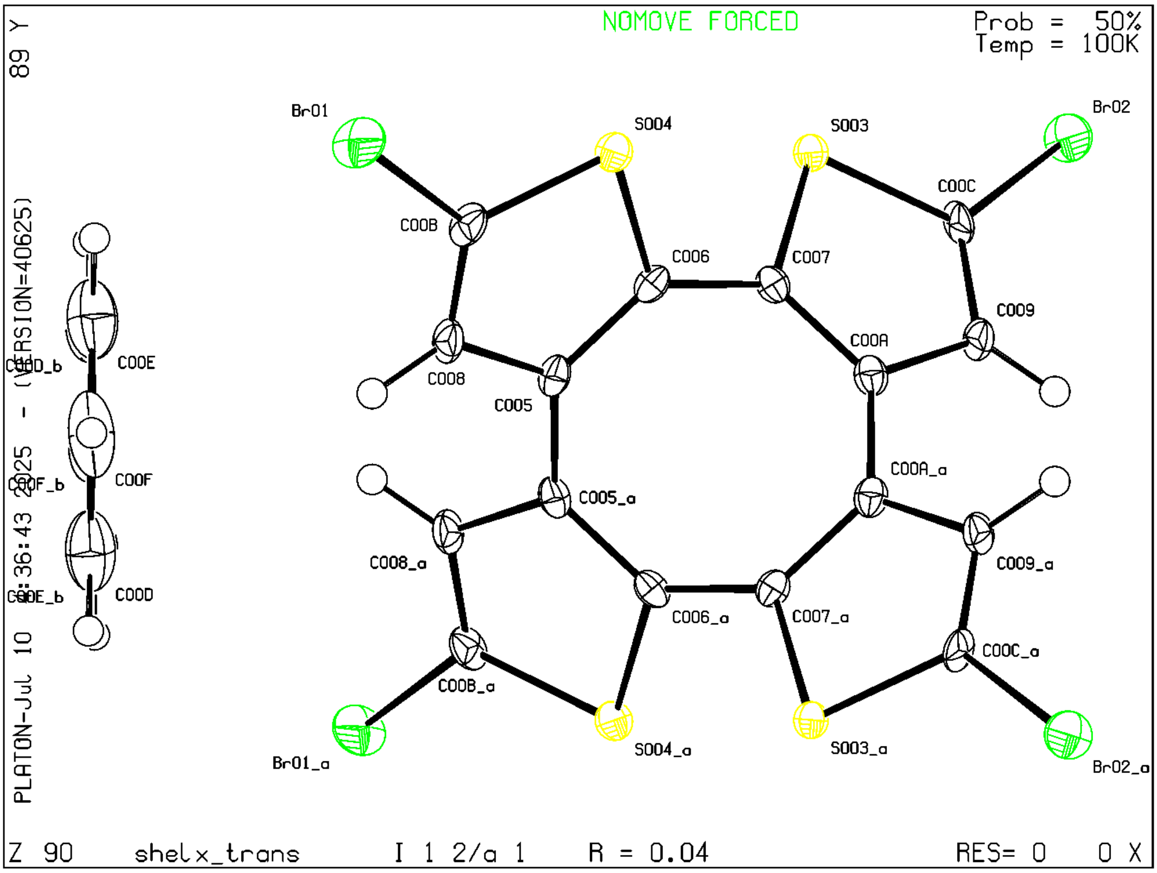

checkCIF/PLATON report

Structure factors have been supplied for datablock(s) shelx_trans

THIS REPORT IS FOR GUIDANCE ONLY. IF USED AS PART OF A REVIEW PROCEDURE FOR PUBLICATION, IT SHOULD NOT REPLACE THE EXPERTISE OF AN EXPERIENCED CRYSTALLOGRAPHIC REFEREE.

No syntax errors found. CIF dictionary Interpreting this report

Datablock: shelx_trans

Bond precision:	C-C = 0.0040 A		Wavelength=1.54180
Cell:	a=10.5686(3)	b=12.7008(4)	c=18.5946(15)
	alpha=90	beta=104.618(7)	gamma=90
Temperature:	100 K		

	Calculated	Reported
Volume	2415.2(2)	2415.2(2)
Space group	I 2/a	I 1 2/a 1
Hall group	-I 2ya	-I 2ya
Moiety formula	C16 H4 Br4 S4, C7 H8	C16 H4 Br4 S4, C7 H8
Sum formula	C23 H12 Br4 S4	C23 H12 Br4 S4
Mr	736.17	736.21
Dx,g cm-3	2.025	2.025
Z	4	4
Mu (mm-1)	11.453	11.454
F000	1416.0	1416.0
F000'	1412.31	
h,k,lmax	12,15,22	12,15,22
Nref	2212	2211
Tmin,Tmax		0.541,1.000
Tmin'		

Correction method= # Reported T Limits: Tmin=0.541 Tmax=1.000
AbsCorr = EMPIRICAL

Data completeness= 1.000 Theta(max)= 68.244

R(reflections)= 0.0271(2070) wR2(reflections)= 0.0704(2211)

S = 1.155 Npar= 150

The following ALERTS were generated. Each ALERT has the format
test-name_ALERT_alert-type_alert-level.
Click on the hyperlinks for more details of the test.

Alert level C

```
PLAT053_ALERT_1_C Minimum Crystal Dimension Missing (or Error) ...     Please Check
PLAT054_ALERT_1_C Medium  Crystal Dimension Missing (or Error) ...     Please Check
PLAT055_ALERT_1_C Maximum Crystal Dimension Missing (or Error) ...     Please Check
PLAT223_ALERT_4_C Solv./Anion  Resd 2  H   Ueq(max)/Ueq(min) Range        6.3 Ratio
PLAT245_ALERT_2_C U(iso) H00F      Smaller than U(eq) C00F      by      0.018 Ang**2
PLAT906_ALERT_3_C Large K Value in the Analysis of Variance ......      3.522 Check
```

Alert level G

```
PLAT002_ALERT_2_G Number of Distance or Angle Restraints on AtSite          2 Note
PLAT172_ALERT_4_G The CIF-Embedded .res File Contains DFIX Records          1 Report
PLAT174_ALERT_4_G The CIF-Embedded .res File Contains FLAT Records          1 Report
PLAT299_ALERT_4_G Atom Site Occupancy Constrained at ............         0.5 Check
              C00G    H00B    H00C    H00G    H00F
PLAT302_ALERT_4_G Anion/Solvent/Minor-Residue Disorder (Resd    2)        14% Note
PLAT720_ALERT_4_G Number of Unusual/Non-Standard Labels ..........         24 Note
              Br01    Br02    S003    S004    C005    C006    C007    C008
              C009    H009    C00A    H00A    C00B    C00C    C00D    H00D
              C00E    H00E    C00F    C00G    H00B    H00C    H00G    H00F
PLAT860_ALERT_3_G Number of Least-Squares Restraints ............          2 Note
PLAT969_ALERT_5_G The 'Henn et al.' R-Factor-gap value ..........      2.606 Note
              Predicted wR2: Based on SigI**2  2.70 or SHELX Weight  6.09
PLAT978_ALERT_2_G Number C-C Bonds with Positive Residual Density.          7 Info
```

0 **ALERT level A** = Most likely a serious problem - resolve or explain
0 **ALERT level B** = A potentially serious problem, consider carefully
6 **ALERT level C** = Check. Ensure it is not caused by an omission or oversight
9 **ALERT level G** = General information/check it is not something unexpected

3 ALERT type 1 CIF construction/syntax error, inconsistent or missing data
3 ALERT type 2 Indicator that the structure model may be wrong or deficient
2 ALERT type 3 Indicator that the structure quality may be low
6 ALERT type 4 Improvement, methodology, query or suggestion
1 ALERT type 5 Informative message, check

It is advisable to attempt to resolve as many as possible of the alerts in all categories. Often the minor alerts point to easily fixed oversights, errors and omissions in your CIF or refinement strategy, so attention to these fine details can be worthwhile. In order to resolve some of the more serious problems it may be necessary to carry out additional measurements or structure refinements. However, the purpose of your study may justify the reported deviations and the more serious of these should normally be commented upon in the discussion or experimental section of a paper or in the "special_details" fields of the CIF. checkCIF was carefully designed to identify outliers and unusual parameters, but every test has its limitations and alerts that are not important in a particular case may appear. Conversely, the absence of alerts does not guarantee there are no aspects of the results needing attention. It is up to the individual to critically assess their own results and, if necessary, seek expert advice.

Publication of your CIF in IUCr journals

A basic structural check has been run on your CIF. These basic checks will be run on all CIFs submitted for publication in IUCr journals (*Acta Crystallographica*, *Journal of Applied Crystallography*, *Journal of Synchrotron Radiation*); however, if you intend to submit to *Acta Crystallographica Section C* or *E* or *IUCrData*, you should make sure that full publication checks are run on the final version of your CIF prior to submission.

Publication of your CIF in other journals

Please refer to the *Notes for Authors* of the relevant journal for any special instructions relating to CIF submission.

PLATON version of 04/06/2025; check.def file version of 30/05/2025

Datablock shelx_trans - ellipsoid plot

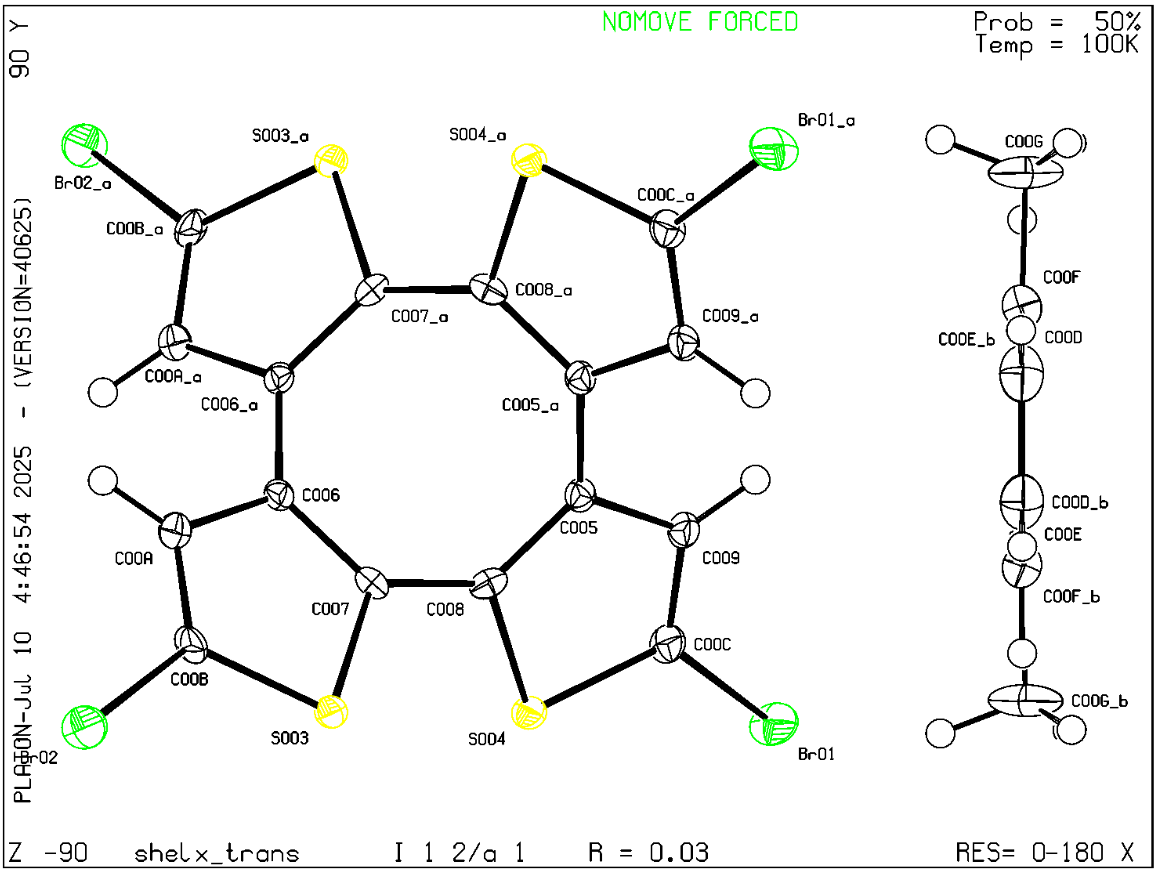

checkCIF/PLATON report

Structure factors have been supplied for datablock(s) shelx_trans

THIS REPORT IS FOR GUIDANCE ONLY. IF USED AS PART OF A REVIEW PROCEDURE FOR PUBLICATION, IT SHOULD NOT REPLACE THE EXPERTISE OF AN EXPERIENCED CRYSTALLOGRAPHIC REFEREE.

No syntax errors found. CIF dictionary Interpreting this report

Datablock: shelx_trans

Bond precision: C-C = 0.0052 A Wavelength=1.54180

Cell:	a=10.8442(4)	b=12.7904(5)	c=18.5673(16)
	alpha=90	beta=105.255(7)	gamma=90
Temperature:	293 K		

	Calculated	Reported
Volume	2484.6(3)	2484.6(3)
Space group	I 2/a	I 1 2/a 1
Hall group	-I 2ya	-I 2ya
Moiety formula	C16 H4 Br4 S4, C7 H8	C16 H4 Br4 S4, C7 H8
Sum formula	C23 H12 Br4 S4	C23 H12 Br4 S4
Mr	736.17	736.21
Dx,g cm-3	1.968	1.968
Z	4	4
Mu (mm-1)	11.133	11.134
F000	1416.0	1416.0
F000'	1412.31	
h,k,lmax	13,15,22	13,15,22
Nref	2278	2273
Tmin,Tmax		0.440,1.000
Tmin'		

Correction method= # Reported T Limits: Tmin=0.440 Tmax=1.000
AbsCorr = EMPIRICAL

Data completeness= 0.998 Theta(max)= 68.219

R(reflections)= 0.0391(2088) wR2(reflections)= 0.0953(2273)

S = 1.111 Npar= 150

The following ALERTS were generated. Each ALERT has the format
test-name_ALERT_alert-type_alert-level.
Click on the hyperlinks for more details of the test.

Alert level C

```
PLAT053_ALERT_1_C Minimum Crystal Dimension Missing (or Error) ...     Please Check
PLAT054_ALERT_1_C Medium  Crystal Dimension Missing (or Error) ...     Please Check
PLAT055_ALERT_1_C Maximum Crystal Dimension Missing (or Error) ...     Please Check
PLAT244_ALERT_4_C Low    'Solvent' Ueq as Compared to Neighbors of       C00D Check
PLAT250_ALERT_2_C Large U3/U1 Ratio for <U(i,j)> Tensor(Resd    1)        2.2 Note
PLAT331_ALERT_2_C Small Aver Phenyl C-C Dist C00D     --C00F_b   .       1.36 Ang.
PLAT906_ALERT_3_C Large K Value in the Analysis of Variance ......      3.837 Check
PLAT911_ALERT_3_C Missing FCF Refl Between Thmin & STh/L=    0.600          3 Report
                0  9 17,  -8  0 20,  -2  0 22,
```

Alert level G

```
PLAT002_ALERT_2_G Number of Distance or Angle Restraints on AtSite          4 Note
PLAT083_ALERT_2_G SHELXL Second Parameter in WGHT  Unusually Large       6.14 Why ?
PLAT172_ALERT_4_G The CIF-Embedded .res File Contains DFIX Records          2 Report
PLAT174_ALERT_4_G The CIF-Embedded .res File Contains FLAT Records          1 Report
PLAT199_ALERT_1_G Reported _cell_measurement_temperature ..... (K)        293 Check
PLAT200_ALERT_1_G Reported   _diffrn_ambient_temperature ..... (K)        293 Check
PLAT299_ALERT_4_G Atom Site Occupancy Constrained at ............         0.5 Check
              C5      H00D    H5A     H5B     H5C
PLAT302_ALERT_4_G Anion/Solvent/Minor-Residue Disorder (Resd    2)        14% Note
PLAT720_ALERT_4_G Number of Unusual/Non-Standard Labels ..........         20 Note
              Br01    Br02    S003    S004    C005    C006    C007    C008
              C009    H009    C00A    H00A    C00B    C00C    C00D    C00E
              H00E    C00F    H00F    H00D
PLAT860_ALERT_3_G Number of Least-Squares Restraints ............           5 Note
PLAT912_ALERT_4_G Missing # of FCF Reflections Above STh/L=  0.600          2 Note
PLAT969_ALERT_5_G The 'Henn et al.' R-Factor-gap value ..........       2.918 Note
              Predicted wR2: Based on SigI**2  3.27 or SHELX Weight  8.58
PLAT978_ALERT_2_G Number C-C Bonds with Positive Residual Density.          3 Info
```

0 **ALERT level A** = Most likely a serious problem - resolve or explain
0 **ALERT level B** = A potentially serious problem, consider carefully
8 **ALERT level C** = Check. Ensure it is not caused by an omission or oversight
13 **ALERT level G** = General information/check it is not something unexpected

5 ALERT type 1 CIF construction/syntax error, inconsistent or missing data
5 ALERT type 2 Indicator that the structure model may be wrong or deficient
3 ALERT type 3 Indicator that the structure quality may be low
7 ALERT type 4 Improvement, methodology, query or suggestion
1 ALERT type 5 Informative message, check

It is advisable to attempt to resolve as many as possible of the alerts in all categories. Often the minor alerts point to easily fixed oversights, errors and omissions in your CIF or refinement strategy, so attention to these fine details can be worthwhile. In order to resolve some of the more serious problems it may be necessary to carry out additional measurements or structure refinements. However, the purpose of your study may justify the reported deviations and the more serious of these should normally be commented upon in the discussion or experimental section of a paper or in the "special_details" fields of the CIF. checkCIF was carefully designed to identify outliers and unusual parameters, but every test has its limitations and alerts that are not important in a particular case may appear. Conversely, the absence of alerts does not guarantee there are no aspects of the results needing attention. It is up to the individual to critically assess their own results and, if necessary, seek expert advice.

Publication of your CIF in IUCr journals

A basic structural check has been run on your CIF. These basic checks will be run on all CIFs submitted for publication in IUCr journals (*Acta Crystallographica*, *Journal of Applied Crystallography*, *Journal of Synchrotron Radiation*); however, if you intend to submit to *Acta Crystallographica Section C* or *E* or *IUCrData*, you should make sure that full publication checks are run on the final version of your CIF prior to submission.

Publication of your CIF in other journals

Please refer to the *Notes for Authors* of the relevant journal for any special instructions relating to CIF submission.

PLATON version of 04/06/2025; check.def file version of 30/05/2025

Datablock shelx_trans - ellipsoid plot

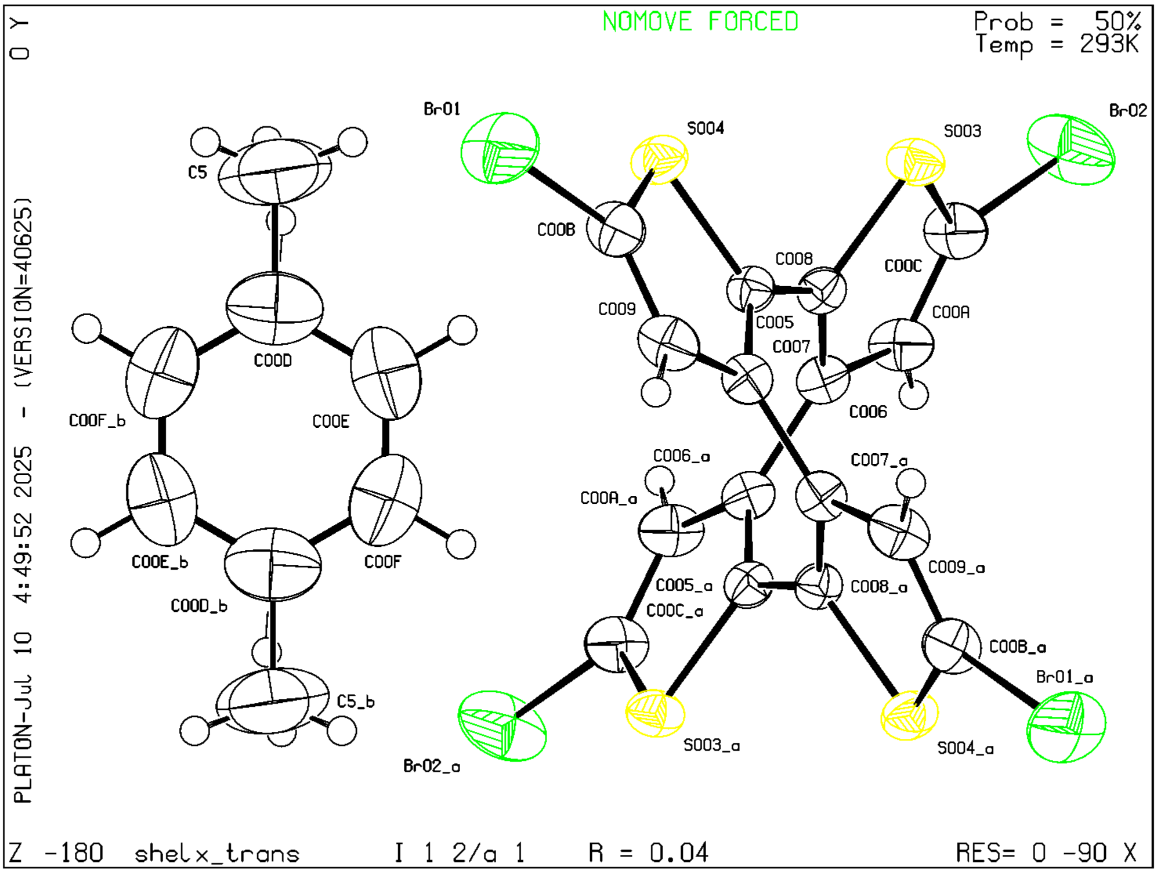

checkCIF/PLATON report

Structure factors have been supplied for datablock(s) shelx_trans

THIS REPORT IS FOR GUIDANCE ONLY. IF USED AS PART OF A REVIEW PROCEDURE FOR PUBLICATION, IT SHOULD NOT REPLACE THE EXPERTISE OF AN EXPERIENCED CRYSTALLOGRAPHIC REFEREE.

No syntax errors found.   CIF dictionary   Interpreting this report

Datablock: shelx_trans

Bond precision:	C-C = 0.0041 A		Wavelength=1.54180
Cell:	a=10.7951(2)	b=12.6150(2)	c=18.4437(14)
	alpha=90	beta=106.109(6)	gamma=90
Temperature:	100 K		

	Calculated	Reported
Volume	2413.1(2)	2413.0(2)
Space group	I 2/a	I 1 2/a 1
Hall group	-I 2ya	-I 2ya
Moiety formula	C16 H4 Br4 S4, C7 H8 O	C16 H4 Br4 S4, C7 H8 O
Sum formula	C23 H12 Br4 O S4	C23 H12 Br4 O S4
Mr	752.17	752.21
Dx,g cm-3	2.070	2.071
Z	4	4
Mu (mm-1)	11.514	11.514
F000	1448.0	1448.0
F000'	1444.50	
h,k,lmax	12,15,22	12,15,22
Nref	2208	2208
Tmin,Tmax		0.446,1.000
Tmin'		

Correction method= # Reported T Limits: Tmin=0.446 Tmax=1.000
AbsCorr = EMPIRICAL

Data completeness= 1.000   Theta(max)= 68.235

R(reflections)= 0.0311(2075)   wR2(reflections)= 0.0809(2208)

S = 1.148   Npar= 170

The following ALERTS were generated. Each ALERT has the format
test-name_ALERT_alert-type_alert-level.
Click on the hyperlinks for more details of the test.

Alert level C

```
PLAT053_ALERT_1_C Minimum Crystal Dimension Missing (or Error) ...     Please Check
PLAT054_ALERT_1_C Medium  Crystal Dimension Missing (or Error) ...     Please Check
PLAT055_ALERT_1_C Maximum Crystal Dimension Missing (or Error) ...     Please Check
PLAT906_ALERT_3_C Large K Value in the Analysis of Variance ......      3.267 Check
PLAT934_ALERT_3_C Number of (Iobs-Icalc)/Sigma(W) > 10 Outliers ..          1 Check
              -10  0 18,
```

Alert level G

```
PLAT083_ALERT_2_G SHELXL Second Parameter in WGHT  Unusually Large       7.61 Why ?
PLAT174_ALERT_4_G The CIF-Embedded .res File Contains FLAT Records          1 Report
PLAT299_ALERT_4_G Atom Site Occupancy Constrained at ............         0.5 Check
              O00E    C1      C2      C4      C5      C00D    C00F    C00H
              H2      H00D    H4      H00F    H00H    H5A     H5B     H5C
PLAT302_ALERT_4_G Anion/Solvent/Minor-Residue Disorder (Resd    2)       100% Note
PLAT720_ALERT_4_G Number of Unusual/Non-Standard Labels ..........         21 Note
              Br01    Br02    S003    S004    C005    C006    C007    C008
              H008    C009    H009    C00A    C00B    C00C    C00D    H00D
              C00F    H00F    C00H    H00H    O00E
PLAT789_ALERT_4_G Atoms with Negative _atom_site_disorder_group  #         16 Check
PLAT822_ALERT_4_G CIF-embedded .res Contains Negative PART Numbers          1 Check
PLAT860_ALERT_3_G Number of Least-Squares Restraints ............           1 Note
PLAT969_ALERT_5_G The 'Henn et al.' R-Factor-gap value ..........       3.099 Note
              Predicted wR2: Based on SigI**2  2.61 or SHELX Weight  7.05
PLAT978_ALERT_2_G Number C-C Bonds with Positive Residual Density.          2 Info
```

0 **ALERT level A** = Most likely a serious problem - resolve or explain
0 **ALERT level B** = A potentially serious problem, consider carefully
5 **ALERT level C** = Check. Ensure it is not caused by an omission or oversight
10 **ALERT level G** = General information/check it is not something unexpected

3 ALERT type 1 CIF construction/syntax error, inconsistent or missing data
2 ALERT type 2 Indicator that the structure model may be wrong or deficient
3 ALERT type 3 Indicator that the structure quality may be low
6 ALERT type 4 Improvement, methodology, query or suggestion
1 ALERT type 5 Informative message, check

It is advisable to attempt to resolve as many as possible of the alerts in all categories. Often the minor alerts point to easily fixed oversights, errors and omissions in your CIF or refinement strategy, so attention to these fine details can be worthwhile. In order to resolve some of the more serious problems it may be necessary to carry out additional measurements or structure refinements. However, the purpose of your study may justify the reported deviations and the more serious of these should normally be commented upon in the discussion or experimental section of a paper or in the "special_details" fields of the CIF. checkCIF was carefully designed to identify outliers and unusual parameters, but every test has its limitations and alerts that are not important in a particular case may appear. Conversely, the absence of alerts does not guarantee there are no aspects of the results needing attention. It is up to the individual to critically assess their own results and, if necessary, seek expert advice.

Publication of your CIF in IUCr journals

A basic structural check has been run on your CIF. These basic checks will be run on all CIFs submitted for publication in IUCr journals (*Acta Crystallographica*, *Journal of Applied Crystallography*, *Journal of Synchrotron Radiation*); however, if you intend to submit to *Acta Crystallographica Section C* or *E* or *IUCrData*, you should make sure that full publication checks are run on the final version of your CIF prior to submission.

Publication of your CIF in other journals

Please refer to the *Notes for Authors* of the relevant journal for any special instructions relating to CIF submission.

PLATON version of 04/06/2025; check.def file version of 30/05/2025

Datablock shelx_trans - ellipsoid plot

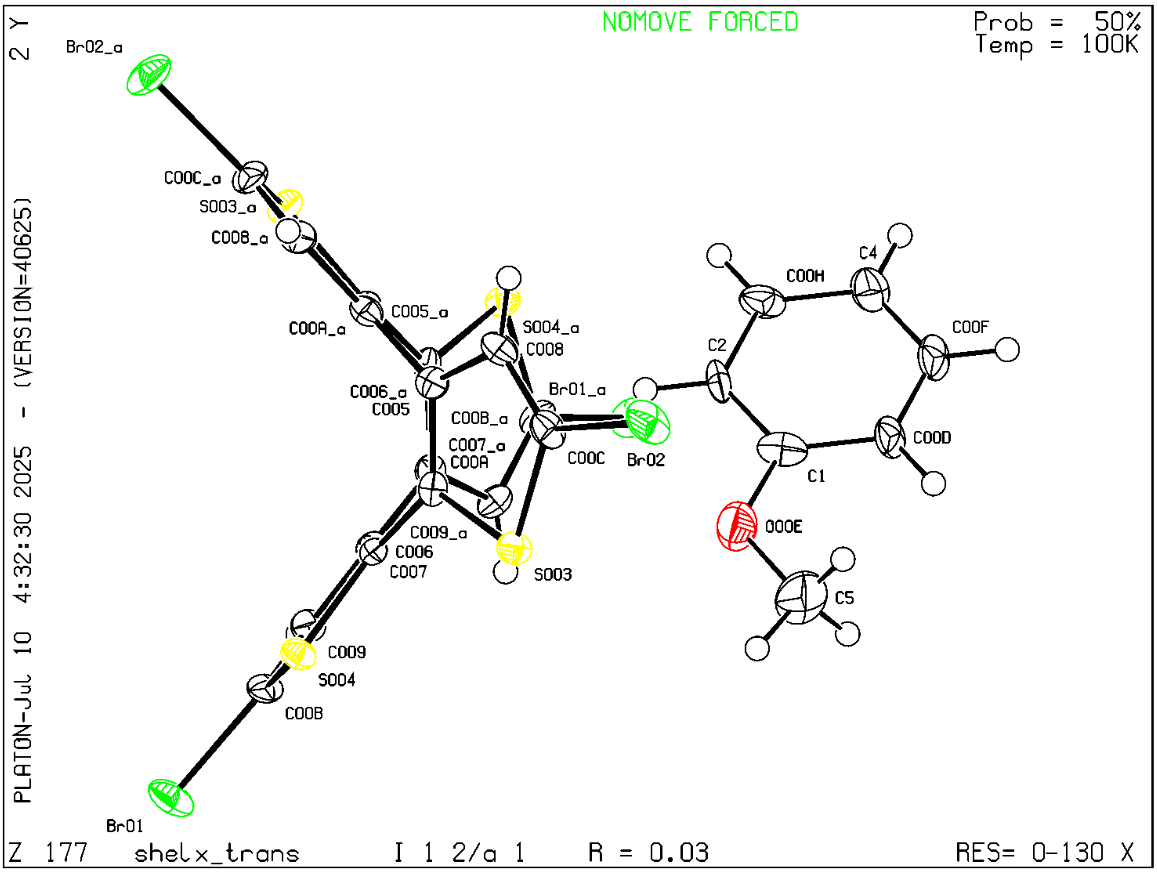

checkCIF/PLATON report

Structure factors have been supplied for datablock(s) shelx_sq

THIS REPORT IS FOR GUIDANCE ONLY. IF USED AS PART OF A REVIEW PROCEDURE FOR PUBLICATION, IT SHOULD NOT REPLACE THE EXPERTISE OF AN EXPERIENCED CRYSTALLOGRAPHIC REFEREE.

No syntax errors found.   CIF dictionary   Interpreting this report

Datablock: shelx_sq

Bond precision:	C-C = 0.0169 A		Wavelength=1.54180
Cell:	a=10.3980(8)	b=13.1641(10)	c=25.809(2)
	alpha=90	beta=90	gamma=90
Temperature:	100 K		

	Calculated	Reported
Volume	3532.7(5)	3532.7(5)
Space group	I b a m	I b a m
Hall group	-I 2 2c	-I 2 2c
Moiety formula	C16 H4 I4 S4, 2(C6 H6) [+ solvent]	C16 H4 I4 S4, 2(C6 H6), 1[]
Sum formula	C28 H16 I4 S4 [+ solvent]	C28 H16 I4 S4
Mr	988.25	988.25
Dx,g cm-3	1.858	1.858
Z	4	4
Mu (mm-1)	30.029	30.029
F000	1840.0	1840.0
F000'	1845.92	
h,k,lmax	12,15,31	12,15,30
Nref	1661	1661
Tmin,Tmax	0.006,0.050	0.262,0.993
Tmin'	0.001	

Correction method= # Reported T Limits: Tmin=0.262 Tmax=0.993
AbsCorr = EMPIRICAL

Data completeness= 1.000   Theta(max)= 68.095

R(reflections)= 0.0582(1306)   wR2(reflections)= 0.1642(1661)

S = 1.111   Npar= 87

The following ALERTS were generated. Each ALERT has the format
test-name_ALERT_alert-type_alert-level.
Click on the hyperlinks for more details of the test.

Alert level C

```
RINTA01_ALERT_3_C  The value of Rint is greater than 0.12
            Rint given   0.128
PLAT243_ALERT_4_C High   'Solvent' Ueq as Compared to Neighbors of       C00B Check
PLAT342_ALERT_3_C Low Bond Precision on  C-C Bonds ...............     0.0169 Ang.
PLAT906_ALERT_3_C Large K Value in the Analysis of Variance ......      3.297 Check
PLAT971_ALERT_2_C Check Calcd Resid. Dens.  0.91Ang From I001            2.43 eA-3
```

Alert level G

```
PLAT002_ALERT_2_G Number of Distance or Angle Restraints on AtSite          5 Note
PLAT003_ALERT_2_G Number of Uiso or U(i,j)  Restrained non-H-Atoms          3 Report
PLAT020_ALERT_3_G The Value of Rint is Greater Than 0.12 .........      0.128 Report
PLAT042_ALERT_1_G Calc. and Reported MoietyFormula Strings  Differ     Please Check
              Calc: C16 H4 I4 S4, 2(C6 H6)
              Rep.: C16 H4 I4 S4, 2(C6 H6), 1[]
PLAT083_ALERT_2_G SHELXL Second Parameter in WGHT  Unusually Large      46.82 Why ?
PLAT172_ALERT_4_G The CIF-Embedded .res File Contains DFIX Records          2 Report
PLAT186_ALERT_4_G The CIF-Embedded .res File Contains ISOR Records          1 Report
PLAT605_ALERT_4_G Largest Solvent Accessible VOID in the Structure        179 A**3
PLAT720_ALERT_4_G Number of Unusual/Non-Standard Labels ..........         17 Note
              I001    S002    C003    H003    C004    C005    C006    C007
              H007    C008    H008    C009    H009    C00A    H00A    C00B
              H00B
PLAT764_ALERT_4_G Overcomplete CIF Bond List Detected (Rep/Expd) .       1.15 Ratio
PLAT860_ALERT_3_G Number of Least-Squares Restraints .............         22 Note
PLAT869_ALERT_4_G ALERTS Related to the Use of SQUEEZE  Suppressed          ! Info
PLAT883_ALERT_1_G Absent Datum for _atom_sites_solution_primary ..     Please Do !
PLAT909_ALERT_3_G Percentage of I>2sig(I) Data at Theta(Max) Still        59% Note
PLAT969_ALERT_5_G The 'Henn et al.' R-Factor-gap value ...........      2.591 Note
              Predicted wR2: Based on SigI**2  6.34 or SHELX Weight 14.82
PLAT978_ALERT_2_G Number C-C Bonds with Positive Residual Density.          1 Info
```

0 **ALERT level A** = Most likely a serious problem - resolve or explain
0 **ALERT level B** = A potentially serious problem, consider carefully
5 **ALERT level C** = Check. Ensure it is not caused by an omission or oversight
16 **ALERT level G** = General information/check it is not something unexpected

2 ALERT type 1 CIF construction/syntax error, inconsistent or missing data
5 ALERT type 2 Indicator that the structure model may be wrong or deficient
6 ALERT type 3 Indicator that the structure quality may be low
7 ALERT type 4 Improvement, methodology, query or suggestion
1 ALERT type 5 Informative message, check

It is advisable to attempt to resolve as many as possible of the alerts in all categories. Often the minor alerts point to easily fixed oversights, errors and omissions in your CIF or refinement strategy, so attention to these fine details can be worthwhile. In order to resolve some of the more serious problems it may be necessary to carry out additional measurements or structure refinements. However, the purpose of your study may justify the reported deviations and the more serious of these should normally be commented upon in the discussion or experimental section of a paper or in the "special_details" fields of the CIF. checkCIF was carefully designed to identify outliers and unusual parameters, but every test has its limitations and alerts that are not important in a particular case may appear. Conversely, the absence of alerts does not guarantee there are no aspects of the results needing attention. It is up to the individual to critically assess their own results and, if necessary, seek expert advice.

Publication of your CIF in IUCr journals

A basic structural check has been run on your CIF. These basic checks will be run on all CIFs submitted for publication in IUCr journals (*Acta Crystallographica*, *Journal of Applied Crystallography*, *Journal of Synchrotron Radiation*); however, if you intend to submit to *Acta Crystallographica Section C* or *E* or *IUCrData*, you should make sure that full publication checks are run on the final version of your CIF prior to submission.

Publication of your CIF in other journals

Please refer to the *Notes for Authors* of the relevant journal for any special instructions relating to CIF submission.

PLATON version of 04/06/2025; check.def file version of 30/05/2025

Datablock shelx_sq - ellipsoid plot

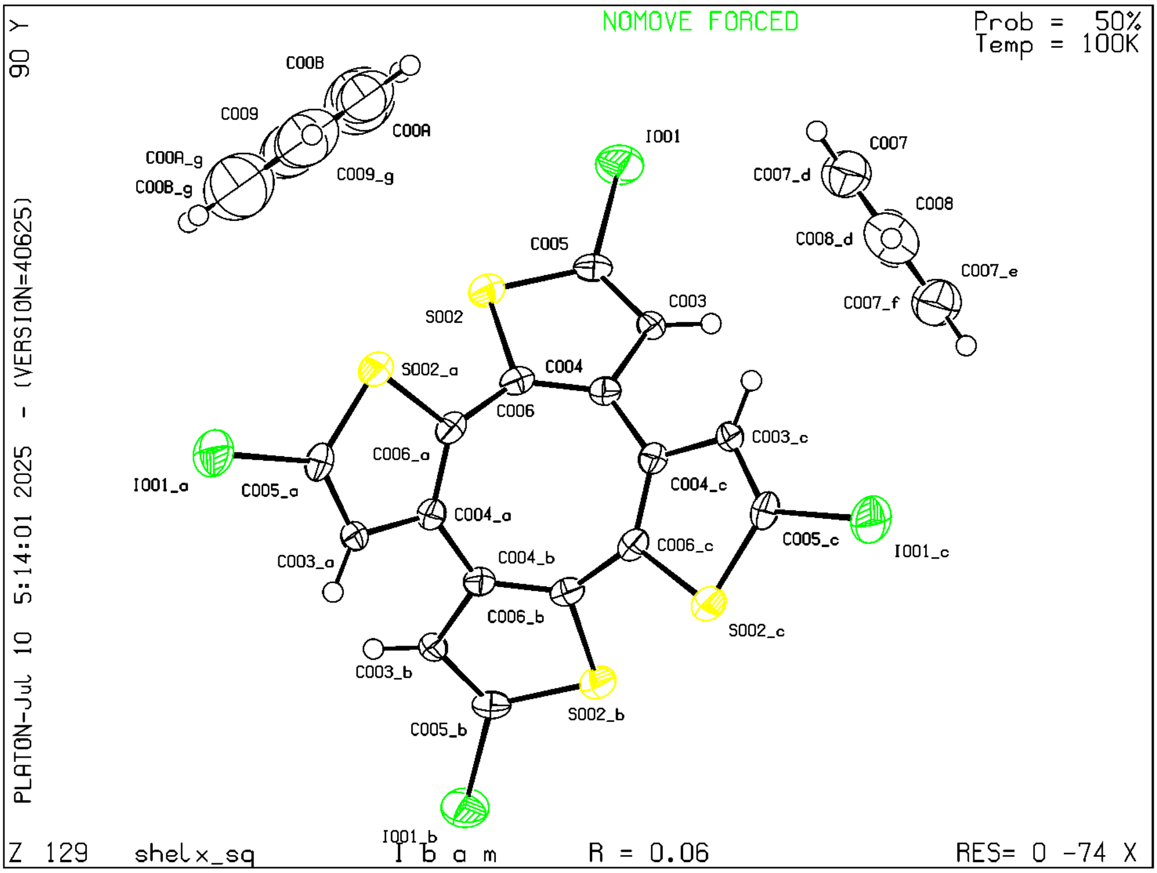

checkCIF/PLATON report

Structure factors have been supplied for datablock(s) shelx

THIS REPORT IS FOR GUIDANCE ONLY. IF USED AS PART OF A REVIEW PROCEDURE FOR PUBLICATION, IT SHOULD NOT REPLACE THE EXPERTISE OF AN EXPERIENCED CRYSTALLOGRAPHIC REFEREE.

No syntax errors found.   CIF dictionary   Interpreting this report

Datablock: shelx

```
Bond precision:   C-C = 0.0194 A          Wavelength=1.54180

Cell:             a=10.3141(5)      b=13.1039(6)      c=25.5756(12)
                  alpha=90          beta=90           gamma=90
Temperature:      100 K

                Calculated                    Reported
Volume          3456.7(3)                     3456.7(3)
Space group     I b a m                       I b a m
Hall group      -I 2 2c                       -I 2 2c
Moiety formula  C16 H4 I4 S4, 3(C6 H6)        C16 H4 I4 S4, 3(C6 H6)
Sum formula     C34 H22 I4 S4                 C34 H22 I4 S4
Mr              1066.36                       1066.35
Dx,g cm-3       2.049                         2.049
Z               4                             4
Mu (mm-1)       30.752                        30.752
F000            2008.0                        2008.0
F000'           2014.02
h,k,lmax        12,15,30                      12,15,30
Nref            1635                          1632
Tmin,Tmax       0.025,0.046                   0.287,1.000
Tmin'           0.004

Correction method= # Reported T Limits: Tmin=0.287 Tmax=1.000
AbsCorr = EMPIRICAL

Data completeness= 0.998          Theta(max)= 68.237

                                                     wR2(reflections)=
R(reflections)= 0.0660( 1443)                        0.1667( 1632)

S = 1.171                  Npar= 102
```

The following ALERTS were generated. Each ALERT has the format
test-name_ALERT_alert-type_alert-level.
Click on the hyperlinks for more details of the test.

Alert level C

```
PLAT342_ALERT_3_C Low Bond Precision on  C-C Bonds ..............    0.01943 Ang.
PLAT906_ALERT_3_C Large K Value in the Analysis of Variance ......      7.849 Check
PLAT906_ALERT_3_C Large K Value in the Analysis of Variance ......      2.140 Check
PLAT911_ALERT_3_C Missing FCF Refl Between Thmin & STh/L=    0.600          3 Report
               12  0  0,  12  0  2,  12  0  6,
PLAT971_ALERT_2_C Check Calcd Resid. Dens.  1.35Ang From I001            1.65 eA-3
PLAT971_ALERT_2_C Check Calcd Resid. Dens.  1.51Ang From I001            1.52 eA-3
```

Alert level G

```
PLAT002_ALERT_2_G Number of Distance or Angle Restraints on AtSite          5 Note
PLAT003_ALERT_2_G Number of Uiso or U(i,j)  Restrained non-H-Atoms          3 Report
PLAT083_ALERT_2_G SHELXL Second Parameter in WGHT  Unusually Large     133.96 Why ?
PLAT172_ALERT_4_G The CIF-Embedded .res File Contains DFIX Records          5 Report
PLAT186_ALERT_4_G The CIF-Embedded .res File Contains ISOR Records          1 Report
PLAT720_ALERT_4_G Number of Unusual/Non-Standard Labels ..........         21 Note
              I001    S002    C003    C004    H004    C005    C006    C007
              H007    C008    H008    C009    H009    C00A    H00A    C00B
              H00B    C00C    H00C    C00D    H00D
PLAT860_ALERT_3_G Number of Least-Squares Restraints ............          23 Note
PLAT883_ALERT_1_G Absent Datum for _atom_sites_solution_primary ..     Please Do !
PLAT969_ALERT_5_G The 'Henn et al.' R-Factor-gap value ..........       3.658 Note
              Predicted wR2: Based on SigI**2  4.56 or SHELX Weight 14.24
PLAT978_ALERT_2_G Number C-C Bonds with Positive Residual Density.          4 Info
```

0 **ALERT level A** = Most likely a serious problem - resolve or explain
0 **ALERT level B** = A potentially serious problem, consider carefully
6 **ALERT level C** = Check. Ensure it is not caused by an omission or oversight
10 **ALERT level G** = General information/check it is not something unexpected

1 ALERT type 1 CIF construction/syntax error, inconsistent or missing data
6 ALERT type 2 Indicator that the structure model may be wrong or deficient
5 ALERT type 3 Indicator that the structure quality may be low
3 ALERT type 4 Improvement, methodology, query or suggestion
1 ALERT type 5 Informative message, check

It is advisable to attempt to resolve as many as possible of the alerts in all categories. Often the minor alerts point to easily fixed oversights, errors and omissions in your CIF or refinement strategy, so attention to these fine details can be worthwhile. In order to resolve some of the more serious problems it may be necessary to carry out additional measurements or structure refinements. However, the purpose of your study may justify the reported deviations and the more serious of these should normally be commented upon in the discussion or experimental section of a paper or in the "special_details" fields of the CIF. checkCIF was carefully designed to identify outliers and unusual parameters, but every test has its limitations and alerts that are not important in a particular case may appear. Conversely, the absence of alerts does not guarantee there are no aspects of the results needing attention. It is up to the individual to critically assess their own results and, if necessary, seek expert advice.

Publication of your CIF in IUCr journals

A basic structural check has been run on your CIF. These basic checks will be run on all CIFs submitted for publication in IUCr journals (*Acta Crystallographica*, *Journal of Applied Crystallography*, *Journal of Synchrotron Radiation*); however, if you intend to submit to *Acta Crystallographica Section C* or *E* or *IUCrData*, you should make sure that full publication checks are run on the final version of your CIF prior to submission.

Publication of your CIF in other journals

Please refer to the *Notes for Authors* of the relevant journal for any special instructions relating to CIF submission.

PLATON version of 04/06/2025; check.def file version of 30/05/2025

Datablock shelx - ellipsoid plot

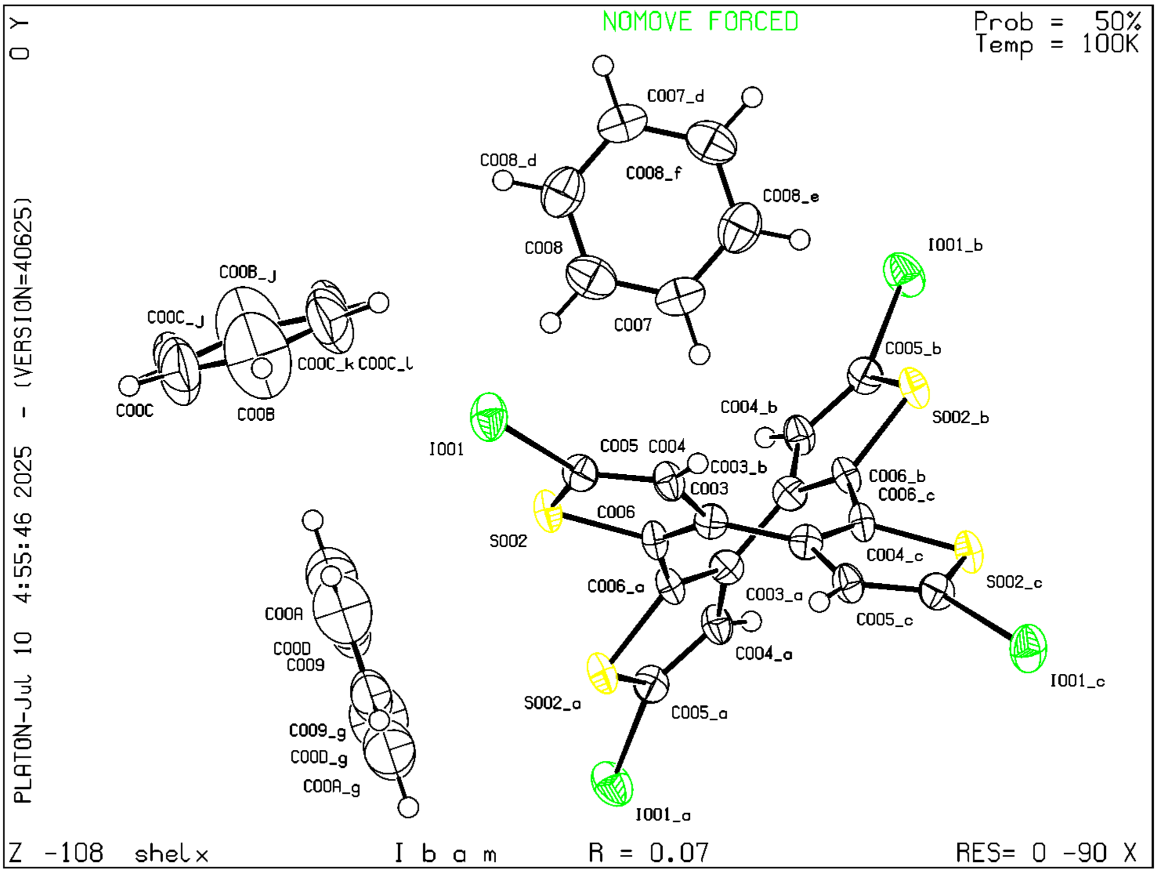

checkCIF/PLATON report

Structure factors have been supplied for datablock(s) shelx_trans

THIS REPORT IS FOR GUIDANCE ONLY. IF USED AS PART OF A REVIEW PROCEDURE FOR PUBLICATION, IT SHOULD NOT REPLACE THE EXPERTISE OF AN EXPERIENCED CRYSTALLOGRAPHIC REFEREE.

No syntax errors found. CIF dictionary Interpreting this report

Datablock: shelx_trans

Bond precision:	C-C = 0.0098 A		Wavelength=1.54180
Cell:	a=10.9151(3)	b=13.0111(4)	c=19.1733(14)
	alpha=90	beta=105.147(6)	gamma=90
Temperature:	100 K		

	Calculated	Reported
Volume	2628.3(2)	2628.3(2)
Space group	I 2/a	I 1 2/a 1
Hall group	-I 2ya	-I 2ya
Moiety formula	C16 H4 I4 S4, C7 H8	C16 H4 I4 S4, C7 H8
Sum formula	C23 H12 I4 S4	C23 H12 I4 S4
Mr	924.17	924.17
Dx,g cm-3	2.336	2.335
Z	4	4
Mu (mm-1)	40.293	40.292
F000	1704.0	1704.0
F000'	1709.87	
h,k,lmax	13,15,23	13,15,23
Nref	2412	2412
Tmin,Tmax		0.191,1.000
Tmin'		

Correction method= # Reported T Limits: Tmin=0.191 Tmax=1.000
AbsCorr = EMPIRICAL

Data completeness= 1.000 Theta(max)= 68.244

R(reflections)= 0.0626(2046) wR2(reflections)= 0.1609(2412)

S = 1.054 Npar= 150

The following ALERTS were generated. Each ALERT has the format
test-name_ALERT_alert-type_alert-level.
Click on the hyperlinks for more details of the test.

Alert level B

```
PLAT971_ALERT_2_B Check Calcd Resid. Dens.  1.05Ang From I001          3.23 eA-3
PLAT971_ALERT_2_B Check Calcd Resid. Dens.  1.10Ang From I002          3.16 eA-3
PLAT971_ALERT_2_B Check Calcd Resid. Dens.  0.90Ang From I002          2.98 eA-3
PLAT971_ALERT_2_B Check Calcd Resid. Dens.  0.91Ang From I001          2.70 eA-3
```

Alert level C

```
RINTA01_ALERT_3_C  The value of Rint is greater than 0.12
            Rint given   0.134
PLAT053_ALERT_1_C Minimum Crystal Dimension Missing (or Error) ...     Please Check
PLAT054_ALERT_1_C Medium  Crystal Dimension Missing (or Error) ...     Please Check
PLAT055_ALERT_1_C Maximum Crystal Dimension Missing (or Error) ...     Please Check
PLAT213_ALERT_2_C Atom C00C            has ADP max/min Ratio .....        3.3 prolat
PLAT250_ALERT_2_C Large U3/U1 Ratio for <U(i,j)> Tensor(Resd    1)        2.7 Note
PLAT342_ALERT_3_C Low Bond Precision on  C-C Bonds ..............    0.00975 Ang.
PLAT971_ALERT_2_C Check Calcd Resid. Dens.  0.92Ang From I001          2.05 eA-3
PLAT971_ALERT_2_C Check Calcd Resid. Dens.  0.92Ang From I002          1.80 eA-3
PLAT972_ALERT_2_C Check Calcd Resid. Dens.  0.88Ang From I001         -1.77 eA-3
PLAT972_ALERT_2_C Check Calcd Resid. Dens.  0.99Ang From I002         -1.58 eA-3
```

Alert level G

```
PLAT002_ALERT_2_G Number of Distance or Angle Restraints on AtSite          2 Note
PLAT020_ALERT_3_G The Value of Rint is Greater Than 0.12 .........      0.134 Report
PLAT072_ALERT_2_G SHELXL First  Parameter in WGHT  Unusually Large       0.10 Report
PLAT172_ALERT_4_G The CIF-Embedded .res File Contains DFIX Records          1 Report
PLAT174_ALERT_4_G The CIF-Embedded .res File Contains FLAT Records          1 Report
PLAT299_ALERT_4_G Atom Site Occupancy Constrained at ............         0.5 Check
              C5      H00D    H5A     H5B     H5C
PLAT302_ALERT_4_G Anion/Solvent/Minor-Residue Disorder (Resd    2)        14% Note
PLAT720_ALERT_4_G Number of Unusual/Non-Standard Labels ..........         20 Note
              I001    I002    S003    S004    C005    C006    C007    H007
              C008    H008    C009    C00A    C00B    C00C    C00D    C00E
              H00E    C00F    H00F    H00D
PLAT860_ALERT_3_G Number of Least-Squares Restraints ............           2 Note
PLAT969_ALERT_5_G The 'Henn et al.' R-Factor-gap value ..........       2.033 Note
              Predicted wR2: Based on SigI**2  7.91 or SHELX Weight 15.27
PLAT978_ALERT_2_G Number C-C Bonds with Positive Residual Density.          2 Info
```

0 **ALERT level A** = Most likely a serious problem - resolve or explain
4 **ALERT level B** = A potentially serious problem, consider carefully
11 **ALERT level C** = Check. Ensure it is not caused by an omission or oversight
11 **ALERT level G** = General information/check it is not something unexpected

3 ALERT type 1 CIF construction/syntax error, inconsistent or missing data

```
13 ALERT type 2 Indicator that the structure model may be wrong or deficient
 4 ALERT type 3 Indicator that the structure quality may be low
 5 ALERT type 4 Improvement, methodology, query or suggestion
 1 ALERT type 5 Informative message, check
```

It is advisable to attempt to resolve as many as possible of the alerts in all categories. Often the minor alerts point to easily fixed oversights, errors and omissions in your CIF or refinement strategy, so attention to these fine details can be worthwhile. In order to resolve some of the more serious problems it may be necessary to carry out additional measurements or structure refinements. However, the purpose of your study may justify the reported deviations and the more serious of these should normally be commented upon in the discussion or experimental section of a paper or in the "special_details" fields of the CIF. checkCIF was carefully designed to identify outliers and unusual parameters, but every test has its limitations and alerts that are not important in a particular case may appear. Conversely, the absence of alerts does not guarantee there are no aspects of the results needing attention. It is up to the individual to critically assess their own results and, if necessary, seek expert advice.

Publication of your CIF in IUCr journals

A basic structural check has been run on your CIF. These basic checks will be run on all CIFs submitted for publication in IUCr journals (*Acta Crystallographica*, *Journal of Applied Crystallography*, *Journal of Synchrotron Radiation*); however, if you intend to submit to *Acta Crystallographica Section C* or *E* or *IUCrData*, you should make sure that full publication checks are run on the final version of your CIF prior to submission.

Publication of your CIF in other journals

Please refer to the *Notes for Authors* of the relevant journal for any special instructions relating to CIF submission.

PLATON version of 04/06/2025; check.def file version of 30/05/2025

Datablock shelx_trans - ellipsoid plot

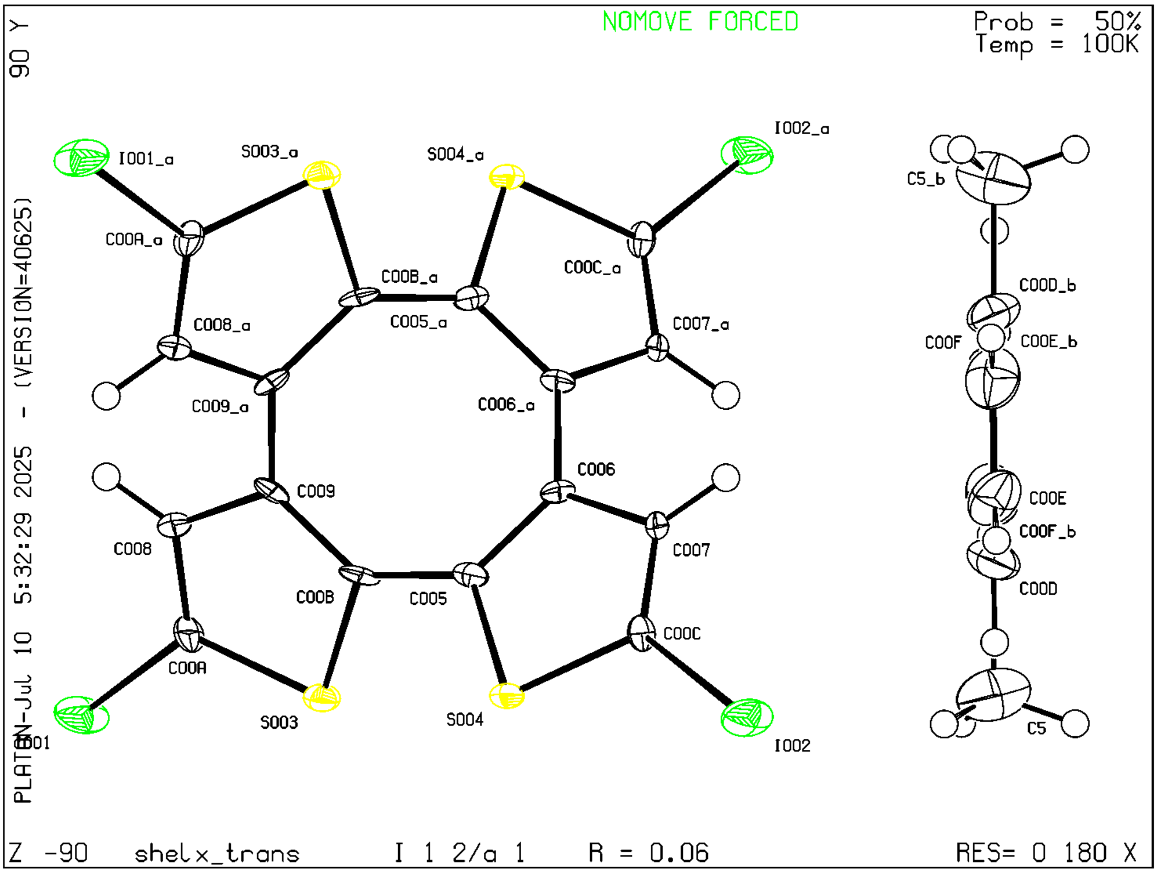

checkCIF/PLATON report

Structure factors have been supplied for datablock(s) shelx_trans

THIS REPORT IS FOR GUIDANCE ONLY. IF USED AS PART OF A REVIEW PROCEDURE FOR PUBLICATION, IT SHOULD NOT REPLACE THE EXPERTISE OF AN EXPERIENCED CRYSTALLOGRAPHIC REFEREE.

No syntax errors found. CIF dictionary Interpreting this report

Datablock: shelx_trans

```
Bond precision:   C-C = 0.0148 A          Wavelength=1.54180

Cell:            a=11.1025(5)     b=13.0763(5)       c=19.1696(16)
                 alpha=90         beta=105.547(7)    gamma=90
Temperature:     293 K

                Calculated                   Reported
Volume          2681.2(3)                    2681.2(3)
Space group     I 2/a                        I 1 2/a 1
Hall group      -I 2ya                       -I 2ya
Moiety formula  C16 H4 I4 S4, C7 H8          C16 H4 I4 S4, C7 H8
Sum formula     C23 H12 I4 S4                C23 H12 I4 S4
Mr              924.17                       924.17
Dx,g cm-3       2.289                        2.289
Z               4                            4
Mu (mm-1)       39.498                       39.498
F000            1704.0                       1704.0
F000'           1709.87
h,k,lmax        13,15,23                     13,15,23
Nref            2450                         2447
Tmin,Tmax                                    1.000,1.000
Tmin'

Correction method= # Reported T Limits: Tmin=1.000 Tmax=1.000
AbsCorr = EMPIRICAL

Data completeness= 0.999          Theta(max)= 68.233

                                                    wR2(reflections)=
R(reflections)= 0.0538( 1571)                       0.1161( 2447)

S = 0.963                  Npar= 150
```

```
The following ALERTS were generated. Each ALERT has the format
       test-name_ALERT_alert-type_alert-level.
Click on the hyperlinks for more details of the test.
```

Alert level C

```
RINTA01_ALERT_3_C  The value of Rint is greater than 0.12
            Rint given   0.131
PLAT053_ALERT_1_C Minimum Crystal Dimension Missing (or Error) ...     Please Check
PLAT054_ALERT_1_C Medium  Crystal Dimension Missing (or Error) ...     Please Check
PLAT055_ALERT_1_C Maximum Crystal Dimension Missing (or Error) ...     Please Check
PLAT244_ALERT_4_C Low    'Solvent' Ueq as Compared to Neighbors of       C00D Check
PLAT260_ALERT_2_C Large Average Ueq of Residue Including      C00D      0.105 Check
PLAT331_ALERT_2_C Small Aver Phenyl C-C Dist C00D     --C00F_b   .       1.37 Ang.
PLAT342_ALERT_3_C Low Bond Precision on  C-C Bonds ...............    0.01483 Ang.
PLAT906_ALERT_3_C Large K Value in the Analysis of Variance ......     10.696 Check
PLAT906_ALERT_3_C Large K Value in the Analysis of Variance ......      2.675 Check
```

Alert level G

```
PLAT002_ALERT_2_G Number of Distance or Angle Restraints on AtSite          3 Note
PLAT020_ALERT_3_G The Value of Rint is Greater Than 0.12 .........      0.131 Report
PLAT172_ALERT_4_G The CIF-Embedded .res File Contains DFIX Records          1 Report
PLAT173_ALERT_4_G The CIF-Embedded .res File Contains DANG Records          1 Report
PLAT174_ALERT_4_G The CIF-Embedded .res File Contains FLAT Records          1 Report
PLAT199_ALERT_1_G Reported _cell_measurement_temperature ..... (K)        293 Check
PLAT200_ALERT_1_G Reported   _diffrn_ambient_temperature ..... (K)        293 Check
PLAT299_ALERT_4_G Atom Site Occupancy Constrained at ............         0.5 Check
              C12     H00D    H12A    H12B    H12C
PLAT302_ALERT_4_G Anion/Solvent/Minor-Residue Disorder (Resd    2)        14% Note
PLAT720_ALERT_4_G Number of Unusual/Non-Standard Labels ..........         20 Note
              I001    I002    S003    S004    C005    C006    C007    C008
              C009    H009    C00A    H00A    C00B    C00C    C00D    C00E
              H00E    C00F    H00F    H00D
PLAT860_ALERT_3_G Number of Least-Squares Restraints ............           3 Note
PLAT912_ALERT_4_G Missing # of FCF Reflections Above STh/L=  0.600          2 Note
PLAT969_ALERT_5_G The 'Henn et al.' R-Factor-gap value ...........      1.237 Note
              Predicted wR2: Based on SigI**2  9.38 or SHELX Weight 12.05
PLAT978_ALERT_2_G Number C-C Bonds with Positive Residual Density.          1 Info
```

```
   0 ALERT level A = Most likely a serious problem - resolve or explain
   0 ALERT level B = A potentially serious problem, consider carefully
  10 ALERT level C = Check. Ensure it is not caused by an omission or oversight
  14 ALERT level G = General information/check it is not something unexpected

   5 ALERT type 1 CIF construction/syntax error, inconsistent or missing data
   4 ALERT type 2 Indicator that the structure model may be wrong or deficient
   6 ALERT type 3 Indicator that the structure quality may be low
   8 ALERT type 4 Improvement, methodology, query or suggestion
   1 ALERT type 5 Informative message, check
```

It is advisable to attempt to resolve as many as possible of the alerts in all categories. Often the minor alerts point to easily fixed oversights, errors and omissions in your CIF or refinement strategy, so attention to these fine details can be worthwhile. In order to resolve some of the more serious problems it may be necessary to carry out additional measurements or structure refinements. However, the purpose of your study may justify the reported deviations and the more serious of these should normally be commented upon in the discussion or experimental section of a paper or in the "special_details" fields of the CIF. checkCIF was carefully designed to identify outliers and unusual parameters, but every test has its limitations and alerts that are not important in a particular case may appear. Conversely, the absence of alerts does not guarantee there are no aspects of the results needing attention. It is up to the individual to critically assess their own results and, if necessary, seek expert advice.

Publication of your CIF in IUCr journals

A basic structural check has been run on your CIF. These basic checks will be run on all CIFs submitted for publication in IUCr journals (*Acta Crystallographica*, *Journal of Applied Crystallography*, *Journal of Synchrotron Radiation*); however, if you intend to submit to *Acta Crystallographica Section C* or *E* or *IUCrData*, you should make sure that full publication checks are run on the final version of your CIF prior to submission.

Publication of your CIF in other journals

Please refer to the *Notes for Authors* of the relevant journal for any special instructions relating to CIF submission.

PLATON version of 04/06/2025; check.def file version of 30/05/2025

Datablock shelx_trans - ellipsoid plot

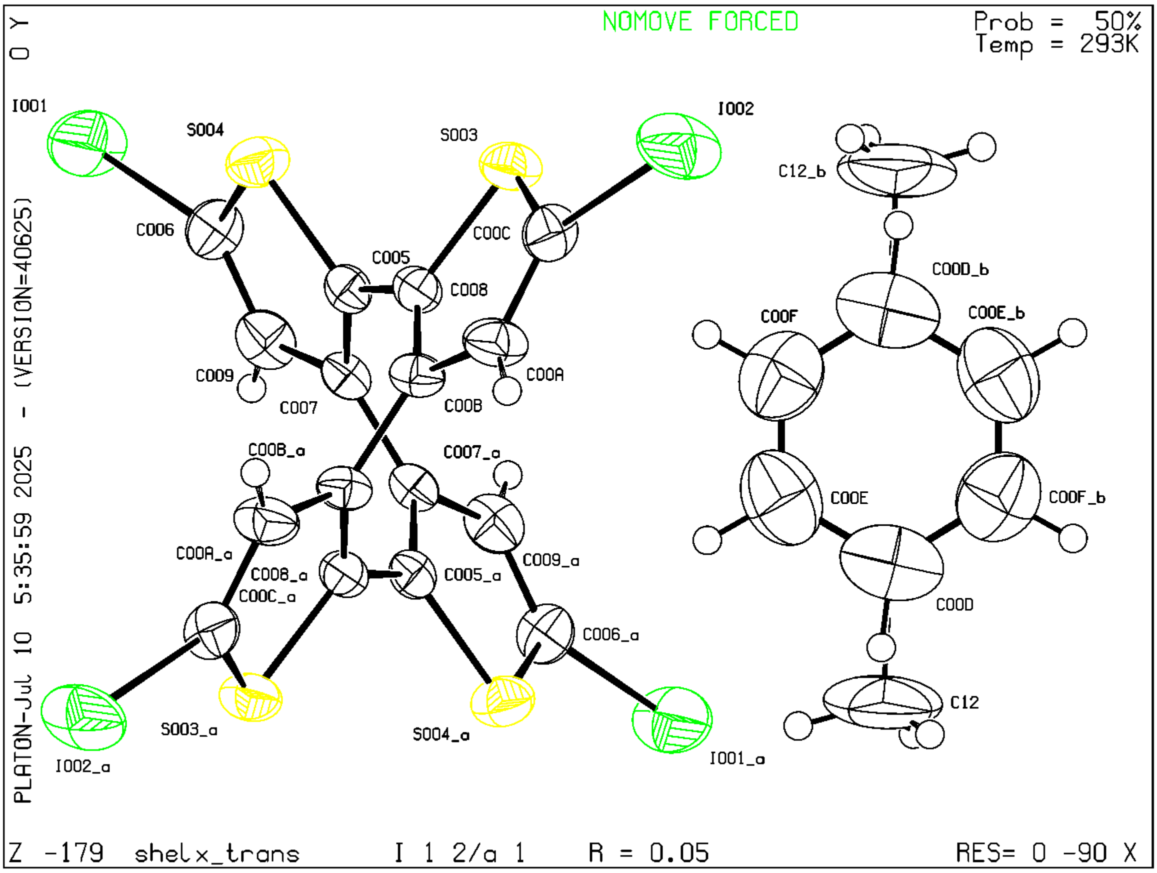

checkCIF/PLATON report

Structure factors have been supplied for datablock(s) shelx

THIS REPORT IS FOR GUIDANCE ONLY. IF USED AS PART OF A REVIEW PROCEDURE FOR PUBLICATION, IT SHOULD NOT REPLACE THE EXPERTISE OF AN EXPERIENCED CRYSTALLOGRAPHIC REFEREE.

No syntax errors found. CIF dictionary Interpreting this report

Datablock: shelx

```
Bond precision:   C-C = 0.0160 A          Wavelength=1.54180

Cell:             a=19.2156(8)     b=13.0505(6)        c=10.7236(4)
                  alpha=90         beta=104.995(7)     gamma=90
Temperature:      100 K

                 Calculated                     Reported
Volume           2597.6(2)                      2597.6(2)
Space group      P 21/c                         P 1 21/c 1
Hall group       -P 2ybc                        -P 2ybc
Moiety formula   C16 H4 I4 S4, C6 H5 Cl         C16 H4 I4 S4, C6 H5 Cl
Sum formula      C22 H9 Cl I4 S4                C22 H9 Cl I4 S4
Mr               944.58                         944.58
Dx,g cm-3        2.415                          2.415
Z                4                              4
Mu (mm-1)        41.716                         41.716
F000             1736.0                         1736.0
F000'            1743.37
h,k,lmax         23,15,12                       23,15,12
Nref             4749                           4743
Tmin,Tmax        0.005,0.015                    0.310,1.000
Tmin'            0.001

Correction method= # Reported T Limits: Tmin=0.310 Tmax=1.000
AbsCorr = EMPIRICAL

Data completeness= 0.999           Theta(max)= 68.244

R(reflections)= 0.0684( 3276)                          wR2(reflections)= 0.1735( 4743)

S = 1.093                  Npar= 297
```

The following ALERTS were generated. Each ALERT has the format
test-name_ALERT_alert-type_alert-level.
Click on the hyperlinks for more details of the test.

Alert level C

```
RINTA01_ALERT_3_C  The value of Rint is greater than 0.12
            Rint given   0.144
PLAT213_ALERT_2_C Atom C00G            has ADP max/min Ratio .....        3.2 prolat
PLAT213_ALERT_2_C Atom C00N            has ADP max/min Ratio .....        3.1 oblate
PLAT223_ALERT_4_C Solv./Anion  Resd 2  H   Ueq(max)/Ueq(min) Range        4.3 Ratio
PLAT250_ALERT_2_C Large U3/U1 Ratio for <U(i,j)> Tensor(Resd    1)        2.9 Note
PLAT250_ALERT_2_C Large U3/U1 Ratio for <U(i,j)> Tensor(Resd    2)        2.6 Note
PLAT331_ALERT_2_C Small Aver Phenyl C-C Dist C00P     --C00T     .       1.37 Ang.
PLAT342_ALERT_3_C Low Bond Precision on  C-C Bonds ..............    0.01605 Ang.
PLAT906_ALERT_3_C Large K Value in the Analysis of Variance ......      7.150 Check
PLAT906_ALERT_3_C Large K Value in the Analysis of Variance ......      2.215 Check
PLAT911_ALERT_3_C Missing FCF Refl Between Thmin & STh/L=    0.600          5 Report
              -13  0 12, -13  1 12, -12  0 12, -12  1 12,  -8  0 12,
PLAT934_ALERT_3_C Number of (Iobs-Icalc)/Sigma(W) > 10 Outliers ..          1 Check
              -17  7  8,
PLAT971_ALERT_2_C Check Calcd Resid. Dens.  0.96Ang From I002            2.40 eA-3
PLAT971_ALERT_2_C Check Calcd Resid. Dens.  0.16Ang From I002            2.40 eA-3
PLAT971_ALERT_2_C Check Calcd Resid. Dens.  0.97Ang From I001            2.33 eA-3
PLAT971_ALERT_2_C Check Calcd Resid. Dens.  0.12Ang From I001            2.13 eA-3
PLAT971_ALERT_2_C Check Calcd Resid. Dens.  0.04Ang From I003            2.07 eA-3
PLAT971_ALERT_2_C Check Calcd Resid. Dens.  0.18Ang From I004            1.89 eA-3
PLAT971_ALERT_2_C Check Calcd Resid. Dens.  0.91Ang From I003            1.68 eA-3
PLAT971_ALERT_2_C Check Calcd Resid. Dens.  0.95Ang From I002            1.65 eA-3
PLAT971_ALERT_2_C Check Calcd Resid. Dens.  1.00Ang From I003            1.62 eA-3
PLAT971_ALERT_2_C Check Calcd Resid. Dens.  0.97Ang From I003            1.61 eA-3
PLAT971_ALERT_2_C Check Calcd Resid. Dens.  0.95Ang From I001            1.51 eA-3
PLAT972_ALERT_2_C Check Calcd Resid. Dens.  0.82Ang From I002           -2.27 eA-3
PLAT972_ALERT_2_C Check Calcd Resid. Dens.  0.88Ang From I002           -2.25 eA-3
PLAT972_ALERT_2_C Check Calcd Resid. Dens.  0.85Ang From I003           -2.07 eA-3
PLAT972_ALERT_2_C Check Calcd Resid. Dens.  0.83Ang From I001           -2.07 eA-3
PLAT972_ALERT_2_C Check Calcd Resid. Dens.  0.80Ang From I001           -2.06 eA-3
PLAT972_ALERT_2_C Check Calcd Resid. Dens.  0.80Ang From I004           -1.93 eA-3
PLAT972_ALERT_2_C Check Calcd Resid. Dens.  0.89Ang From I003           -1.88 eA-3
PLAT972_ALERT_2_C Check Calcd Resid. Dens.  0.74Ang From I001           -1.66 eA-3
PLAT972_ALERT_2_C Check Calcd Resid. Dens.  0.85Ang From I004           -1.58 eA-3
PLAT972_ALERT_2_C Check Calcd Resid. Dens.  1.54Ang From I002           -1.58 eA-3
PLAT972_ALERT_2_C Check Calcd Resid. Dens.  0.69Ang From I002           -1.58 eA-3
PLAT977_ALERT_2_C Check Negative Difference Density on H00U      .      -0.32 eA-3
```

Alert level G

```
PLAT002_ALERT_2_G Number of Distance or Angle Restraints on AtSite          4 Note
PLAT003_ALERT_2_G Number of Uiso or U(i,j)  Restrained non-H-Atoms          1 Report
PLAT020_ALERT_3_G The Value of Rint is Greater Than 0.12 .........      0.144 Report
PLAT172_ALERT_4_G The CIF-Embedded .res File Contains DFIX Records          2 Report
PLAT174_ALERT_4_G The CIF-Embedded .res File Contains FLAT Records          1 Report
PLAT186_ALERT_4_G The CIF-Embedded .res File Contains ISOR Records          1 Report
PLAT300_ALERT_4_G Atom Site Occupancy of Cl1       Constrained at        0.78 Check
PLAT300_ALERT_4_G Atom Site Occupancy of Cl2       Constrained at        0.22 Check
PLAT300_ALERT_4_G Atom Site Occupancy of H00P      Constrained at        0.78 Check
```

```
PLAT300_ALERT_4_G Atom Site Occupancy of H00U       Constrained at       0.22 Check
PLAT302_ALERT_4_G Anion/Solvent/Minor-Residue Disorder (Resd    2)        14% Note
PLAT720_ALERT_4_G Number of Unusual/Non-Standard Labels .........          40 Note
              I001    I002    I003    I004    S005    S006    S007    S008
              C00A    C00B    C00C    C00D    C00E    H00E    C00F    H00F
              C00G    H00G    C00H    C00I    C00J    H00J    C00K    C00L
              C00M    C00N    C00O    C00P    C00Q    H00Q    C00R    C00S
              H00S    C00T    H00T    C00U    C00V    H00V    H00U    H00P
PLAT860_ALERT_3_G Number of Least-Squares Restraints ............          11 Note
PLAT883_ALERT_1_G Absent Datum for _atom_sites_solution_primary ..     Please Do !
PLAT910_ALERT_3_G Missing FCF Reflection(s) Below Theta(Min)[Deg]=       4.14 Note
                1  0  0,
PLAT969_ALERT_5_G The 'Henn et al.' R-Factor-gap value ..........      1.741 Note
              Predicted wR2: Based on SigI**2  9.97 or SHELX Weight 15.87
PLAT978_ALERT_2_G Number C-C Bonds with Positive Residual Density.          0 Info
```

```
   0 ALERT level A = Most likely a serious problem - resolve or explain
   0 ALERT level B = A potentially serious problem, consider carefully
  35 ALERT level C = Check. Ensure it is not caused by an omission or oversight
  17 ALERT level G = General information/check it is not something unexpected

   1 ALERT type 1 CIF construction/syntax error, inconsistent or missing data
  31 ALERT type 2 Indicator that the structure model may be wrong or deficient
   9 ALERT type 3 Indicator that the structure quality may be low
  10 ALERT type 4 Improvement, methodology, query or suggestion
   1 ALERT type 5 Informative message, check
```

It is advisable to attempt to resolve as many as possible of the alerts in all categories. Often the minor alerts point to easily fixed oversights, errors and omissions in your CIF or refinement strategy, so attention to these fine details can be worthwhile. In order to resolve some of the more serious problems it may be necessary to carry out additional measurements or structure refinements. However, the purpose of your study may justify the reported deviations and the more serious of these should normally be commented upon in the discussion or experimental section of a paper or in the "special_details" fields of the CIF. checkCIF was carefully designed to identify outliers and unusual parameters, but every test has its limitations and alerts that are not important in a particular case may appear. Conversely, the absence of alerts does not guarantee there are no aspects of the results needing attention. It is up to the individual to critically assess their own results and, if necessary, seek expert advice.

Publication of your CIF in IUCr journals

A basic structural check has been run on your CIF. These basic checks will be run on all CIFs submitted for publication in IUCr journals (*Acta Crystallographica*, *Journal of Applied Crystallography*, *Journal of Synchrotron Radiation*); however, if you intend to submit to *Acta Crystallographica Section C* or *E* or *IUCrData*, you should make sure that full publication checks are run on the final version of your CIF prior to submission.

Publication of your CIF in other journals

Please refer to the *Notes for Authors* of the relevant journal for any special instructions relating to CIF submission.

PLATON version of 04/06/2025; check.def file version of 30/05/2025

Datablock shelx - ellipsoid plot

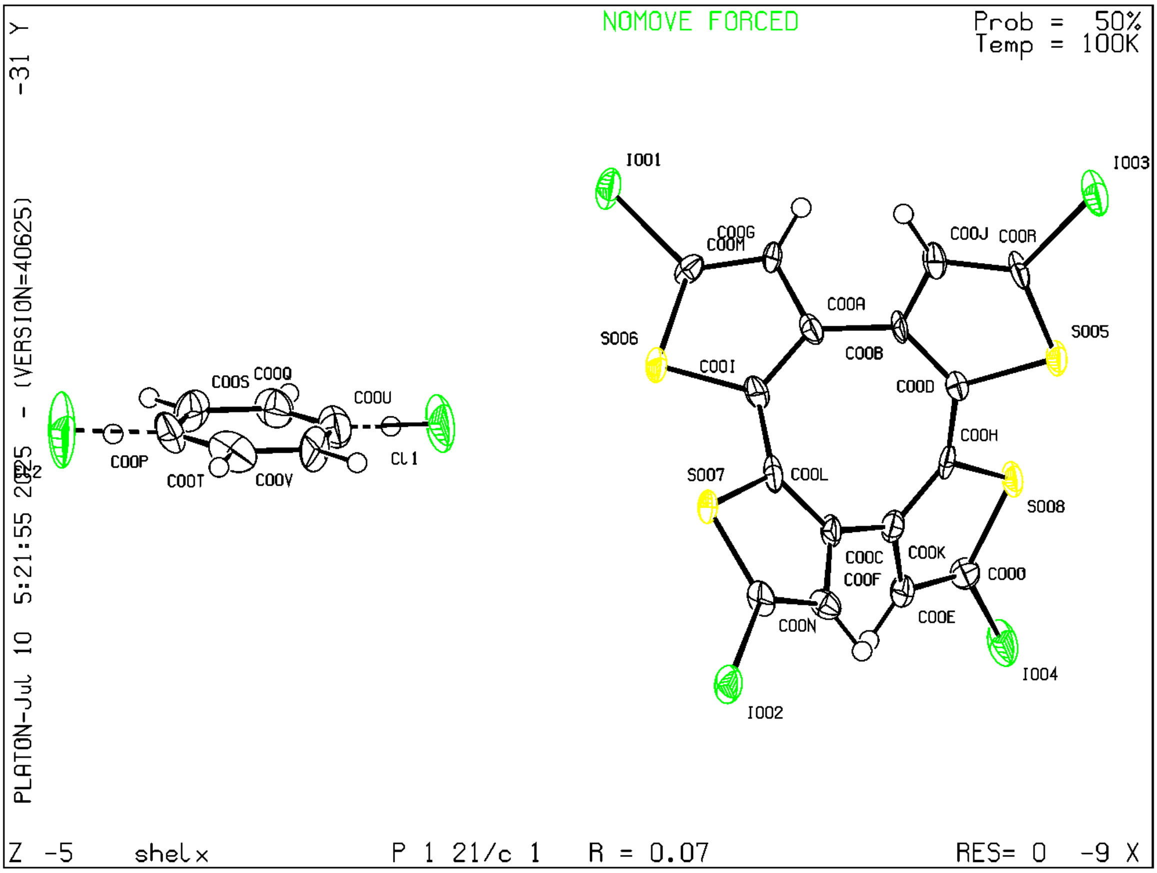

checkCIF/PLATON report

Structure factors have been supplied for datablock(s) shelx_trans

THIS REPORT IS FOR GUIDANCE ONLY. IF USED AS PART OF A REVIEW PROCEDURE FOR PUBLICATION, IT SHOULD NOT REPLACE THE EXPERTISE OF AN EXPERIENCED CRYSTALLOGRAPHIC REFEREE.

No syntax errors found. CIF dictionary Interpreting this report

Datablock: shelx_trans

```
Bond precision:   C-C = 0.0107 A          Wavelength=1.54180

Cell:            a=11.0401(4)     b=13.1470(5)       c=19.1636(15)
                 alpha=90         beta=105.497(6)    gamma=90
Temperature:     293 K

                Calculated                    Reported
Volume          2680.4(3)                     2680.4(3)
Space group     I 2/a                         I 1 2/a 1
Hall group      -I 2ya                        -I 2ya
Moiety formula  C16 H4 I4 S4, C6 H5 Cl        C16 H4 I4 S4, C6 H5 Cl
Sum formula     C22 H9 Cl I4 S4               C22 H9 Cl I4 S4
Mr              944.58                        944.58
Dx,g cm-3       2.341                         2.341
Z               4                             4
Mu (mm-1)       40.427                        40.428
F000            1736.0                        1736.0
F000'           1743.37
h,k,lmax        13,15,23                      13,15,23
Nref            2454                          2452
Tmin,Tmax                                     0.132,1.000
Tmin'

Correction method= # Reported T Limits: Tmin=0.132 Tmax=1.000
AbsCorr = EMPIRICAL

Data completeness= 0.999          Theta(max)= 68.206

R(reflections)= 0.0534( 1976)                     wR2(reflections)=
                                                  0.1430( 2452)
S = 1.023                    Npar= 149
```

The following ALERTS were generated. Each ALERT has the format
test-name_ALERT_alert-type_alert-level.
Click on the hyperlinks for more details of the test.

Alert level C

```
PLAT053_ALERT_1_C Minimum Crystal Dimension Missing (or Error) ...     Please Check
PLAT054_ALERT_1_C Medium  Crystal Dimension Missing (or Error) ...     Please Check
PLAT055_ALERT_1_C Maximum Crystal Dimension Missing (or Error) ...     Please Check
PLAT234_ALERT_4_C Large Hirshfeld Difference C00F     --C00G     .       0.17 Ang.
PLAT244_ALERT_4_C Low    'Solvent' Ueq as Compared to Neighbors of       C00F Check
PLAT260_ALERT_2_C Large Average Ueq of Residue Including      Cl00      0.119 Check
PLAT342_ALERT_3_C Low Bond Precision on  C-C Bonds ...............    0.01067 Ang.
PLAT906_ALERT_3_C Large K Value in the Analysis of Variance ......      4.625 Check
PLAT972_ALERT_2_C Check Calcd Resid. Dens.  0.86Ang From I001           -1.51 eA-3
```

Alert level G

```
PLAT002_ALERT_2_G Number of Distance or Angle Restraints on AtSite          5 Note
PLAT172_ALERT_4_G The CIF-Embedded .res File Contains DFIX Records          2 Report
PLAT173_ALERT_4_G The CIF-Embedded .res File Contains DANG Records          1 Report
PLAT174_ALERT_4_G The CIF-Embedded .res File Contains FLAT Records          1 Report
PLAT199_ALERT_1_G Reported _cell_measurement_temperature ..... (K)        293 Check
PLAT200_ALERT_1_G Reported   _diffrn_ambient_temperature ..... (K)        293 Check
PLAT299_ALERT_4_G Atom Site Occupancy Constrained at ............         0.5 Check
              Cl00    H00F
PLAT302_ALERT_4_G Anion/Solvent/Minor-Residue Disorder (Resd    2)        14% Note
PLAT720_ALERT_4_G Number of Unusual/Non-Standard Labels ..........         21 Note
              I001    I002    S003    S004    C005    C006    C007    C008
              C009    H009    C00A    H00A    C00B    C00C    Cl00    C00E
              H00E    C00F    C00G    H00G    H00F
PLAT860_ALERT_3_G Number of Least-Squares Restraints ............           6 Note
PLAT912_ALERT_4_G Missing # of FCF Reflections Above STh/L=  0.600          1 Note
PLAT969_ALERT_5_G The 'Henn et al.' R-Factor-gap value ..........       2.137 Note
              Predicted wR2: Based on SigI**2  6.69 or SHELX Weight 13.98
PLAT978_ALERT_2_G Number C-C Bonds with Positive Residual Density.          0 Info
```

0 **ALERT level A** = Most likely a serious problem - resolve or explain
0 **ALERT level B** = A potentially serious problem, consider carefully
9 **ALERT level C** = Check. Ensure it is not caused by an omission or oversight
13 **ALERT level G** = General information/check it is not something unexpected

5 ALERT type 1 CIF construction/syntax error, inconsistent or missing data
4 ALERT type 2 Indicator that the structure model may be wrong or deficient
3 ALERT type 3 Indicator that the structure quality may be low
9 ALERT type 4 Improvement, methodology, query or suggestion
1 ALERT type 5 Informative message, check

It is advisable to attempt to resolve as many as possible of the alerts in all categories. Often the minor alerts point to easily fixed oversights, errors and omissions in your CIF or refinement strategy, so attention to these fine details can be worthwhile. In order to resolve some of the more serious problems it may be necessary to carry out additional measurements or structure refinements. However, the purpose of your study may justify the reported deviations and the more serious of these should normally be commented upon in the discussion or experimental section of a paper or in the "special_details" fields of the CIF. checkCIF was carefully designed to identify outliers and unusual parameters, but every test has its limitations and alerts that are not important in a particular case may appear. Conversely, the absence of alerts does not guarantee there are no aspects of the results needing attention. It is up to the individual to critically assess their own results and, if necessary, seek expert advice.

Publication of your CIF in IUCr journals

A basic structural check has been run on your CIF. These basic checks will be run on all CIFs submitted for publication in IUCr journals (*Acta Crystallographica*, *Journal of Applied Crystallography*, *Journal of Synchrotron Radiation*); however, if you intend to submit to *Acta Crystallographica Section C* or *E* or *IUCrData*, you should make sure that full publication checks are run on the final version of your CIF prior to submission.

Publication of your CIF in other journals

Please refer to the *Notes for Authors* of the relevant journal for any special instructions relating to CIF submission.

PLATON version of 04/06/2025; check.def file version of 30/05/2025

Datablock shelx_trans - ellipsoid plot

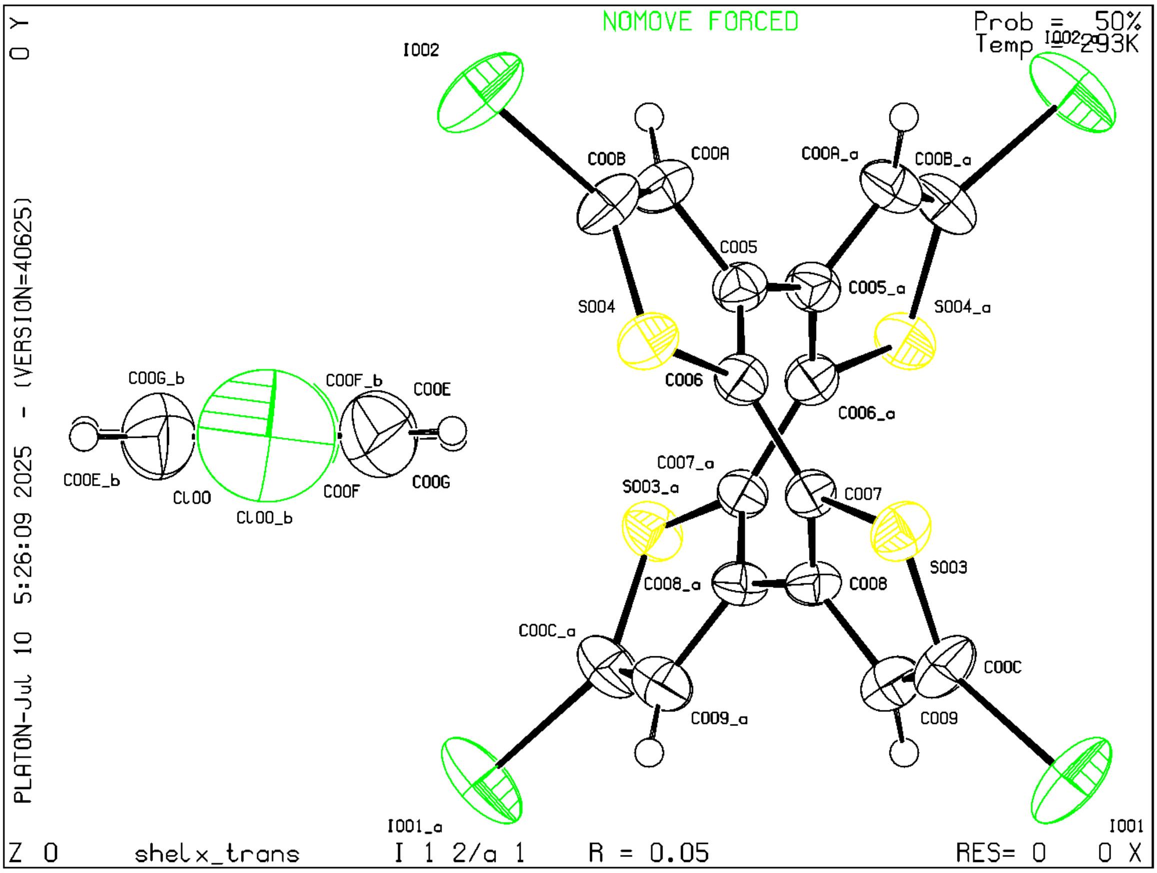

checkCIF/PLATON report

Structure factors have been supplied for datablock(s) shelx_trans

THIS REPORT IS FOR GUIDANCE ONLY. IF USED AS PART OF A REVIEW PROCEDURE FOR PUBLICATION, IT SHOULD NOT REPLACE THE EXPERTISE OF AN EXPERIENCED CRYSTALLOGRAPHIC REFEREE.

No syntax errors found.   CIF dictionary   Interpreting this report

Datablock: shelx_trans

```
Bond precision:   C-C = 0.0086 A          Wavelength=1.54180

Cell:             a=10.9873(2)     b=12.9319(8)       c=19.0732(10)
                  alpha=90         beta=105.739(4)    gamma=90
Temperature:      293 K

                  Calculated                 Reported
Volume            2608.4(2)                  2608.4(2)
Space group       I 2/a                      I 1 2/a 1
Hall group        -I 2ya                     -I 2ya
Moiety formula    C16 H4 I4 S4, C7 H8 O      C16 H4 I4 S4, C7 H8 O
Sum formula       C23 H12 I4 O S4            C23 H12 I4 O S4
Mr                940.17                     940.17
Dx,g cm-3         2.394                      2.394
Z                 4                          4
Mu (mm-1)         40.647                     40.646
F000              1736.0                     1736.0
F000'             1742.00
h,k,lmax          13,15,22                   13,15,22
Nref              2395                       2392
Tmin,Tmax                                    0.275,1.000
Tmin'

Correction method= # Reported T Limits: Tmin=0.275 Tmax=1.000
AbsCorr = EMPIRICAL

Data completeness= 0.999          Theta(max)= 68.204

R(reflections)= 0.0532( 2085)          wR2(reflections)=
                                       0.1389( 2392)
S = 1.059                 Npar= 170
```

The following ALERTS were generated. Each ALERT has the format
test-name_ALERT_alert-type_alert-level.
Click on the hyperlinks for more details of the test.

Alert level B

```
PLAT971_ALERT_2_B Check Calcd Resid. Dens.  0.86Ang From I002          2.88 eA-3
PLAT971_ALERT_2_B Check Calcd Resid. Dens.  0.97Ang From I001          2.52 eA-3
```

Alert level C

```
PLAT053_ALERT_1_C Minimum Crystal Dimension Missing (or Error) ...     Please Check
PLAT054_ALERT_1_C Medium  Crystal Dimension Missing (or Error) ...     Please Check
PLAT055_ALERT_1_C Maximum Crystal Dimension Missing (or Error) ...     Please Check
PLAT342_ALERT_3_C Low Bond Precision on  C-C Bonds ...............    0.00856 Ang.
PLAT906_ALERT_3_C Large K Value in the Analysis of Variance ......      3.721 Check
PLAT971_ALERT_2_C Check Calcd Resid. Dens.  0.81Ang From I002          1.83 eA-3
PLAT971_ALERT_2_C Check Calcd Resid. Dens.  0.91Ang From I001          1.65 eA-3
PLAT971_ALERT_2_C Check Calcd Resid. Dens.  0.96Ang From I002          1.57 eA-3
PLAT971_ALERT_2_C Check Calcd Resid. Dens.  0.87Ang From I001          1.55 eA-3
PLAT972_ALERT_2_C Check Calcd Resid. Dens.  0.85Ang From I001         -1.98 eA-3
PLAT972_ALERT_2_C Check Calcd Resid. Dens.  0.89Ang From I002         -1.96 eA-3
PLAT972_ALERT_2_C Check Calcd Resid. Dens.  0.92Ang From I002         -1.75 eA-3
PLAT972_ALERT_2_C Check Calcd Resid. Dens.  0.96Ang From I001         -1.55 eA-3
PLAT977_ALERT_2_C Check Negative Difference Density on H7C       .      -0.54 eA-3
```

Alert level G

```
PLAT003_ALERT_2_G Number of Uiso or U(i,j)  Restrained non-H-Atoms          9 Report
PLAT083_ALERT_2_G SHELXL Second Parameter in WGHT  Unusually Large       9.82 Why ?
PLAT178_ALERT_4_G The CIF-Embedded .res File Contains SIMU Records          1 Report
PLAT186_ALERT_4_G The CIF-Embedded .res File Contains ISOR Records          1 Report
PLAT188_ALERT_3_G A Non-default SIMU Restraint Value has been used     0.0010 Report
PLAT199_ALERT_1_G Reported _cell_measurement_temperature ..... (K)        293 Check
PLAT200_ALERT_1_G Reported   _diffrn_ambient_temperature ..... (K)        293 Check
PLAT299_ALERT_4_G Atom Site Occupancy Constrained at ............         0.5 Check
              O       C1A     C2      C5      C7      C00F    C00E    C11
              H1A     H2      H00F    H00E    H7A     H7B     H7C     H11
PLAT302_ALERT_4_G Anion/Solvent/Minor-Residue Disorder (Resd    2)       100% Note
PLAT720_ALERT_4_G Number of Unusual/Non-Standard Labels ..........         18 Note
              I001    I002    S003    S004    C005    C006    C007    C008
              C009    C00A    C00B    H00B    C00C    H00C    C00F    H00F
              C00E    H00E
PLAT764_ALERT_4_G Overcomplete CIF Bond List Detected (Rep/Expd) .       1.13 Ratio
PLAT773_ALERT_2_G Check long C-C Bond in CIF: C1A      --C00F            1.88 Ang.
PLAT774_ALERT_1_G Check   X-Y Bond in CIF: O        --C7        ..       5.34 Ang.
PLAT779_ALERT_4_G Suspect or Irrelevant (Bond) Angle(s) in CIF ...      11.00 Deg.
              C5   -O    -C7      1_555   1_555   2_656 ......     #   29 Check
PLAT779_ALERT_4_G Suspect or Irrelevant (Bond) Angle(s) in CIF ...      15.60 Deg.
              C5   -O    -C7      2_656   1_555   2_656 ......     #   31 Check
PLAT779_ALERT_4_G Suspect or Irrelevant (Bond) Angle(s) in CIF ...       5.30 Deg.
              C5   -O    -C5      1_555   1_555   2_656 ......     #   32 Check
PLAT779_ALERT_4_G Suspect or Irrelevant (Bond) Angle(s) in CIF ...      30.10 Deg.
              C2   -C1A  -C00F    1_555   1_555   2_656 ......     #   51 Check
PLAT789_ALERT_4_G Atoms with Negative _atom_site_disorder_group  #         16 Check
```

```
PLAT822_ALERT_4_G CIF-embedded .res Contains Negative PART Numbers          1 Check
PLAT860_ALERT_3_G Number of Least-Squares Restraints ............          54 Note
PLAT883_ALERT_1_G Absent Datum for _atom_sites_solution_primary ..     Please Do !
PLAT912_ALERT_4_G Missing # of FCF Reflections Above STh/L=  0.600          2 Note
PLAT969_ALERT_5_G The 'Henn et al.' R-Factor-gap value ..........       2.399 Note
              Predicted wR2: Based on SigI**2  5.79 or SHELX Weight 13.11
PLAT978_ALERT_2_G Number C-C Bonds with Positive Residual Density.          0 Info
```

```
   0 ALERT level A = Most likely a serious problem - resolve or explain
   2 ALERT level B = A potentially serious problem, consider carefully
  14 ALERT level C = Check. Ensure it is not caused by an omission or oversight
  24 ALERT level G = General information/check it is not something unexpected

   7 ALERT type 1 CIF construction/syntax error, inconsistent or missing data
  15 ALERT type 2 Indicator that the structure model may be wrong or deficient
   4 ALERT type 3 Indicator that the structure quality may be low
  13 ALERT type 4 Improvement, methodology, query or suggestion
   1 ALERT type 5 Informative message, check
```

It is advisable to attempt to resolve as many as possible of the alerts in all categories. Often the minor alerts point to easily fixed oversights, errors and omissions in your CIF or refinement strategy, so attention to these fine details can be worthwhile. In order to resolve some of the more serious problems it may be necessary to carry out additional measurements or structure refinements. However, the purpose of your study may justify the reported deviations and the more serious of these should normally be commented upon in the discussion or experimental section of a paper or in the "special_details" fields of the CIF. checkCIF was carefully designed to identify outliers and unusual parameters, but every test has its limitations and alerts that are not important in a particular case may appear. Conversely, the absence of alerts does not guarantee there are no aspects of the results needing attention. It is up to the individual to critically assess their own results and, if necessary, seek expert advice.

Publication of your CIF in IUCr journals

A basic structural check has been run on your CIF. These basic checks will be run on all CIFs submitted for publication in IUCr journals (*Acta Crystallographica*, *Journal of Applied Crystallography*, *Journal of Synchrotron Radiation*); however, if you intend to submit to *Acta Crystallographica Section C* or *E* or *IUCrData*, you should make sure that full publication checks are run on the final version of your CIF prior to submission.

Publication of your CIF in other journals

Please refer to the *Notes for Authors* of the relevant journal for any special instructions relating to CIF submission.

PLATON version of 04/06/2025; check.def file version of 30/05/2025

Datablock shelx_trans - ellipsoid plot

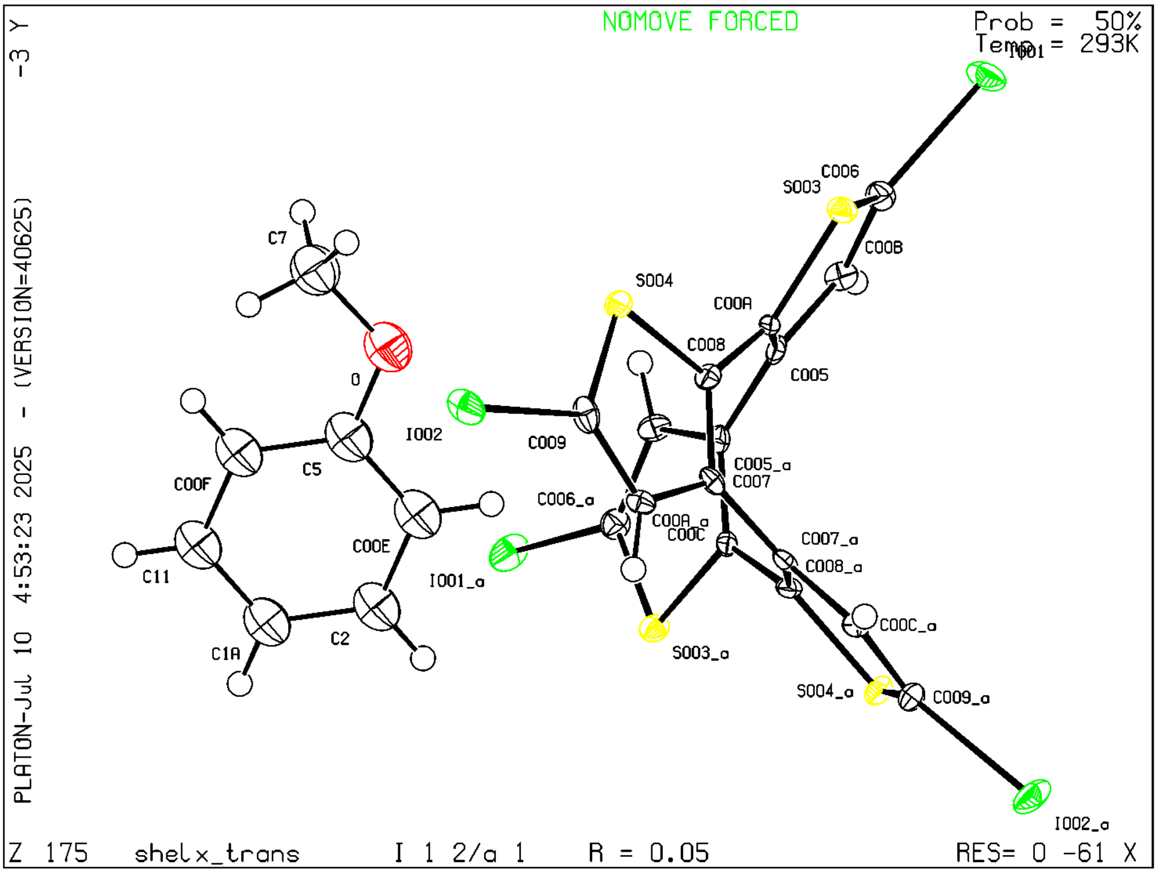

Supplement: Supplementary file 2 — Supporting Information [file CHEM-31-e02872-s001.zip › CheckCIF.pdf]
