# Supplementary material for: Porous Organic Frameworks Utilizing Halogen···Halogen Interactions of X4–tetra[2,3]Thienylene (X = Br, I): Guest Dynamics and Dielectric Response
Source: Chemistry. 2025 Nov 10;31(71):e02872. doi: 10.1002/chem.202502872 (PMC12734654; doi:10.1002/chem.202502872)

# checkCIF/PLATON report

Structure factors have been supplied for datablock(s) shelx_sq

THIS REPORT IS FOR GUIDANCE ONLY. IF USED AS PART OF A REVIEW PROCEDURE FOR PUBLICATION, IT SHOULD NOT REPLACE THE EXPERTISE OF AN EXPERIENCED CRYSTALLOGRAPHIC REFEREE.

No syntax errors found. CIF dictionary Interpreting this report

# Datablock: shelx_sq

---

Bond precision: C-C = 0.0169 A Wavelength=1.54180

| | | | |
|---|---|---|---|
| Cell: | a=10.3980(8) | b=13.1641(10) | c=25.809(2) |
| | alpha=90 | beta=90 | gamma=90 |
| Temperature: | 100 K | | |

| | Calculated | Reported |
|---|---|---|
| Volume | 3532.7(5) | 3532.7(5) |
| Space group | I b a m | I b a m |
| Hall group | -I 2 2c | -I 2 2c |
| Moiety formula | C16 H4 I4 S4, 2(C6 H6) [+ solvent] | C16 H4 I4 S4, 2(C6 H6), 1[] |
| Sum formula | C28 H16 I4 S4 [+ solvent] | C28 H16 I4 S4 |
| Mr | 988.25 | 988.25 |
| Dx,g cm-3 | 1.858 | 1.858 |
| Z | 4 | 4 |
| Mu (mm-1) | 30.029 | 30.029 |
| F000 | 1840.0 | 1840.0 |
| F000' | 1845.92 | |
| h,k,lmax | 12,15,31 | 12,15,30 |
| Nref | 1661 | 1661 |
| Tmin,Tmax | 0.006,0.050 | 0.262,0.993 |
| Tmin' | 0.001 | |

Correction method= # Reported T Limits: Tmin=0.262 Tmax=0.993
AbsCorr = EMPIRICAL

Data completeness= 1.000 Theta(max)= 68.095

R(reflections)= 0.0582( 1306) wR2(reflections)= 0.1642( 1661)

S = 1.111 Npar= 87

The following ALERTS were generated. Each ALERT has the format
**test-name_ALERT_alert-type_alert-level.**
Click on the hyperlinks for more details of the test.

## Alert level C

```
RINTA01_ALERT_3_C  The value of Rint is greater than 0.12
            Rint given   0.128
PLAT243_ALERT_4_C High   'Solvent' Ueq as Compared to Neighbors of       C00B Check
PLAT342_ALERT_3_C Low Bond Precision on  C-C Bonds ...............     0.0169 Ang.
PLAT906_ALERT_3_C Large K Value in the Analysis of Variance ......      3.297 Check
PLAT971_ALERT_2_C Check Calcd Resid. Dens.  0.91Ang From I001            2.43 eA-3
```

## Alert level G

```
PLAT002_ALERT_2_G Number of Distance or Angle Restraints on AtSite          5 Note
PLAT003_ALERT_2_G Number of Uiso or U(i,j)  Restrained non-H-Atoms          3 Report
PLAT020_ALERT_3_G The Value of Rint is Greater Than 0.12 .........      0.128 Report
PLAT042_ALERT_1_G Calc. and Reported MoietyFormula Strings  Differ     Please Check
              Calc: C16 H4 I4 S4, 2(C6 H6)
              Rep.: C16 H4 I4 S4, 2(C6 H6), 1[]
PLAT083_ALERT_2_G SHELXL Second Parameter in WGHT  Unusually Large      46.82 Why ?
PLAT172_ALERT_4_G The CIF-Embedded .res File Contains DFIX Records          2 Report
PLAT186_ALERT_4_G The CIF-Embedded .res File Contains ISOR Records          1 Report
PLAT605_ALERT_4_G Largest Solvent Accessible VOID in the Structure        179 A**3
PLAT720_ALERT_4_G Number of Unusual/Non-Standard Labels ..........         17 Note
              I001    S002    C003    H003    C004    C005    C006    C007
              H007    C008    H008    C009    H009    C00A    H00A    C00B
              H00B
PLAT764_ALERT_4_G Overcomplete CIF Bond List Detected (Rep/Expd) .       1.15 Ratio
PLAT860_ALERT_3_G Number of Least-Squares Restraints .............         22 Note
PLAT869_ALERT_4_G ALERTS Related to the Use of SQUEEZE  Suppressed          ! Info
PLAT883_ALERT_1_G Absent Datum for _atom_sites_solution_primary ..     Please Do !
PLAT909_ALERT_3_G Percentage of I>2sig(I) Data at Theta(Max) Still        59% Note
PLAT969_ALERT_5_G The 'Henn et al.' R-Factor-gap value ...........      2.591 Note
              Predicted wR2: Based on SigI**2  6.34 or SHELX Weight 14.82
PLAT978_ALERT_2_G Number C-C Bonds with Positive Residual Density.          1 Info
```

- 0 **ALERT level A** = Most likely a serious problem - resolve or explain
- 0 **ALERT level B** = A potentially serious problem, consider carefully
- 5 **ALERT level C** = Check. Ensure it is not caused by an omission or oversight
- 16 **ALERT level G** = General information/check it is not something unexpected

- 2 ALERT type 1 CIF construction/syntax error, inconsistent or missing data
- 5 ALERT type 2 Indicator that the structure model may be wrong or deficient
- 6 ALERT type 3 Indicator that the structure quality may be low
- 7 ALERT type 4 Improvement, methodology, query or suggestion
- 1 ALERT type 5 Informative message, check

It is advisable to attempt to resolve as many as possible of the alerts in all categories. Often the minor alerts point to easily fixed oversights, errors and omissions in your CIF or refinement strategy, so attention to these fine details can be worthwhile. In order to resolve some of the more serious problems it may be necessary to carry out additional measurements or structure refinements. However, the purpose of your study may justify the reported deviations and the more serious of these should normally be commented upon in the discussion or experimental section of a paper or in the "special_details" fields of the CIF. checkCIF was carefully designed to identify outliers and unusual parameters, but every test has its limitations and alerts that are not important in a particular case may appear. Conversely, the absence of alerts does not guarantee there are no aspects of the results needing attention. It is up to the individual to critically assess their own results and, if necessary, seek expert advice.

**Publication of your CIF in IUCr journals**

A basic structural check has been run on your CIF. These basic checks will be run on all CIFs submitted for publication in IUCr journals (*Acta Crystallographica*, *Journal of Applied Crystallography*, *Journal of Synchrotron Radiation*); however, if you intend to submit to *Acta Crystallographica Section C* or *E* or *IUCrData*, you should make sure that full publication checks are run on the final version of your CIF prior to submission.

**Publication of your CIF in other journals**

Please refer to the *Notes for Authors* of the relevant journal for any special instructions relating to CIF submission.

---

**PLATON version of 04/06/2025; check.def file version of 30/05/2025**

**Datablock shelx_sq** - ellipsoid plot

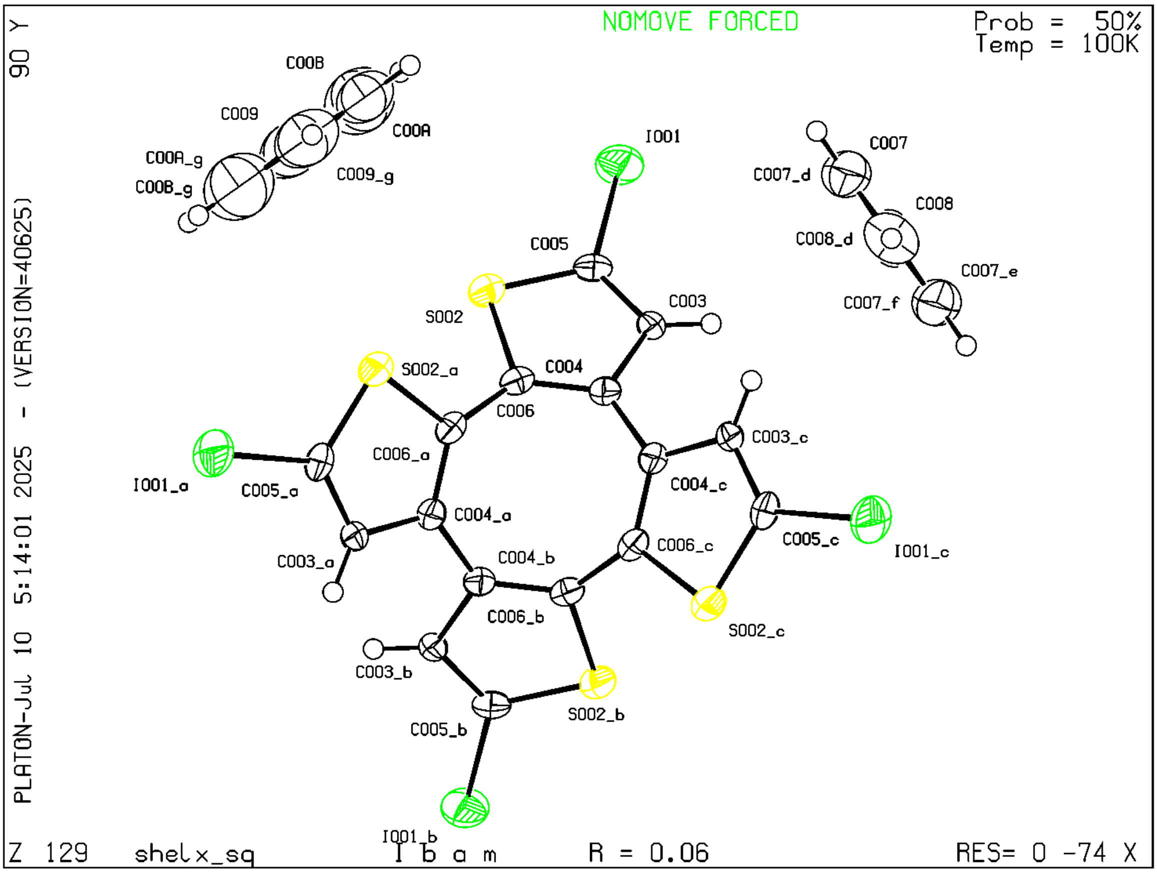

Supplement: Supplementary file 2 — Supporting Information [file CHEM-31-e02872-s001.zip › I_2Bz_100K.pdf]
